# Supplementary material for: Barriers to childhood asthma care in sub-Saharan Africa: a multicountry qualitative study with children and their caregivers
Source: BMJ Open. 2023 Sep 1;13(9):e070784. doi: 10.1136/bmjopen-2022-070784 (PMC10476107; doi:10.1136/bmjopen-2022-070784)
Supplement: Supplementary data [file bmjopen-2022-070784supp001.pdf]

# ACACIA Breathing Survey

**QR code label or Participant ID:**

______________________________

## Introduction

We are a group of researchers interested in healthy breathing. In the questions below we will ask if you sometimes make a noise when you breathe, which is called wheezing. Wheezing is a whistling sound from the chest that some people make when they breathe.

## Section 1: Personal Details

1. **What is your name?** ______________________________________________

2. **Today's date:** DAY / MONTH / YEAR

3. **School:** ______________________________________________

4. **Are you male or female?**
   - ☐ Male
   - ☐ Female

5. **How old are you?** _________

6. **What is your date of birth?** DAY / MONTH / YEAR

7. **Which school class or school year are you in?** ______________________________

8. **How would you describe your ethnicity?**
   - ☐ Black
   - ☐ White
   - ☐ East Asian, such as Chinese, Japanese, or Korean
   - ☐ South Asian, such as Indian, or Bangladeshi
   - ☐ Mixed race, please specify: ______________________
   - ☐ Other, please specify: ___________________________



Naidoo KL, *et al. BMJ Open* 2023; 13:e070784. doi: 10.1136/bmjopen-2022-070784

acacia achieving control of asthma in children in africa

UNIVERSITY OF KWAZULU-NATAL INYUVESI YAKWAZULU-NATALI

## Section 2: Breathing

1. **Have you had wheezing or whistling in the chest in the past 12 months?**

☐ Yes

☐ No

**IF YOU HAVE ANSWERED "NO" PLEASE SKIP TO QUESTION 5**

2. **How many attacks of wheezing have you had in the past 12 months?**

☐ None

☐ 1 to 3

☐ 4 to 12

☐ More than 12

3. **In the past 12 months, how often, on average, has your sleep been disturbed due to wheezing?**

☐ Never woken with wheezing

☐ Less than one night per week

☐ One or more nights per week

4. **In the past 12 months, has wheezing ever been severe enough to limit your speech to only one or two words at a time between breaths?**

☐ Yes

☐ No

5. **Have you ever had asthma?**

☐ Yes

☐ No

**IF YOU HAVE ANSWERED YES,**

6. **Was your asthma confirmed by a doctor?**

☐ Yes

☐ No

NIHR | National Institute for Health Research

ACACIA Breathing Survey, v2.0, 29/05/2019

Queen Mary University of London
