# Supplementary material for: Barriers to childhood asthma care in sub-Saharan Africa: a multicountry qualitative study with children and their caregivers
Source: BMJ Open. 2023 Sep 1;13(9):e070784. doi: 10.1136/bmjopen-2022-070784 (PMC10476107; doi:10.1136/bmjopen-2022-070784)
Supplement: Supplementary data [file bmjopen-2022-070784supp002.pdf]

acacia achieving control of asthma in children in africa

FGD 1: Children (12-14 yrs) with severe asthma and diagnosis of asthma

Before the discussion

1. Introduction
2. Game/warming up exercise

Discussion

1. Starter activity: Go around the room, everyone should finish the sentence: 'Asthma is…

   (The facilitators start, e.g. Asthma is… not easy/ hard to talk about/ limiting/ not a problem…)

2. How much do breathing problems affect what you do?
   - … At school?
   - … At home?
   - … Anywhere else?
3. If you have asthma medication, how do you take your asthma medications?
   - What helps you take your asthma medication?
   - What makes it difficult to take your asthma medication?
   - What do you dislike about your asthma medication?
   - Is there any other medication that you think helps you? For example traditional medication?
   - What are some of the ideas you have to make it easier to take your asthma medication?
4. How do you feel if you have difficulties breathing?
   - … When you are around your friends?
     - How do they react?
   - … When you are in class?
     - How does the teacher react?
5. What do you believe people around you think about your asthma?
   - Friends,
   - family,
   - teachers
   - What do you tell people about your asthma? And who do you talk to?

acacia
achieving control of
asthma in children in africa

FGD 2: Children (12-14yrs) with severe asthma and no diagnosis

Before the discussion:

1. Introduction

2. Game/warming up exercise

Discussion:

1. What would you do if you have difficulties breathing?
    - Can you think of anything that might make it easier to breathe?
    - Do you think there is anything that might make it worse?
2. Have you ever taken any medication or remedies for your breathing difficulty?
    - Tell me about it.
3. How much do breathing problems affect what you do?
    - … At school?
    - … At home?
    - … Anywhere else?
4. How do you feel if you have difficulties breathing...?
    - … When you are around your friends?
        - How do you think they would react?
    - … When you are in class?
        - How has the teacher reacted?
5. Have you heard of asthma?
    - What do you think does it mean to have asthma?
6. What would you think if a very good friend suddenly tells you that he/she has asthma?

Naidoo KL, *et al. BMJ Open* 2023; 13:e070784. doi: 10.1136/bmjopen-2022-070784

acacia
achieving control of
asthma in children in africa

FGD 3: Parents of children with diagnosed asthma

Before the discussion

1. Introduction
2. Warm up exercise / tea & cookies
3. Short questionnaire
   - Knowledge questions (symptoms of asthma/triggers etc.)
   - Questions about beliefs around asthma (e.g. How much support do you think a person with asthma needs? A lot,…, none)
   - Where do you go to seek help?

Discussion

1. Asthma is… (finish sentence)
2. Tell us about your experience with asthma?
   - Tell us about your experience with healthcare
     - diagnosis
   - … Experience with asthma treatment (inhalers, alternative treatment methods etc.)
   - Tell us about any impact the asthma diagnosis had for you and your family
     - Financially
     - Socially
3. Can you tell us what makes it difficult for your child to take the asthma medication?
4. What do you think it means for your child to have asthma?
   - Would you have any special concerns about your child because of their asthma?
     - Would you think there is anything that an asthmatic child should avoid?
5. Do you have any examples of what makes life with asthma difficult for your child?
6. Is there anything that helps your child to live with asthma, apart from medication?

FGD 4: Parents of children without asthma

Before the discussion

1. Introduction
2. Warm up exercise/ tea & cookies
3. Short questionnaire
   - Knowledge questions (symptoms of asthma/triggers etc.)

Naidoo KL, *et al. BMJ Open* 2023; 13:e070784. doi: 10.1136/bmjopen-2022-070784

- Questions about beliefs around asthma (e.g. How much support do you think a person with asthma needs? A lot,…, none)
- If your child had problems to breathe sometimes, where would you go to seek help?

Discussion

1. How would you describe asthma?
   - Tell us about your experience with asthma (Do you know anyone with asthma?)
   - If you hear that your child's friend had asthma, what would you say to your child?
2. If you are told that your child has asthma,
   - How would you react?
   - What would you tell your child?
   - Where would you seek help?
   - How would you manage?
   - How would you feel?
3. Why do you think someone might be worried about asthma?
   - Who do you think might be affected by asthma?

Naidoo KL, *et al. BMJ Open* 2023; 13:e070784. doi: 10.1136/bmjopen-2022-070784
