# Supplementary material for: Barriers to childhood asthma care in sub-Saharan Africa: a multicountry qualitative study with children and their caregivers
Source: BMJ Open. 2023 Sep 1;13(9):e070784. doi: 10.1136/bmjopen-2022-070784 (PMC10476107; doi:10.1136/bmjopen-2022-070784)
Supplement: Supplementary data [file bmjopen-2022-070784supp003.pdf]

FGD 1: Children (12-14 yrs.) with severe asthma and diagnosis of asthma		
**Code**	**Definition**	**Purpose/meaning of code**
1. Definition of asthma	Participant defines asthma	Indicates participant's knowledge of what asthma is<br>Indicates Knowledge
2. Effects of breathing difficulties	Participant describes their own experience of how their asthma affects them when they are in various environments such as school, home and anywhere else	Participant indicates how much their breathing problems alter their activities in different environments.
3. Positive experience with asthma medication	participants describe what is pleasing to them about their medications and why they use them	participant describe what improves their symptoms<br>what makes it easy for them to use the medications
4. Negative experience with asthma medication	Participant describe what is not pleasing about their medication and why they do not like to use them.	What make is difficult to take their medication, can be taste, smell, stigma attached to taking medication in public
5. Alternative medication	Participant indicates and describes an experience of taking any alternative medication for their asthma	Indicate alternative medication/methods or idea to relieve their symptoms. Can be at school, home or anywhere else
6. reaction of friends to an asthma attack	How their friends have reacted to them while having an asthma attack and how they felt about their friend's reaction	Determine if there are perceived stigma limiting expression of symptoms. Determine the extent to which peers could provide support or otherwise
7. Reaction to having an asthmatic attack teachers	Participant recalls or describes how their teachers reacted when they were having an asthma attack and how they felt when that was happening	How they perceive getting help from the teachers. Finding out the aid that is offered to the learner and how teachers respond/react to asthmatic learner
8. Perception of asthma from friends	Describe whether friends are aware that they have asthma or not? Describe how the friends feel about having an asthma sufferer as a friend	Describe friends' reaction to them having asthma.<br>how has the relationship being impacted
9. Perception of asthma from family	Participants describe what they family think or feel about their asthma	Are they treated differently in their family because of their asthma? How does their family react when they exhibit symptoms? Have there been any changes to the household since their diagnosis?
10. Perceptions of asthma from teachers	Participants describe what their teachers feel about them having asthma	Do their teachers know they have asthma? What kind of assistance do they receive from the teachers to reduce risk of developing symptoms in school?<br>What kind of assistance do they receive when they exhibit asthma symptoms?
11. Personal perceptions about your asthma	Participants describe how they feel about their own asthma, what are their experiences around it	We are trying to find out how they feel or think about their asthma, what are their experiences around it, good or bad

FGD 2: Children (12-14yrs) with severe asthma and no diagnosis		
**Code**	**Definition**	**Purpose/meaning of code**
1. Easing breathing difficulties	Participants describe what makes it easier for them to breath when they have trouble breathing	What are their coping mechanisms, what do they do in order for their symptoms not to get worse
2. Exacerbations of breathing difficulties	Participants describe what makes it worse for them when they have difficulties breathing	identify things that make their symptom worse
3. Medications	Participants indicate if they have taken any medications for their symptoms or any alternative methods to help them with their symptoms. Identify medication use : type	Finding out what medications and remedies they have used. Describe what is done to bring relief
4. Effects of experiencing breathing problem	Participant describes their experience of how their asthma affects them when they are in various environments such as school, home and anywhere else	description of experience of asthma symptoms- home, school etc.
5. Reaction from friends	Participant recalls and describe how their friends reacted when they were having an asthma attack and how they felt when that was happening	How they perceive getting help from their peers. Finding out they experience any stigma from exhibiting breathing difficulties in front of their friends
6. Reaction from teachers	Participant recalls and describe how their teachers reacted when they were having an asthma attack and how they felt when that was happening	How they perceive the help or otherwise from school. describe what was done and the teacher's reaction
7. Perceptions of having breathing problems /asthma	Participant perception of what asthma is and how they will react if a friend have asthma	Definition of asthma and perception of it

FGD 3: Parents of children with diagnosed asthma		
**Code**	**Definition**	**Purpose/meaning of code**
1.Definition of asthma	Participant defines asthma/ describe asthma symptoms	Indicates participant's knowledge of what asthma is
2. Response to initial asthma diagnosis	Participant describes their experience when their child was diagnosed with asthma	Describe parents' initial reaction to asthma diagnosis
3.Experience with healthcare with regards asthma		Journey to diagnosis of asthma in healthcare
4.Positive experiences asthma medication	Participants describes their experience with asthma treatment	describe parents knowledge about asthma treatment and support provided when child was diagnosed
5.Negative experience with asthma medication	Participant describes negative aspects of asthma medication. What they don't like about the medication	What make is difficult to for their children to take the medication, can be taste, smell, stigma attached to taking medication in public
6.Financial impact of asthma diagnosis	Cost of medication, availability of medication at their local healthcare facility, fear of side effects	determine the financial implication (direct and indirect cost of asthma care)
7.Social implications of the diagnosis	Social implications of the asthma diagnosis on their children and family	Try to find out the perceptions and attitude to asthma in the community they live in or perceptions from their family. Find out the if they have experienced stigma or support
8.Concerns around asthma	What it means for their child to have asthma. Their perceptions and worries around the diagnosis and care of asthma	How people would Perceive or treat their child, how the child would cope if not at home etc.
9.Enablers in asthma care of children	Participant describes what makes it easier for their child with an asthma diagnosis to be cared for	Parents perception of what will make life easier for a child with asthma
10. Difficulties of an asthma diagnosis	Participant describes what has been difficult since the asthma diagnosis	Parents perception of what has made life difficult for a child with asthma

**FGD 4: Parents of children without asthma**		
**Codes**	**Definition**	**Purpose/meaning code**
1. Knowledge of breathing problems in children	Participant recalls and describes their experience and knowledge around asthma	Try to find out their knowledge around asthma and if they know someone with asthma
2. Perception of asthma in other children	Their reactions if their child's friend had asthma	Try to find out their perception around asthma, fear, stigma etc.
3. Reaction if their child had asthma	Their reaction if their child was diagnosed with asthma	Try to find out their perception and feeling about asthma
4. communicating with child on asthma	How would they tell their child about the diagnosis	Try to find out their knowledge around asthma or what resources they would use to tell the child more about asthma. How would they communicate asthma information to their child
5. Seeking help if their child had asthma	Where would they go to seek help for asthma, asthma care, treatment and information	Try to find out if they have access to resources that can help them and their child. What type kind of resources are available to them in case of emergencies<br>Where they would seek help i.e. from people, places
6. Manage asthma care/Breathing problems	Participant describe what they would do differently if their child was diagnosed with asthma and how they would care for them	How would they manage the care of their child who has asthma- things they would do in terms of care
7. Positive Feelings on asthma	Their perceptions around asthma	any positive feeling described
8. Negative feelings /Concerns around asthma	Participant describes their concerns about asthma or an asthmatic's concern about asthma	Describes such feeling as fear, afraid of stigma, afraid of financial burden, burden of visiting hospital frequently, fear for sudden asthma attacks or what the child may not be able to do.
