# Supplementary material for: Barriers to childhood asthma care in sub-Saharan Africa: a multicountry qualitative study with children and their caregivers
Source: BMJ Open. 2023 Sep 1;13(9):e070784. doi: 10.1136/bmjopen-2022-070784 (PMC10476107; doi:10.1136/bmjopen-2022-070784)
Supplement: Author's manuscript [file bmjopen-2022-070784.draft_revisions.pdf]

BMJ Open

BMJ Open is committed to open peer review. As part of this commitment we make the peer review history of every article we publish publicly available.

When an article is published we post the peer reviewers' comments and the authors' responses online. We also post the versions of the paper that were used during peer review. These are the versions that the peer review comments apply to.

The versions of the paper that follow are the versions that were submitted during the peer review process. They are not the versions of record or the final published versions. They should not be cited or distributed as the published version of this manuscript.

BMJ Open is an open access journal and the full, final, typeset and author-corrected version of record of the manuscript is available on our site with no access controls, subscription charges or pay-per-view fees (http://bmjopen.bmj.com).

If you have any questions on BMJ Open's open peer review process please email info.bmjopen@bmj.com

BMJ Open

Barriers to Childhood asthma care in Sub-Saharan Africa: a multi-country qualitative study with children and their caregivers.

Journal:	*BMJ Open*
Manuscript ID	bmjopen-2022-070784
Article Type:	Original research
Date Submitted by the Author:	04-Dec-2022
Complete List of Authors:	Naidoo, Kimesh; King Edward VIII Hospital, Nelson R Mandela School of Medicine University of KwaZulu-Natal, Paediatrics and Child health<br>Dladla, Sindiswa; College of Health Sciences University of KwaZulu-Natal, Department of Paediatrics and Child Health<br>Mphahlele, Reratilwe; University of KwaZulu-Natal Nelson R Mandela School of Medicine, Paediatrics and Child Health<br>Mosler, Gioia; Queen Mary University of London, Centre for Genomics and Child Health<br>Muyemayema, S; University of Zimbabwe, Department of Primary Health Care Sciences<br>Ssemata, Andrew; Makerere University College of Health Sciences, Psychiatry<br>Mkutumula, E; College of Medicine, 6. Malawi Liverpool Wellcome Programme, Queen Elizabeth Central Hospital, College of Medicine, Chichiri, Malawi<br>Adeyeye, Olayinka; Lagos State University College of Medicine<br>Moyo, Melinda ; University of Zimbabwe, 3. Child and Adolescent Health Unit (CAHU), Department of Primary Health Care Sciences<br>Goodman, Y; Lagos State University College of Medicine Ikeja; Lagos State University Teaching Hospital<br>Kuyinu, Y; Lagos State University College of Medicine Ikeja; Lagos State University Teaching Hospital<br>Nantanda, Rebecca; Makerere University, Lung Institute, Makerere College of Health Sciences<br>Addo-Yobo, Emmanuel; Kwame Nkrumah University of Science and Technology, School of Medicine and Dentistry, College of Health Sciences<br>Ticklay, Ismail; University of Zimbabwe College of Health Sciences, Paediatrics and Child Health; University of Zimbabwe College of Health Sciences, Medical School Parirenyatwa Hospital<br>HA, Mujuru; University of Zimbabwe, Department of Primary Health Care Sciences<br>Grigg, Jonathan; Queen Mary University London, Paediatric Respiratory<br>Masekela, R ; University of KwaZulu-Natal College of Health Sciences, Paediatrics and Child Health
Keywords:	Asthma < THORACIC MEDICINE, PAEDIATRICS, RESPIRATORY MEDICINE (see Thoracic Medicine), PUBLIC HEALTH

SCHOLARONE™
Manuscripts

I, the Submitting Author has the right to grant and does grant on behalf of all authors of the Work (as defined in the below author licence), an exclusive licence and/or a non-exclusive licence for contributions from authors who are: i) UK Crown employees; ii) where BMJ has agreed a CC-BY licence shall apply, and/or iii) in accordance with the terms applicable for US Federal Government officers or employees acting as part of their official duties; on a worldwide, perpetual, irrevocable, royalty-free basis to BMJ Publishing Group Ltd ("BMJ") its licensees and where the relevant Journal is co-owned by BMJ to the co-owners of the Journal, to publish the Work in this journal and any other BMJ products and to exploit all rights, as set out in our licence.

The Submitting Author accepts and understands that any supply made under these terms is made by BMJ to the Submitting Author unless you are acting as an employee on behalf of your employer or a postgraduate student of an affiliated institution which is paying any applicable article publishing charge ("APC") for Open Access articles. Where the Submitting Author wishes to make the Work available on an Open Access basis (and intends to pay the relevant APC), the terms of reuse of such Open Access shall be governed by a Creative Commons licence – details of these licences and which Creative Commons licence will apply to this Work are set out in our licence referred to above.

Other than as permitted in any relevant BMJ Author's Self Archiving Policies, I confirm this Work has not been accepted for publication elsewhere, is not being considered for publication elsewhere and does not duplicate material already published. I confirm all authors consent to publication of this Work and authorise the granting of this licence.

For peer review only

Title: Barriers to Childhood asthma care in Sub-Saharan Africa: a multi-country qualitative study with children and their caregivers.

Authors:

Naidoo KL, PhD[1]; Dladla S[1]; Mphahlele REM[1]; Mosler G[2], PhD; Muyemayema S[3]; Ssemata AS[4 5]; Mkutumula E[6]; Adeyeye O[7 8]; Moyo M[3]; Goodman Y[7 8]; Kuyinu Y[7 8]; Nantanda R[9 10]; Addo-Yobo E[11]; Ticklay I [3 12]; Mujuru HA[3]; Grigg J[2*], PhD; Masekela R[1], PhD*

1. Department of Paediatrics and Child Health, School of Clinical Medicine, College of Health Sciences, University of KwaZulu-Natal
2. Centre for Genomics and Child Health, Blizard Institute, Barts and The London School of Medicine and Dentistry, Queen Mary University of London, London, UK
3. Child and Adolescent Health Unit (CAHU), Department of Primary Health Care Sciences, Faculty of Medicine and Health Sciences, University of Zimbabwe, Avondale, Harare, Zimbabwe
4. Department of Psychiatry, College of Health Sciences, Makerere University, Kampala, Uganda
5. Department of Global Health and Development, London School of Hygiene and Tropical Medicine, London, UK
6. Malawi Liverpool Wellcome Programme, Queen Elizabeth Central Hospital, College of Medicine, Chichiri, Malawi
7. Lagos State University College of Medicine Ikeja, Lagos, Nigeria
8. Lagos State University Teaching Hospital, Ikeja, Lagos, Nigeria
9. Makerere University Lung Institute, Makerere University College of Health Sciences, Kampala, Uganda
10. Department of Paediatrics and Child Health, Makerere University College of Health Sciences, Kampala, Uganda
11. Department of Child Health, Komfo Anokye Teaching Hospital, Kumasi, Ghana
12. Parirenyatwa Group of Hospitals, Causeway, Harare, Zimbabwe

For peer review only - http://bmjopen.bmj.com/site/about/guidelines.xhtml

ABSTRACT – word count = 293

Objectives

This study identifies barriers and provides recommendations to improve asthma care in children across sub-Saharan Africa, where qualitative data is lacking despite high rates.

Design

One of the aims of our National Institute for Health Research global health research group “Achieving Control of Asthma in Children in Africa” (ACACIA) was to use qualitative thematic analysis of transcribed audio recordings from focus group discussions (FGDs) to describe barriers to achieving good asthma control.

Setting

Schools in Blantyre (Malawi), Lagos (Nigeria), Durban (South Africa), Kampala (Uganda), and Harare (Zimbabwe).

Participants

Children (n=136), 12 to 14 years with either asthma symptoms or a diagnosis and their caregivers participated in 39 FGDs. All were recruited using asthma control questions from the Global Asthma Network survey.

Results

There were four key themes related to asthma identified: (1) reacting to the diagnosis, (2) managing symptoms, (3) accessing health care and (4) use of medications across all countries. An inadequate understanding of environmental triggers, a hesitancy in using metered dose inhalers and a preference for oral and alternate medications were identified as barriers. In addition, limited access to health care with delays in diagnosis and an inability to cope with expected lifestyle changes was reported. Based on these findings, we recommend tailored education to promote access to and acceptance of metered dose inhalers, including advocating for access to a single therapeutic, preventative and treatment option. Furthermore, healthcare systems should have simpler diagnostic pathways and easier emergency access for asthma.

Conclusions

In a continent with rapidly increasing levels of poorly controlled asthma, we identified multiple barriers to achieving good asthma control along the entire trajectory of care. Exploration of these barriers reveals several broad, generalisable recommendations that should modify asthma care plans and potentially transform asthma care in Africa.

Trial registration number 269211.

For peer review only

Key messages statements /strengths and limitations

- One of the first cross-Africa studies to analyse perspectives of children with both doctor-diagnosed asthma and severe wheezing symptoms without a diagnosis;
- A large and rich dataset identifies common barriers related to personal preferences and universal challenges to accessing optimal asthma care; and
- Several generalisable recommendations that include evidence-based advocacy for universal access to using combination inhalers across Africa are identified.

Keywords: children , asthma, Childhood asthma, sub-Saharan Africa,

Funding statement

This research was funded by the National Institute for Health Research (NIHR) (Trial registration number 269211) using UK aid from the UK Government to support global health research.

Disclaimer The views expressed in this publication are those of the author(s) and not necessarily those of the NIHR or the UK Department of Health and Social Care (17/63/38).

Main manuscript word count = 4123 (excluding Figures and Tables)

INTRODUCTION

Globally, asthma is the most common non-communicable respiratory disease among children.[1] While the prevalence of asthma in African children is currently 14% and matches the global average, it is increasing in low-middle income countries (LMIC) with different trajectories suggested by the wide range of prevalence between countries, for example, Kenya at 15.8%, Nigeria 13.0%, and South Africa 20.3%. [1-4] The goal of asthma care is to optimise symptom control and maintain a good quality of life.[5, 6] Asthma control has been reported to be poor with the 2018 Global Asthma Report reporting high uncontrolled asthma prevalence rates across sub-Saharan Africa (sSA).[4] The determinants of poor asthma control in sSA are multifactorial including environmental factors which are certainly important and include rural to urban migration, early life urban residence, overcrowding, poorer air quality, lifestyle changes and diet.[6-9] Patient-related factors reported in African children include poor knowledge and

For peer review only - http://bmjopen.bmj.com/site/about/guidelines.xhtml

symptom recognition, non-acceptance of inhaler treatment, older age and concurrent allergic rhinitis.[6,7] Health system factors include limited diagnosis of asthma, episodic management of asthma symptoms at the primary health care (PHC) level, and poor access to inhaled corticosteroids.[5,6,9-12]. However, to date, there is little qualitative data from children and young people on the determinants of asthma control in this region. Studies in high-income countries have found that children's and caregivers' health beliefs about asthma and poor relationships with healthcare providers (HCPs) can undermine asthma management.[13] Caregivers have also reported fear, feeling of powerlessness, uncertainty, and social tension related to their children who have asthma.[14-16] Studies from individual African countries have also identified physical, emotional and social burdens related to childhood asthma, and these have been postulated to affect non-adherence to recommended treatment regimens.[10,17-19]

Understanding the views of caregivers and affected children with doctor-diagnosed asthma and those with symptoms suggestive of asthma are needed to capture perspectives effectively. To accurately and efficiently determine strategies to improve asthma care, child-generated strategies from affected African children and their caregiver's concerns and recommendations need to be identified and explored.[20] A better understanding of identified barriers to asthma care in African countries will assist with advocacy and policy intervention to improve the supply and financing of asthma medicines across the continent and aid the development of strategies to improve retention in care.[21] This study aimed to capture the views of asthmatic children with and their caregivers, in sSA ,to identify barriers to asthma diagnosis and care as well as interventions to improve asthma care across countries in Africa.

METHODS

Study design

This study was a component of the achieving control of the National Institute for Health Research (NIHR)funded global health research group's "Achieving control of asthma in children in Africa" (ACACIA), a larger observational cohort study across six African countries; Ghana, Malawi, Nigeria, South Africa, Uganda and Zimbabwe.[22] The ACACIA project aims to determine the burden of asthma in school-going children by assessing asthma control in children between 12 and 14 years with symptoms compatible with asthma or a doctor-diagnosis of asthma to assess symptom severity and asthma control. As a separate study, we conducted focus group discussions (FGDs) with children with an asthma diagnosis or symptoms suggestive of asthma and their caregivers to obtain their perspectives on the barriers

For peer review only - http://bmjopen.bmj.com/site/about/guidelines.xhtml

to managing asthma. The study plan, procedures and methods were standardised across all countries.[22]

Study population

Children and their caregivers were recruited initially as part of the ACACIA dataset. A Breathing Survey developed from questions in the Global Asthma Network (GAN) questionnaire (Supplementary file 1)was used to identify participants between 12 and 14 years with asthma symptoms or an asthma diagnosis.[22] Focus group participants (both children and their caregivers) were recruited using purposive sampling and categorised into four groups; children with a doctor-diagnosis of asthma (CAD) and their parents/caregivers (PAD), children with severe wheezing symptoms without a doctor-diagnosis of asthma (CND) and their parents/caregivers (PND), with FGDs held separately for each category. The aim of this group categorisation was to ensure views of both children and caregivers, as well as those with and without a formal asthma diagnosis, were adequately sampled.

Study setting

The FGDs were held between 1 November 2020 and 30 June 2021 in schools in cities across countries: (Blantyre), Malawi; (Lagos), Nigeria; (Durban), South Africa; (Kampala), Uganda; and (Harare), Zimbabwe. Data from Ghana were not included for analysis due to challenges with COVID-19 restrictions in that country that impacted data collection. The in-country team of researchers and fieldworkers facilitated the focus group discussions.

Data collection

Focus groups were conducted face-to-face, with adherence to in-country-specific COVID-19 regulations. All FGDs were moderated by at least two facilitators, one of whom was a study investigator at each site. FGDs took place in English and /or isiZulu (SA), Shona (Zimbabwe) and Chichewa (Malawi). Focus groups were audio-recorded, transcribed verbatim and translated to English if needed in the country. Discussions lasted between 40-60 minutes and followed a predefined question guide specific to each focus group category. The discussion guides were developed after multiple iterations by the ACACIA core study team and informed by the study aims and literature review on barriers to asthma care. (Supplementary file 2)

Data analysis

For peer review only

The transcripts taken during the discussion were used to analyse the data using the NVivo 1.6 software program. We used an inductive analysis to examine the data.[23] Ten members of the ACACIA research group from all the participating countries independently developed codes from the transcripts and then discussed the coding to develop a code book.[23,24] At least two investigators reviewed each transcript. A reiterative process was followed to apply the codebook to the transcribed data and determine whether further codes emerged. When no new codes emerged, the codebook was assumed to be a valid representation of the data and finalised (Supplementary 3).

Braun and Clarke's six-step thematic analysis was then used as the analytic framework to identify the patterns and themes within the coded data.[25] Major themes and subthemes were identified based on patterns and relationships noted between derived codes, and all investigators agreed upon these after multiple iterations. The themes that were common across countries were specifically identified and highlighted and where specific in-country views differed these were noted.

ETHICS

Ethical approval was granted at all the study sites: Malawi, COMREC Reference Number: P.10/18/2494; Nigeria, LREC 06/10/1084; Uganda, MHREC 1514, and UNCST SS 4940; Zimbabwe, MRCZ/A/2415; South Africa, BREC Ref No.: BF002/19. Participants provided written informed consent as well as informed caregiver (parent /guardian) consent and child assent in accordance with both country/local regulations and United Kingdom (UK) ethics regulations. Trial registration number 269211. Data were de-identified and stored on a password-protected computer with access only to researchers.

RESULTS

Description of the focus groups conducted

The data from 39 transcribed FGDs reflecting 256 participants across five countries were analysed. Table 1 indicates the breakdown of these groups per category and country. A total of 136 children were included with CAD 45.6% (n=62) and CND 54.4% (n=74). Of the 120 caregivers included they included PAD 42.5% (n=51) and PND 57.5% (n=69)

Table 1: Summary of children with asthma or asthma symptoms and their caregivers who participated in focus group discussions (n=256).

For peer review only

For peer review only - http://bmjopen.bmj.com/site/about/guidelines.xhtml

Summary of findings

Table 2 illustrates the inter-relationship of the subthemes and themes identified. There were four major themes identified which were grouped into: (1) understanding asthma, (2) reactions to the diagnosis, (3) managing symptoms and access to health care and (4) asthma and medications with sub-themes in each category.

Table 2. Major themes and subthemes and their relationships

Understanding Asthma

This theme related to perspectives on how caregivers and children defined asthma and what was considered the cause of asthma. Across all groups, asthma was seen as "a *set of conditions that is associated with breathing and affecting the lungs"* (PAD, NG Group 1). We could not ascertain differences in knowledge amongst caregivers of those with and without a diagnosis of asthma.

> *It's prolonged congestion which comes in allergy form. In most cases those children with asthma have breathing problems like their cough doesn't break very fast. It is dry and, in that dryness, that's when you hear the wheezing (*PAD, UG Group 1)

We did find a distinction in how caregivers and children understood the causation of asthma. Children expressed fewer opinions on the familial or genetic nature of asthma than their caregivers. In all groups, there was a general ambivalence about expressing clear roles of how environmental triggers and other factors such as prematurity and genetic predisposition contribute to the development of asthma.

> *There is dirt of some kind that interrupts ones breathing in their lungs so when air is meant to come in then you'll find that it's this dirt that is blocking the inhalation and exhalation of air.* (PND ZA, Group 6)

> *The father of my mother had that complication* (reference to asthma) *and now this is contradictory because now it's my girl that has that complication but the three boys are okay*. (PAD, UG, Group 2)

Reacting to asthma diagnosis

This theme related to perspectives being expressed around the time of being diagnosed and was reflected predominantly in the groups with a formal doctor-diagnosis. The sub-themes

For peer review only - http://bmjopen.bmj.com/site/about/guidelines.xhtml

identified include concerns with delays in diagnosis, the emotional distress felt with disruptions to lifestyles and reactions of others to the diagnosis.

These perspectives included specific concerns about the perceived reluctance of health care workers to making an asthma diagnosis and the resulting frustration these delays cause . There were no references to the use of peak flow meters in the diagnostic workup of these children.

> *I think that symptoms start with cough and flu. We waste a lot of time and yet the actual disease is not treated. So, after you have given that dose of cough and flu, when you go back, the doctor will tell you that is an infection yet it is asthma* (PAD, UG, Group2)

> *It started when he was still young and when we went to the doctor, I think he was in kindergarten, they said we must change the milk…and then they said maybe he was too fat. The chest was too tight. And then we stopped the milk and then he didn't get any better* (PAD, ZA, Group2)

Both children and caregivers indicated displeasure about the disruption in activities of normal daily life after being diagnosed with asthma. These included disruptions to school attendance, participation in sports and the child's share of household chores. Children expressed emotions of sadness and loss whilst caregivers expressed anxiety and reflected on the practical aspects that related to the disruption of their previous activities.

> *When am in an attack I can't do anything, I can't study, I can't go to class, I can't breathe properly, I can't focus, I can't understand. I can't be at school.* (CAD, MW, Group1)

> *Since we discovered she has asthma, my daughter is not exposed to a lot of things anymore. We don't allow her to sweep except if she's covered with a face mask before sweeping, we stopped her from drinking cold water and her room is cleaned properly, no rug, no cockroaches in the house, the fan is kept clean.* (PAD, NG, Group2)

Children also specifically expressed concerns about the stigma and discrimination they endured when their asthma diagnosis was known in the school environment. Fears of being isolated led to non-disclosure.

For peer review only - http://bmjopen.bmj.com/site/about/guidelines.xhtml

It's also embarrassing because they all look at you funny and some start excluding you from games because they fear that you will have an attack. Some end up avoiding you forever and you remain with no one to play with. (CAD, ZW, Group1)

I don't tell people that I am asthmatic because they will start looking at me all different. (CAD, UG, Group2)

Managing symptoms and access to healthcare

This theme related to views on the participants of the non-pharmacological intervention they undertook in relation to addressing the symptoms associated with asthma.

In all groups, identifying and avoiding various asthma triggers was seen as an important component in managing symptoms . For caregivers, these avoidance strategies translated to altering the external environments of their children, whilst children focused more on how lifestyle changes impacted their daily lives. All groups articulated that they had difficulties with understanding what the various environmental triggers are and how to avoid them effectively.

Simple solutions like make sure your surroundings are clean, check for dampness, in your bathroom or the ceilings and your walls … there are lots of things you can do to ensure that, the triggers are reduced. (PND, ZA, Group2)

I was outside and then my sister started sweeping the compound raising dust, the dust affected me and I started wheezing. I was then told to go inside the house and sleep. (CAD, ZW, Group1)

The theme also includes caregivers' perspectives on their interactions with various levels of the health systems, particularly during acute asthma episodes. In these interactions, caregivers reflected on their frustrations, financial dependencies and vulnerabilities. These arose following the experiences of limited and often difficult access to health care during an acute asthmatic attack.

For peer review only

For peer review only - http://bmjopen.bmj.com/site/about/guidelines.xhtml

> *Government hospitals are useless … It is possible that the doctor's house is very near but for him to come is a challenge. Sometimes they will leave in the middle of their shift and you come with your child to the hospital and they tell you to wait … meanwhile your child's condition is worsening* (PAD, MW, Group 1)

> *Apart from the hospital, I would also seek help from more accessible nurses in the neighbourhood or at home because these attacks usually happen when the hospital is too far and, in that moment, they may not be able to help you.* (PND, UG, Group1)

Asthma and medications

Across all groups, common perspectives on the role, availability and acceptance of various types of medications to treat asthma were identified.

All groups reflected on relatively easier access to and acceptance of alternate and complementary asthma treatments than prescribed asthma medications.

> *Others told me to find a chameleon and leave it on flour for it to walk over, then to use the flour to make porridge to give the child. None of those things really worked! I have taken too much of these traditional medications!* (PAD, MW, Group1)

> *I also use the inhaling smoke method. My mother does the same thing of putting marijuana seeds on hot coal and covers me with a blanket to inhale the smoke but it will be for five minutes tops and it clears my chest and I breathe better.* (CND, ZW, Group1)

Juxtaposed to the acceptance of various alternate medications and treatment, there are widely held views that using and relying on inhalers should be met with caution. In addition, caregivers and children with an asthma and no asthma diagnosis expressed various fears and myths about using prescribed inhalers. Concerns over possible addiction developing with the use of inhalers and fears of what could happen when inhalers are unavailable were expressed. In addition to these concerns, there is a clearly articulated preference for oral over inhaled medication by many. It is important to note that some caregivers and children with asthma (largely in groups with a formal diagnosis) did note the improvements in asthma symptoms with the regular use of prescribed inhalers.

For peer review only - http://bmjopen.bmj.com/site/about/guidelines.xhtml

*Okay a lot of people would tell us …we can use the inhalers but when I grow up it will build up in my body … also what if I get addicted to the inhaler and one day I have shortness of breath and don't have the inhaler? What will happen? (*CAD, MW, group2)

*The people from my village saw that I am using an inhaler, they asked me why I have started using it as if I don't know how such things end. But since last year when I started using the inhaler, everything is fine and we have not been admitted in the hospital since. I really had that mind-set that inhalers kill but since I started using them I saw that they are really helpful (*PAD, MW, and group1)

Barriers identified across countries

From the themes, we identified several barriers to asthma care across the five African countries, Table 3. Although there was some understanding of the role of genetic predisposition and allergen triggers, children and caregivers still expressed the need for more information, specifically regarding trigger avoidance. This included lifestyle changes that had to be made for families and disruption of normal family routines and chores. Reliance on the use of oral and complementary medications was expressed with the stigma around the use of asthma inhaler therapies.

Table 3: Identified Barriers to asthma care across African countries

Discussion

Main findings

Overall, we identified several barriers to asthma care that were common in all five African countries in this study. The significance of these common barriers to care in Africa is highlighted, and on further exploration, several cross-cutting recommendations are summarised in Table 4.

Table 4 Recommendations to improve asthma care across Africa

For peer review only - http://bmjopen.bmj.com/site/about/guidelines.xhtml

Inadequate understanding of environmental triggers and genetic predisposition in causing asthma

Asthma is a complex heterogeneous condition with multiple risk factors. The role of genetics, atopy and environment is complex even for experienced researchers. It is therefore not surprising that there was a poor societal understanding of the differences between cure, prevention and control, including confusion between trigger avoidance and symptom control using chronic treatments. Studies from sSA have noted that caregivers and children ascribe triggers with causation[10,17]. Thus, removing these 'offenders' equates to treatment/cure, lessening the perceived necessity for measures to prevent or control asthma.[10,17]. These perspectives are identified in our study's findings of an inadequate understanding of environmental triggers, such as dust and cold weather. We speculate that this inadequate understanding translates to low 'necessity beliefs' towards the administration of chronic medications documented in Africa.[17] We thus further suggest that asthma education programs should clarify the role of triggers in the precipitation of asthma episodes and the need for medications for asthma control.

Hesitancy in health care workers making an asthma diagnosis resulting in delays and frustration

The delays in children receiving a doctor-diagnosis of asthma identified in our study can possibly be viewed within the context of poor symptom recognition, episodic management and low level of implementation of existing asthma guidelines by health care workers especially noted at the primary health care level (PHC) in Africa.[17,26] A systematic review by Mphahlele RE et al. records low rates of asthma diagnosis (38.8%), prescribed medication (47.6%) and an increasing prevalence of severe uncontrolled asthma in African children.[6] Our study further identifies that there is little knowledge of peak flow meters which could provide a cost-effective and simple way to monitor symptom severity. The uptake and usage of peak flow meters for asthma, akin to glucometers for diabetes mellitus previously mooted, warrants assessment.[26] Simplifying diagnostic algorithms for asthma to ensure ease of implementation of care in the African context should be thus viewed as a crucial need. We strongly advocate for this, based on our findings.

Difficulties experienced coping with adjustments in lifestyles

Children's perspectives on being self-conscious when using inhalers and thus appearing 'different' was an important theme in the present study and has been reported across both

For peer review only - http://bmjopen.bmj.com/site/about/guidelines.xhtml

resource-developed and challenged countries.[13,19,26] The stigma associated with asthma and the subsequent reluctance to use inhalers in front of peers thus impacts self-management interventions deemed crucial to most asthma care programmes.[19] The use of child-generated strategies where peer interactions help break down social barriers has been suggested for asthma school education programmes.[27] It is relevant to African policy since asthma education programmes must compete with multiple health care messaging from common infectious diseases like human immunodeficiency virus, tuberculosis and malaria.[20] In this study, we identify peer acceptance as a major barrier in coping with asthma adjustments. We thus recommend that school-going children be sensitised to provide support, specifically with inhaler usage, to affected peers.

Another barrier identified was the difficulty delineating asthma triggers and the inability to alter their environment adequately. Studies from both sSA and elsewhere corroborate this finding, and the use of global checklists that can provide guidance on the range of triggers and viable environment modifications may be useful for inclusion, for example, in the World Health Organization Package of essential non-communicable disease interventions for primary health care (WHO PEN).[28]

Difficult and limited access to health care during an acute asthmatic episode

Poor access to asthma care across resource-constrained contexts, including Africa, has been noted as a general barrier, and our study corroborates this across all sampled countries.[7] This limited and often difficult access often leads to caregivers seeking alternate avenues for care and delaying appropriate care.[29,30] The WHO PEN attempts to improve asthma management at PHC sites; however, logistic challenges, including lack of essential medications at some sites, require interventions.[28] We specifically identify the need for access to oxygen, nebulisers and monitoring, especially during acute episodes. The concern is that while PHC services may be accessible geographically, the cost of transport, waiting times, and loss of income also contribute to poor access. We thus advocate for improved access to both acute and chronic asthma care in primary health care settings.

Easier access to and acceptance of alternate and complementary asthma treatments compared with prescribed asthma medication

The acceptance and usage of traditional medicines and alternative treatments for asthma identified in this study have been previously ascribed to negative perceptions of prescribed medications and seen as inappropriate health-seeking behaviour that contributes to severe

For peer review only - http://bmjopen.bmj.com/site/about/guidelines.xhtml

asthma.[17,26,30] In the current study, although ambivalence to prescribed medications contributes to poor inhaler use, widespread access to traditional and complementary medicine and indigenous knowledge systems also contributed to alternative treatments. We propose that the propagation of key programmes that advocate for and educate on inhaler therapies and devices should actively engage traditional health systems in the African context to understand better and embed asthma education in these systems.

Preference for prescribed oral asthma medication with fears and myths over using metered dose inhalers

Multiple studies have corroborated the poor usage and non-adherence to metered dose inhalers in childhood asthma as a major barrier to care.[11,17] This is further seen with the low levels of inhaled corticosteroids (6.7- 17%) and 73% of doctor-diagnosed/suspected asthma not using any inhaler documented.[6,31] Over-reliance on short-acting beta agonist (SABA) inhalers per se is also a barrier to appropriate care.[32] A preference for oral over inhaler-administered medications and widely held concerns that metered dose inhalers were addictive and harmful identified have been noted previously.[10,18] These factors are identified as leading reasons for inadequate use of metered dose inhalers.

With efficacy and safety studies advocating the use of single rapid onset (long-acting beta-agonist/inhaled corticosteroids (LABA/ICS) metered dose inhalers for maintenance, and reliever therapy there is a potential that this single therapeutic option could transform asthma care in sSA.[5,11] Advocacy and policy interventions to make this therapeutic option cheaper need urgent action.[17,11] We suggest that universal access to using a single therapeutic metered dose inhaler, where appropriate, be strongly advocated for in Africa and, in addition, knowledge to dispel fears of using inhaler therapies and devices be included in asthma care plans.

Strengths and limitations of this study

This study presents a large qualitative dataset from multiple sites across Africa, reflecting a wide participant base. The juxtaposition of children with and without a formal diagnosis and their caregivers generated rich data and new insights. We actively sought to identify common barriers across all sites, thus generating data generalisable to the SSA context. Transcription verbatim and using qualitative software to code the data enhanced the reliability. We attempted to ensure a fair and equitable coding team to decrease in-country biases, and this study thus reflects one of the few unique cross-country analyses from Africa.

For peer review only - http://bmjopen.bmj.com/site/about/guidelines.xhtml

Some loss of meaning may have occurred during the translation of African language quotes however, the data were analysed in the source language by members of the large international coding team who checked the semantic and context equivalence for translated quotes. We did not conduct member checks. We used an inductive approach with constant data comparisons and discussions to inform our understanding of the views expressed. We were aware of the risk of researcher bias in qualitative research and actively discussed reflexivity in the regular multidisciplinary team discussions, which aided analysis and informed interpretation.

Conclusion

In a continent with rapidly increasing levels of severe uncontrolled asthma, multiple barriers to asthma diagnosis and management are identified along the entire trajectory of care. Exploration of these barriers reveals several broad, generalisable recommendations to modify asthma care plans that can mitigate challenges and potentially transform asthma care in Africa. Advocacy and tailored education are needed to promote access and acceptance for metered dose inhalers, including advocating for access to a single therapeutic, preventative and treatment modality. Healthcare systems require simpler diagnostic pathways and easier emergency access for childhood asthma across the continent.

DECLARATIONS

Acknowledgements

The authors wish to express our gratitude to all the children and caregivers who participated in this study.

Funding

This research was funded by the National Institute for Health Research (NIHR) , project number 17/63/38269211, trial registration number: 269211

Availability of data and materials

Anonymised data is available from the corresponding author upon request.

Authors' contributions

RM, JG, HAM, GM, and IT contributed to the study's conception. KLN, REM, SN, AS, MM, YG, YK, RN and OA performed the data collection, checking and coding the transcripts. KLN, REM, SN, AS, MM, YG, YK, RN and OA contributed to data analysis and interpretation. KLN, REM, SN and RM drafted the manuscript, and all authors provided critical revisions and

For peer review only

editing. All authors reviewed the manuscript. RM and JG accept full responsibility for the work and the conduct of the study, had access to the data, and controlled the decision to publish

Disclaimer

The views and opinions expressed in this article are those of the authors and do not necessarily reflect the official policy or position of any affiliated agency of the authors.

Ethical consideration and consent to participate

This is contained in the manuscript.

Consent for publication

Not applicable.

Competing interests

The author(s) declare that they have no financial or personal relationship(s) that may have inappropriately influenced them in writing this article.

References

1. Global Asthma Network. The Global Asthma Report 2018. http://globalasthmanetwork.org/Global%20Asthma%20Report%202018.pdf (accessed 03 October 2022)
2. Mortimer K, Reddel HK, Pitrez PM, et al. Asthma management in low and middle income countries: case for change. *Eur Respir J.* 2022;60(3):2103179. doi: 10.1183/13993003.03179-2021.
3. Adeloye D, Chan KY, Rudan I, Campbell H. An estimate of asthma prevalence in Africa: a systematic analysis. *Croat Med J.* 2013;54(6):519-31. doi: 10.3325/cmj.2013.54.519.
4. Asher MI, Rutter CE, Bissell K, et al.; Global Asthma Network Phase I Study Group. Worldwide trends in the burden of asthma symptoms in school-aged children: Global Asthma Network Phase I cross-sectional study. *Lancet* 2021;398(10311):1569-1580. doi: 10.1016/S0140-6736(21)01450-1.
5. Global Initiative for Asthma. Global Strategy for Asthma Management and Prevention, 2022: GINA 2022. https://ginasthma.org/wp-content/uploads/2022/07/GINA-Main-Report-2022-FINAL-22-07-01-WMS.pdf (accessed 03 October 2022)
6. Mphahlele RE, Kitchin O, Masekela R. Barriers and determinants of asthma control in children and children in Africa: a systematic review. *BMJ Open* 2021;11:e053100. doi: 10.1136/bmjopen-2021-053100.

For peer review only - http://bmjopen.bmj.com/site/about/guidelines.xhtml

7. Meghji J, Mortimer K, Agusti A, et al. Improving lung health in low-income and middle-income countries: from challenges to solutions. *Lancet* 2021 Mar 6;397(10277):928-940. doi: 10.1016/S0140-6736(21)00458-X.
8. Nagel G, Weinmayr G, Kleiner A, et al.; ISAAC Phase Two Study Group. Effect of diet on asthma and allergic sensitisation in the International Study on Allergies and Asthma in Childhood (ISAAC) Phase Two. *Thorax* 2010;65(6):516-522. doi: 10.1136/thx.2009.128256.
9. Rodriguez A, Vaca MG, Chico ME, et al. Rural to urban migration is associated with increased prevalence of childhood wheeze in a Latin-American City. *BMJ Open Respir Res* 2017; 4:e000205.
10. Mpairwe H, Tumwesige P, Namutebi M, et al. Asthma control and management among schoolchildren in urban Uganda: results from a cross-sectional study. *Wellcome Open Res* 2019;4:168. doi: 10.12688/wellcomeopenres.15460.1.
11. Masekela R, Mortimer K, Nantanda R, Lesosky M, Meme H, Devereux G, et al. Asthma care in sub-Saharan Africa: Mind the gap! *J Pan Afr Thorac Soc* 2022;3(2):59-62. doi:10.25259/JPATS_12_2022
12. García-Marcos L, Asher MI, Pearce N, et al.; Global Asthma Network Phase I Study Group. The burden of asthma, hay fever and eczema in children in 25 countries: GAN Phase I study. *Eur Respir J* 2022;60(3):2102866. doi: 10.1183/13993003.02866-2021.
13. Callery P, Milnes L, Verduyn C, Couriel J. Qualitative study of young people's and parents' beliefs about childhood asthma. *Br J Gen Pract* 2003;53(488):185-90. https://bjgp.org/content/bjgp/53/488/185.full.pdf (accessed 03 October 2022)
14. Lakhanpaul M, Culley L, Robertson N, et al. A qualitative study to identify parents' perceptions of and barriers to asthma management in children from South Asian and White British families. *BMC Pulm Med* 2017;17(1):126. doi: 10.1186/s12890-017-0464-9.
15. Chong YY, Leung D, Mak YW. When Control Exacerbates Distress: A Qualitative Study Exploring the Experiences of Hong Kong Chinese Parents in Caring for a Child with Asthma. *Int J Environ Res Public Health* 2018;15(7):1372. doi: 10.3390/ijerph15071372.
16. Chen SH, Huang JL, Yeh KW, Tsai YF. The Stress of Caring for Children With Asthma: A Qualitative Study of Primary Caregivers. *J Nurs Res* 2015;23(4):298-307. doi: 10.1097/JNR.0000000000000083.

For peer review only

17. Kassa E, Kebede RA, Habte BM. Perceptions towards childhood asthma and barriers to its management among patients, caregivers and healthcare providers: a qualitative study from Ethiopia. *BMC Pulm Med* 2022;22(1):184. doi: 10.1186/s12890-022-01984-2.
18. Barakat D, Rockers PC, Vian T, et al. Access to asthma medicines at the household level in eight counties of Kenya. *Int J Tuberc Lung Dis* 2018;22(5):585-590. doi: 10.5588/ijtld.17.0664.
19. Rose S, Paul C, Boyes A, et al. Stigma-related experiences in non-communicable respiratory diseases: A systematic review. *Chron Respir Dis* 2017;14(3):199-216. doi: 10.1177/1479972316680847.
20. Penza-Clyve SM, Mansell C, McQuaid EL. Why don't children take their asthma medications? A qualitative analysis of children's perspectives on adherence. *J Asthma* 2004;41(2):189-97. doi: 10.1081/jas-120026076.
21. Tefera BB, Kibret MA, Molla YB, et al. The interaction of healthcare service quality and community-based health insurance in Ethiopia. *PLoS One* 2021;16(8):e0256132. doi: 10.1371/journal.pone.0256132.
22. Mosler G, Oyenuga V, Addo-Yobo E, et al. Achieving Control of Asthma in Children in Africa (ACACIA): protocol of an observational study of children's lung health in six sub-Saharan African countries. *BMJ Open* 2020;10:e035885. doi:10.1136/ bmjopen-2019-035885
23. Roberts K, Dowell A, Nie JB. Attempting rigour and replicability in thematic analysis of qualitative research data; a case study of codebook development. *BMC Med Res Methodol* 2019;19(1):66. doi: 10.1186/s12874-019-0707-y.
24. Boyatzis RE. Transforming qualitative information: thematic analysis and code development. Thousand oaks CA: SAGE Publications Inc; 1998. ISBN-13: 978-0761909613.
25. Braun V, Clarke V. Using Thematic Analysis in Psychology. Qualitative Research in Psychology 2006;3:77-101. doi: 10.1191/1478088706qp063oa.
26. Lion-Cachet HC, Musonda JMM, Omole OB. Severe asthma in South Africa: A literature review and management approach for primary care. *S Afr Fam Pract* 2021;63(1):e1-e10. Doi: 10.4102/safp.v63i1.5179.

For peer review only

27. Kelada L, Molloy CJ, Hibbert P, et al. Child and caregiver experiences and perceptions of asthma self-management. *NPJ Prim Care Respir Med* 2021;31(1):42. doi: 10.1038/s41533-021-00253-9.

28. World Health Organization. WHO package of essential non-communicable (PEN) disease interventions for primary health care. 07 September 2022. https://apps.who.int/iris/rest/bitstreams/1301957/retrieve (accessed 04 October 2022)

29. Searle A, Jago R, Henderson J, et al. Children's, parents' and health professionals' views on the management of childhood asthma: a qualitative study. *NPJ Prim Care Respir Med* 2017;27(1):53. doi: 10.1038/s41533-017-0053-7.

30. Joseph CL, Williams LK, Ownby DR, et al. Applying epidemiologic concepts of primary, secondary, and tertiary prevention to the elimination of racial disparities in asthma. *J Allergy Clin Immunol* 2006;117(2):233-40; quiz 241-2. doi: 10.1016/j.jaci.2005.11.004.

31. Mphahlele REM, Lesosky M, Masekela R. The prevalence, severity, and risk factors of asthma in South African children . Paper presented at The European Respiratory Society International Congress; PA3953; 2022 Sep 4-6; Barcelona, Spain.

32. Zeitouni MO, Al-Moamary MS, Coussa ML, Riachy M, Mahboub B, AlHuraish F, Zidan MH, Metwally MM, Aksu K, Yavuz E, Kalla IS, Chakaya J, Abdelmadjid S, Ghedira H. Challenges and recommendations for the management of asthma in the Middle East and Africa. *Ann Thorac Med* 2022;17(2):71-80. doi: 10.4103/atm.atm_469_21.

For peer review only - http://bmjopen.bmj.com/site/about/guidelines.xhtml

Table 1: Summary of children with asthma or asthma symptoms and their caregivers who participated in focus group discussions(n=256).

Country (n=total number of focus groups)	Children n=136				Caregivers n=120			
	Children with a doctor-diagnosis of Asthma (CAD)		Children with severe asthma symptoms but no diagnosis (CND)		Caregivers of children with a doctor-diagnosis of asthma (PAD)		Caregivers of children with severe asthma symptoms but no diagnosis. (PND)	
	Focus group (n)	Number of participants	Focus group (n)	Number of participants	Focus group (n)	Number of participants	Focus group (n)	Number of participants
**Malawi, MW (n=4)**	2	8	1	8	1	6	0	0
**Nigeria, NG(n=7)**	2	14	2	12	2	12	1	8
**South Africa, ZA (n=15)**	3	19	3	29	3	21	6	41
**Uganda, UG(n=7)**	2	12	1	6	2	12	2	19
**Zimbabwe, ZW(n=6)**	1	9	3	19	0	0	2	10
**Total participants (n=256)**	**10**	**62**	**10**	**74**	**8**	**51**	**11**	**69**

For peer review only - http://bmjopen.bmj.com/site/about/guidelines.xhtml

637 **Table 2 : Major themes and subthemes and their relationships**

**Major themes**	Understanding Asthma	Reacting to the asthma diagnosis	Managing symptoms and access to healthcare	Asthma and medications
**Subthemes**	• Defining what is asthma<br>• Causes of asthma	• Concerns about delayed diagnosis<br>• Impact on the child being diagnosed<br>• Reactions of significant others to diagnosis	• Identifying and managing triggers<br>• Managing acute asthma episodes<br>• Interacting with the health care system	• Alternative and complementary treatments<br>• Challenges with prescribed medication

638
639
640
641
642
643
644
645

For peer review only - http://bmjopen.bmj.com/site/about/guidelines.xhtml

Table 3: Identified Barriers to asthma care across African countries

Barriers to asthma care across the African countries
1. Inadequate understanding of environmental triggers and causes of asthma<br>2. Reluctance in health care workers to make an asthma diagnosis results in delays and frustration<br>3. Difficulties experienced coping with adjustments in lifestyles<br>4. Difficult and limited access to health care, particularly during an acute asthmatic episode<br>5. Easier access to and acceptance of alternate and complementary asthma treatments compared with prescribed asthma medication<br>6. Preference for prescribed oral asthma medication<br>7. Fears and myths over using metered dose inhalers

Table 4 Recommendations to improve asthma care across Africa

1. Tailor asthma education programmes to clarify the role of environmental triggers in asthma and the need for medications for asthma control.<br>2. Modify the current risk determinants for asthma triggers to be relevant to African contexts.<br>3. Sensitise school-going children to provide support with inhaler usage to affected peers.<br>4. Engage traditional medical systems to propagate the use of inhaler asthma therapies and devices<br>5. Simplify the diagnostic pathway of childhood asthma for health care workers<br>6. Improve access to acute and chronic asthma care in primary health care settings<br>7. Ensure universal access to and knowledge of using combination inhalers, where appropriate, to reduce SABA over-reliance and the preference for prescribed oral asthma medications across Africa.

For peer review only

acacia achieving control of asthma in children in africa

UNIVERSITY OF KWAZULU-NATAL INYUVESI YAKWAZULU-NATALI

ACACIA Breathing Survey

QR code label or Participant ID:

____

Introduction

We are a group of researchers interested in healthy breathing. In the questions below we will ask if you sometimes make a noise when you breathe, which is called wheezing. Wheezing is a whistling sound from the chest that some people make when they breathe.

Section 1: Personal Details

1. **What is your name?** ______________________________

2. **Today's date:** DAY / MONTH / YEAR

3. **School:** ______________________________

4. **Are you male or female?**

   ☐ Male

   ☐ Female

5. **How old are you?** _________

6. **What is your date of birth?** DAY / MONTH / YEAR

7. **Which school class or school year are you in?** ______________________________

8. **How would you describe your ethnicity?**

   ☐ Black

   ☐ White

   ☐ East Asian, such as Chinese, Japanese, or Korean

   ☐ South Asian, such as Indian, or Bangladeshi

   ☐ Mixed race, please specify: ______________________

   ☐ Other, please specify: __________________________

For peer review only

NIHR | National Institute for Health Research

Queen Mary University of London

acacia
achieving control of asthma in children in africa

UNIVERSITY OF KWAZULU-NATAL
INYUVESI YAKWAZULU-NATALI

Section 2: Breathing

1. **Have you had wheezing or whistling in the chest in the past 12 months?**

☐ Yes

☐ No

IF YOU HAVE ANSWERED "NO" PLEASE SKIP TO QUESTION 5

2. **How many attacks of wheezing have you had in the past 12 months?**

☐ None

☐ 1 to 3

☐ 4 to 12

☐ More than 12

3. **In the past 12 months, how often, on average, has your sleep been disturbed due to wheezing?**

☐ Never woken with wheezing

☐ Less than one night per week

☐ One or more nights per week

4. **In the past 12 months, has wheezing ever been severe enough to limit your speech to only one or two words at a time between breaths?**

☐ Yes

☐ No

5. **Have you ever had asthma?**

☐ Yes

☐ No

IF YOU HAVE ANSWERED YES,

6. **Was your asthma confirmed by a doctor?**

☐ Yes

☐ No

For peer review only

FGD 1: Children (12-14 yrs) with severe asthma and diagnosis of asthma

Before the discussion

1. Introduction
2. Game/warming up exercise

Discussion

1. Starter activity: Go around the room, everyone should finish the sentence: 'Asthma is…

   (The facilitators start, e.g. Asthma is… not easy/ hard to talk about/ limiting/ not a problem…)

2. How much do breathing problems affect what you do?
   - … At school?
   - … At home?
   - … Anywhere else?
3. If you have asthma medication, how do you take your asthma medications?
   - What helps you take your asthma medication?
   - What makes it difficult to take your asthma medication?
   - What do you dislike about your asthma medication?
   - Is there any other medication that you think helps you? For example traditional medication?
   - What are some of the ideas you have to make it easier to take your asthma medication?
4. How do you feel if you have difficulties breathing?
   - … When you are around your friends?
     - How do they react?
   - … When you are in class?
     - How does the teacher react?
5. What do you believe people around you think about your asthma?
   - Friends,
   - family,
   - teachers
   - What do you tell people about your asthma? And who do you talk to?

For peer review only - http://bmjopen.bmj.com/site/about/guidelines.xhtml

(Optional) Questions about health interventions:

1. Do you have any experience of health-related activities taking place at the school?
    a. Prompt: for example relating to healthy lifestyles, TB, HIV, malaria…..
    b. If yes, please tell us about what happened.
    c. How did you find it?

2. Do you have any suggestions for activities that could take place at the school, to support young people with asthma?
    a. Prompt: some ideas to discuss might be peer support group, health club, visiting "school nurse"

For peer review only

For peer review only - http://bmjopen.bmj.com/site/about/guidelines.xhtml

acacia achieving control of asthma in children in africa

Page | 3

FGD 2: Children (12-14yrs) with severe asthma and no diagnosis

Before the discussion:

1. Introduction
2. Game/warming up exercise

Discussion:

1. What would you do if you have difficulties breathing?
   - Can you think of anything that might make it easier to breathe?
   - Do you think there is anything that might make it worse?
2. Have you ever taken any medication or remedies for your breathing difficulty?
   - Tell me about it.
3. How much do breathing problems affect what you do?
   - … At school?
   - … At home?
   - … Anywhere else?
4. How do you feel if you have difficulties breathing...?
   - … When you are around your friends?
     - How do you think they would react?
   - … When you are in class?
     - How has the teacher reacted?
5. Have you heard of asthma?
   - What do you think does it mean to have asthma?
6. What would you think if a very good friend suddenly tells you that he/she has asthma?

(Optional) Questions about health interventions:

1. Do you have any experience of health-related activities taking place at the school?
   a. Prompt: for example relating to healthy lifestyles, TB, HIV, malaria…..
   b. If yes, please tell us about what happened.
   c. How did you find it?

2. Do you have any suggestions for activities that could take place at the school, to support young people with asthma?
   a. Prompt: some ideas to discuss might be peer support group, health club, visiting "school nurse"

For peer review only - http://bmjopen.bmj.com/site/about/guidelines.xhtml

acacia achieving control of asthma in children in africa

Page | 4

FGD 3: Parents of children with diagnosed asthma

Before the discussion

1. Introduction
2. Warm up exercise / tea & cookies
3. Short questionnaire
   - Knowledge questions (symptoms of asthma/triggers etc.)
   - Questions about beliefs around asthma (e.g. How much support do you think a person with asthma needs? A lot,…, none)
   - Where do you go to seek help?

Discussion

1. Asthma is… (finish sentence)
2. Tell us about your experience with asthma?
   - Tell us about your experience with healthcare
     – diagnosis
   - … Experience with asthma treatment (inhalers, alternative treatment methods etc.)
   - Tell us about any impact the asthma diagnosis had for you and your family
     – Financially
     – Socially
3. Can you tell us what makes it difficult for your child to take the asthma medication?
4. What do you think it means for your child to have asthma?
   - Would you have any special concerns about your child because of their asthma?
     – Would you think there is anything that an asthmatic child should avoid?
5. Do you have any examples of what makes life with asthma difficult for your child?
6. Is there anything that helps your child to live with asthma, apart from medication?

(Optional) Questions about health interventions:

1. Do you know of any health-related activities taking place at your child's school?
   a. Prompt: for example relating to healthy lifestyles, TB, HIV, malaria…..
   b. If yes, please tell us about what you know about it.

2. Do you have any suggestions for activities that could take place at the school, to support young people with asthma?
   a. Prompt: some ideas to discuss might be peer support group, health club, visiting "school nurse", workshops for teaching staff

For peer review only - http://bmjopen.bmj.com/site/about/guidelines.xhtml

acacia achieving control of asthma in children in africa

FGD 4: Parents of children without asthma

Before the discussion

1. Introduction
2. Warm up exercise/ tea & cookies
3. Short questionnaire
   - Knowledge questions (symptoms of asthma/triggers etc.)
   - Questions about beliefs around asthma (e.g. How much support do you think a person with asthma needs? A lot,…, none)
   - If your child had problems to breathe sometimes, where would you go to seek help?

Discussion

1. How would you describe asthma?
   - Tell us about your experience with asthma (Do you know anyone with asthma?)
   - If you hear that your child's friend had asthma, what would you say to your child?
2. If you are told that your child has asthma,
   - How would you react?
   - What would you tell your child?
   - Where would you seek help?
   - How would you manage?
   - How would you feel?
3. Why do you think someone might be worried about asthma?
   - Who do you think might be affected by asthma?

For peer review only

(Optional) Questions about health interventions:

3. Do you know of any health-related activities taking place at your child's school?
   a. Prompt: for example relating to healthy lifestyles, TB, HIV, malaria…..
   b. If yes, please tell us about what you know about it.

4. Do you have any suggestions for activities that could take place at the school, to support young people with asthma?
   a. Prompt: some ideas to discuss might be peer support group, health club, visiting "school nurse", workshops for teaching staff

For peer review only - http://bmjopen.bmj.com/site/about/guidelines.xhtml

acacia
achieving control of
asthma in children in africa

FGD 5: Teachers

Before the discussion

1. Introduction
2. Warm up exercise/ tea & cookies
3. Short questionnaire
    - Knowledge questions (symptoms of asthma/triggers etc.)
    - Questions about beliefs around asthma (e.g. how much support do you think a person with asthma needs? A lot,…, none)
    - Have you had any training/learnt about asthma?

Discussion

1. Asthma is… (finish the sentence)
2. Tell us about your experience with asthma in school?
    - What would you do if a child has an asthma attack in school?
    - Tell us about your experiences of asthma outside of school.
3. What do you think will improve your confidence to help a child when they have an asthma attack in school?
4. In your experience, how does asthma affect a child in school?
5. What are some of your ideas how to improve life for children with asthma?

(Optional) Questions about health interventions:

5. Do you have any experience of health-related activities taking place at the school?
    a. Prompt: for example relating to healthy lifestyles, TB, HIV, malaria…..
    b. If yes, please tell us about what happened.
    c. How did you find it?

6. Do you have any suggestions for activities that could take place at the school, to support young people with asthma?
    a. Prompt: some ideas to discuss might be peer support group, health club, visiting "school nurse", workshops for teaching staff.

For peer review only - http://bmjopen.bmj.com/site/about/guidelines.xhtml

FGD 1: Children (12-14 yrs.) with severe asthma and diagnosis of asthma		
**Code**	**Definition**	**Purpose/meaning of code**
1. Definition of asthma	Participant defines asthma	Indicates participant's knowledge of what asthma is<br>Indicates Knowledge
2. Effects of breathing difficulties	Participant describes their own experience of how their asthma affects them when they are in various environments such as school, home and anywhere else	Participant indicates how much their breathing problems alter their activities in different environments.
3. Positive experience with asthma medication	participants describe what is pleasing to them about their medications and why they use them	participant describe what improves their symptoms<br>what makes it easy for them to use the medications
4. Negative experience with asthma medication	Participant describe what is not pleasing about their medication and why they do not like to use them.	What make is difficult to take their medication, can be taste, smell, stigma attached to taking medication in public
5. Alternative medication	Participant indicates and describes an experience of taking any alternative medication for their asthma	Indicate alternative medication/methods or idea to relieve their symptoms. Can be at school, home or anywhere else
6. reaction of friends to an asthma attack	How their friends have reacted to them while having an asthma attack and how they felt about their friend's reaction	Determine if there are perceived stigma limiting expression of symptoms. Determine the extent to which peers could provide support or otherwise
7. Reaction to having an asthmatic attack teachers	Participant recalls or describes how their teachers reacted when they were having an asthma attack and how they felt when that was happening	How they perceive getting help from the teachers. Finding out the aid that is offered to the learner and how teachers respond/react to asthmatic learner
8. Perception of asthma from friends	Describe whether friends are aware that they have asthma or not? Describe how the friends feel about having an asthma sufferer as a friend	Describe friends' reaction to them having asthma.<br>how has the relationship being impacted
9. Perception of asthma from family	Participants describe what they family think or feel about their asthma	Are they treated differently in their family because of their asthma? How does their family react when they exhibit symptoms? Have there been any changes to the household since their diagnosis?
10. Perceptions of asthma from teachers	Participants describe what their teachers feel about them having asthma	Do their teachers know they have asthma? What kind of assistance do they receive from the teachers to reduce risk of developing symptoms in school?<br>What kind of assistance do they receive when they exhibit asthma symptoms?
11. Personal perceptions about your asthma	Participants describe how they feel about their own asthma, what are their experiences around it	We are trying to find out how they feel or think about their asthma, what are their experiences around it, good or bad

For peer review only

FGD 2: Children (12-14yrs) with severe asthma and no diagnosis		
**Code**	**Definition**	**Purpose/meaning of code**
1. Easing breathing difficulties	Participants describe what makes it easier for them to breath when they have trouble breathing	What are their coping mechanisms, what do they do in order for their symptoms not to get worse
2. Exacerbations of breathing difficulties	Participants describe what makes it worse for them when they have difficulties breathing	identify things that make their symptom worse
3. Medications	Participants indicate if they have taken any medications for their symptoms or any alternative methods to help them with their symptoms. Identify medication use : type	Finding out what medications and remedies they have used. Describe what is done to bring relief
4. Effects of experiencing breathing problem	Participant describes their experience of how their asthma affects them when they are in various environments such as school, home and anywhere else	description of experience of asthma symptoms- home, school etc.
5. Reaction from friends	Participant recalls and describe how their friends reacted when they were having an asthma attack and how they felt when that was happening	How they perceive getting help from their peers. Finding out they experience any stigma from exhibiting breathing difficulties in front of their friends
6. Reaction from teachers	Participant recalls and describe how their teachers reacted when they were having an asthma attack and how they felt when that was happening	How they perceive the help or otherwise from school. describe what was done and the teacher's reaction
7. Perceptions of having breathing problems /asthma	Participant perception of what asthma is and how they will react if a friend have asthma	Definition of asthma and perception of it

For peer review only

FGD 3: Parents of children with diagnosed asthma		
**Code**	**Definition**	**Purpose/meaning of code**
Definition of asthma	Participant defines asthma/ describe asthma symptoms	Indicates participant's knowledge of what asthma is
1. Response to initial asthma diagnosis	Participant describes their experience when their child was diagnosed with asthma	Describe parents' initial reaction to asthma diagnosis
2. Experience with healthcare with regards asthma		Journey to diagnosis of asthma in healthcare
3. Positive experiences asthma medication	Participants describes their experience with asthma treatment	describe parents knowledge about asthma treatment and support provided when child was diagnosed
4. Negative experience with asthma medication	Participant describes negative aspects of asthma medication. What they don't like about the medication	What make is difficult to for their children to take the medication, can be taste, smell, stigma attached to taking medication in public
5. Financial impact of asthma diagnosis	Cost of medication, availability of medication at their local healthcare facility, fear of side effects	determine the financial implication (direct and indirect cost of asthma care)
6. Social implications of the diagnosis	Social implications of the asthma diagnosis on their children and family	Try to find out the perceptions and attitude to asthma in the community they live in or perceptions from their family. Find out the if they have experienced stigma or support
7. Concerns around asthma	What it means for their child to have asthma. Their perceptions and worries around the diagnosis and care of asthma	How people would Perceive or treat their child, how the child would cope if not at home etc.
8. enablers in asthma care of children	Participant describes what makes it easier for their child with an asthma diagnosis to be cared for	Parents perception of what will make life easier for a child with asthma
9. Difficulties of an asthma diagnosis	Participant describes what has been difficult since the asthma diagnosis	Parents perception of what has made life difficult for a child with asthma

For peer review only

FGD 4: Parents of children without asthma		
**Codes**	**Definition**	**Purpose/meaning code**
1. Knowledge of breathing problems in children	Participant recalls and describes their experience and knowledge around asthma	Try to find out their knowledge around asthma and if they know someone with asthma
2. Perception of asthma in other children	Their reactions if their child's friend had asthma	Try to find out their perception around asthma, fear, stigma etc.
3. Reaction if their child had asthma	Their reaction if their child was diagnosed with asthma	Try to find out their perception and feeling about asthma
4. communicating with child on asthma	How would they tell their child about the diagnosis	Try to find out their knowledge around asthma or what resources they would use to tell the child more about asthma. How would they communicate asthma information to their child
5. Seeking help if their child had asthma	Where would they go to seek help for asthma, asthma care, treatment and information	Try to find out if they have access to resources that can help them and their child. What type kind of resources are available to them in case of emergencies<br>Where they would seek help i.e. from people, places
6. Manage asthma care/Breathing problems	Participant describe what they would do differently if their child was diagnosed with asthma and how they would care for them	How would they manage the care of their child who has asthma- things they would do in terms of care
7. Positive Feelings on asthma	Their perceptions around asthma	any positive feeling described
8. Negative feelings /Concerns around asthma	Participant describes their concerns about asthma or an asthmatic's concern about asthma	Describes such feeling as fear, afraid of stigma, afraid of financial burden, burden of visiting hospital frequently, fear for sudden asthma attacks or what the child may not be able to do.

For peer review only

FGD 5: Teachers		
**Code**	**Definition**	**Purpose/meaning of code**
1. knowledge of asthma	Participant defines asthma/ describe asthma symptoms	Indicates participant's knowledge of what asthma is
2. Beliefs	Teachers own beliefs on what asthma is	States teacher's personal belief on asthma this is not in guide
3. Experience of asthma in school	Participant recalls or describes an experience they have had when a learner had an asthma attack in their class	Try to find out their response to an emergency, what measures are in place to help a child with asthma
4. Experience of asthma outside of school	Participant recalls or describes their experience with an asthmatic person	if they know anyone with asthma., presentation, what was done, Also try and find out their perceptions of people with asthma
5. Care for asthmatic child	Participant describes what they would need in order to attend or help an asthmatic in class	Try to find out what access or information they need to better take care of a child with asthma
6. Effects of asthma on schooling	Participant describes from their experience how asthma has affected the learning process of the student	Try to find out how asthma has affect the student in terms of their learning, absenteeism, participant in social activities – sports, behaviour and relationship with other students
7. enablers to asthma care in school	Participant suggest how asthmatic children's lives can be made better inside and outside of school	Try to find out what can be done by school, parents, and teachers, healthcare to improve the life of an asthmatic child. What kind of tools or information needs to be available

For peer review only

COREQ Qualitative research checklist :
Barriers to Childhood asthma care in Sub-Saharan Africa: a multi-country qualitative study with children and their caregivers.

No Item Guide	questions/description	
**Domain 1:**	**Research team and reflexivity**	NB We attempted to ensure a fair and equitable coding team to decrease in-country biases<br>We used an inductive approach with constant data comparisons and discussions to inform our understanding of the views expressed. We were aware of the risk of researcher bias in qualitative research and actively discussed reflexivity in the regular multidisciplinary team discussions, which aided analysis and informed interpretation.
**Personal Characteristics**		
1. Interviewer/facilitator?	Which author/s conducted the interview or focus group?	All focus group discussions (FGDs) were moderated by at least two facilitators, one of whom was a study investigator at each site.<br>Reference is provided for the protocol
2. Credentials	What were the researcher's credentials? E.g. PhD, MD	"one of whom was a study investigator at each site', Research project has a list of credentials of all investigators- reference for the published protocol included
3. Occupation	What was their occupation at the time of the study?	Research project has a list of credentials of all investigators- reference for the published protocol included
4. Gender	Was the researcher male or female?	All genders .multiple researchers
5. Experience and training	What experience or training did the researcher have?	Research project has a list of credentials of all investigators- reference for the published protocol included
**Relationship with participants**		
6. Relationship established Was a relationship established prior to study commencement?	Was a relationship established prior to study commencement?	The details of the larger study are provided. 'Children and their caregivers were recruited initially as part of the ACACIA dataset'
7. Participant knowledge of the interviewer	What did the participants know about the researcher? e.g. personal goals, reasons for doing the	The researchers<br>All moderators introduced themselves at all FGDs. The standardised interview guide included a formal introductions component<br>Participants provided written informed consent as well as informed caregiver (parent /guardian) consent and child assent in accordance with both country/local regulations and United Kingdom (UK) ethics regulations.- this process provided information on the goals of both the larger project and the FGDs

For peer review only - http://bmjopen.bmj.com/site/about/guidelines.xhtml

COREQ Qualitative research checklist :
Barriers to Childhood asthma care in Sub-Saharan Africa: a multi-country qualitative study with children and their caregivers.

8. Interviewer characteristics,	What characteristics were reported about the interviewer/facilitator? e.g. Bias, assumptions, reasons and interests in the research topic	There were multiple facilitators across all countries. Focus groups were conducted face-to-face, with adherence to in-country-specific COVID-19 regulations. All FGDs were moderated by at least two facilitators, one of whom was a study investigator at each site.<br>Participants provided written informed consent as well as informed caregiver (parent /guardian) consent and child assent in accordance with both country/local regulations and United Kingdom (UK) ethics regulations.- this process provided information on the goals of both the larger project and the FGDs
**Domain 2: study design**		
**Theoretical framework**		
9. Methodological orientation and theory	What methodological orientation was stated to underpin the study? e.g. grounded theory, discourse analysis, ethnography, phenomenology, content analysis	This study was across 6 countries and used an inductive analysis to examine the data.[23] The methodological orientation was largely content analysis using a codebook analysis due to the large number of transcripts .We have provided references for the process we followed with the code book development<br>Ten members of the ACACIA research group from all the participating countries independently developed codes from the transcripts and then discussed the coding to develop a code book.[23,24] At least two investigators reviewed each transcript. A reiterative process was followed to apply the codebook to the transcribed data and determine whether further codes emerged. When no new codes emerged, the codebook was assumed to be a valid representation of the data and finalised
**Participant selection**		
10. Sampling	How were participants selected? e.g. purposive, convenience, consecutive, snowball	Children and their caregivers were recruited initially as part of the ACACIA dataset. A Breathing Survey developed from questions in the Global Asthma Network (GAN) questionnaire (Supplementary file 1)was used to identify participants between 12 and 14 years with asthma symptoms or an asthma diagnosis.[22] Focus group participants (both children and their caregivers) were recruited using purposive sampling and categorised into four groups; children with a doctor-diagnosis of asthma (CAD) and their parents/caregivers (PAD), children with severe wheezing

For peer review only - http://bmjopen.bmj.com/site/about/guidelines.xhtml

COREQ Qualitative research checklist :

Barriers to Childhood asthma care in Sub-Saharan Africa: a multi-country qualitative study with children and their caregivers.

		symptoms without a doctor-diagnosis of asthma (CND) and their parents/caregivers (PND), with FGDs held separately for each category
11. Method of approach, mail, email	How were participants approached? e.g. face-to-face, telephone, mail, email	Participants were approached face to face. Focus groups were conducted face-to-face, with adherence to in-country-specific COVID-19 regulations.
12. Sample size	How many participants were in the study?	A total of 136 children were included
13. Non-participation	How many people refused to participate or dropped out? Reasons?	We did not keep data on the refusals or non-participation across countries
**Setting**		
14. Setting of data collection	Where was the data collected? e.g. home, clinic, workplace	At schools where the larger study was being carried out
15. Presence of non-participants	Was anyone else present besides the participants and researchers?	Yes teachers. However the teachers did not join the children or caregivers FGDs. We conducted separate teachers FGDs
**Data collection**		
17. Interview guide	Were questions, prompts, guides provided by the authors? Was it pilot tested?	Discussions lasted between 40-60 minutes and followed a predefined question guide specific to each focus group category. The discussion guides were developed after multiple iterations by the ACACIA core study team and informed by the study aims and literature review on barriers to asthma care. (Supplementary file 2)
18. Repeat interviews carried out?	Were repeat interviews carried out? If yes, how many?	No repeat interviews were done
19. Audio/visual recording	Did the research use audio or visual recording to collect the data?	Focus groups were audio-recorded, transcribed verbatim and translated to English if needed in the country.
20. Field notes	Were field notes made during and/or after the interview or focus group?	Yes this was done in the various countries
21. Duration	What was the duration of the interviews or focus group?	Discussions lasted between 40-60 minutes and followed a predefined question guide specific to each focus group category.
22. Data saturation	Was data saturation discussed?	Yes at two points<br>1. Code book development: A reiterative process was followed to apply the codebook to the transcribed data and determine whether further codes emerged. When no new codes emerged, the codebook was assumed to be a valid representation of the data and finalised (Supplementary 3).

For peer review only - http://bmjopen.bmj.com/site/about/guidelines.xhtml

COREQ Qualitative research checklist :
Barriers to Childhood asthma care in Sub-Saharan Africa: a multi-country qualitative study with children and their caregivers.

		2. Data analysis: Major themes and subthemes were identified based on patterns and relationships noted between derived codes, and all investigators agreed upon these after multiple iterations.
23. Transcripts returned	Were transcripts returned to participants for comment and/or correction?	No. We did not conduct member checks. We have listed this in the limitations
**Domain 3: analysis and findings**		
**Data analysis**		
24. Number of data coders	How many data coders coded the data?	Ten members of the ACACIA research group from all the participating countries independently developed codes from the transcripts and then discussed the coding to develop a code book.[23,24]
25. Description of the coding tree	Did authors provide a description of the coding tree?	Yes. We used an inductive analysis to examine the data.[23] Ten members of the ACACIA research group from all the participating countries independently developed codes from the transcripts and then discussed the coding to develop a code book.[23,24] At least two investigators reviewed each transcript. A reiterative process was followed to apply the codebook to the transcribed data and determine whether further codes emerged. When no new codes emerged, the codebook was assumed to be a valid representation of the data and finalised (Supplementary 3).
26. Derivation of themes	Were themes identified in advance or derived from the data?	We used an inductive analysis to examine the data.[23]
27. Software	What software, if applicable, was used to manage the data?	The transcripts taken during the discussion were used to analyse the data using the NVivo 1.6 software program.
28. Participant checking	Did participants provide feedback on the findings?	No.
**Reporting**		
29. Quotations presented	29. Quotations presented Were participant quotations presented to illustrate the themes / findings? Was each quotation identified? e.g. participant number	Yes .each quote is identified using the country origin and category of the FGD and group number e.g. (CAD, MW, Group1); (CAD, ZW, Group1)
30. Data and findings consistent	Was there consistency between the data presented and the findings?	Yes we have attempted to present the data to support the findings.

For peer review only - http://bmjopen.bmj.com/site/about/guidelines.xhtml

COREQ Qualitative research checklist :
Barriers to Childhood asthma care in Sub-Saharan Africa: a multi-country qualitative study with children and their caregivers.

31.Clarity of major themes	Were major themes clearly presented in the findings?	Yes the themes are presented clearly and tables are used to support this
32. Clarity of minor themes	Is there a description of diverse cases or discussion of minor themes?	With the multiple countries a major aim of the research was to focus on common (cross cutting) themes. However nuanced themes specific to countries are noted<br><br>The themes that were common across countries were specifically identified and highlighted and where specific in-country views differed these were noted.

For peer review only

For peer review only - http://bmjopen.bmj.com/site/about/guidelines.xhtml

BMJ Open

Barriers to Childhood asthma care in Sub-Saharan Africa: a multi-country qualitative study with children and their caregivers.

Journal:	*BMJ Open*
Manuscript ID	bmjopen-2022-070784.R1
Article Type:	Original research
Date Submitted by the Author:	14-Jun-2023
Complete List of Authors:	Naidoo, Kimesh; King Edward VIII Hospital, Nelson R Mandela School of Medicine University of KwaZulu-Natal, Paediatrics and Child health<br>Dladla, Sindiswa; College of Health Sciences University of KwaZulu-Natal, Department of Paediatrics and Child Health<br>Mphahlele, Reratilwe; University of KwaZulu-Natal Nelson R Mandela School of Medicine, Paediatrics and Child Health<br>Mosler, Gioia; Queen Mary University of London, Centre for Genomics and Child Health<br>Muyemayema, S; University of Zimbabwe, Department of Primary Health Care Sciences<br>Ssemata, Andrew; Makerere University College of Health Sciences, Psychiatry<br>Mkutumula, E; College of Medicine, 6. Malawi Liverpool Wellcome Programme, Queen Elizabeth Central Hospital, College of Medicine, Chichiri, Malawi<br>Adeyeye, Olayinka; Lagos State University College of Medicine<br>Moyo, Melinda ; University of Zimbabwe, 3. Child and Adolescent Health Unit (CAHU), Department of Primary Health Care Sciences<br>Goodman, Y; Lagos State University College of Medicine Ikeja; Lagos State University Teaching Hospital<br>Kuyinu, Y; Lagos State University College of Medicine Ikeja; Lagos State University Teaching Hospital<br>Nantanda, Rebecca; Makerere University, Lung Institute, Makerere College of Health Sciences<br>Addo-Yobo, Emmanuel; Kwame Nkrumah University of Science and Technology, School of Medicine and Dentistry, College of Health Sciences<br>Ticklay, Ismail; University of Zimbabwe College of Health Sciences, Paediatrics and Child Health; University of Zimbabwe College of Health Sciences, Medical School Parirenyatwa Hospital<br>HA, Mujuru; University of Zimbabwe, Department of Primary Health Care Sciences<br>Grigg, Jonathan; Queen Mary University London, Paediatric Respiratory<br>Masekela, R ; University of KwaZulu-Natal College of Health Sciences, Paediatrics and Child Health
**Primary Subject Heading**:	Paediatrics
Secondary Subject Heading:	Respiratory medicine

Keywords:	Asthma < THORACIC MEDICINE, PAEDIATRICS, RESPIRATORY MEDICINE (see Thoracic Medicine), PUBLIC HEALTH

SCHOLARONE™ Manuscripts

I, the Submitting Author has the right to grant and does grant on behalf of all authors of the Work (as defined in the below author licence), an exclusive licence and/or a non-exclusive licence for contributions from authors who are: i) UK Crown employees; ii) where BMJ has agreed a CC-BY licence shall apply, and/or iii) in accordance with the terms applicable for US Federal Government officers or employees acting as part of their official duties; on a worldwide, perpetual, irrevocable, royalty-free basis to BMJ Publishing Group Ltd ("BMJ") its licensees and where the relevant Journal is co-owned by BMJ to the co-owners of the Journal, to publish the Work in this journal and any other BMJ products and to exploit all rights, as set out in our licence.

The Submitting Author accepts and understands that any supply made under these terms is made by BMJ to the Submitting Author unless you are acting as an employee on behalf of your employer or a postgraduate student of an affiliated institution which is paying any applicable article publishing charge ("APC") for Open Access articles. Where the Submitting Author wishes to make the Work available on an Open Access basis (and intends to pay the relevant APC), the terms of reuse of such Open Access shall be governed by a Creative Commons licence – details of these licences and which Creative Commons licence will apply to this Work are set out in our licence referred to above.

Other than as permitted in any relevant BMJ Author's Self Archiving Policies, I confirm this Work has not been accepted for publication elsewhere, is not being considered for publication elsewhere and does not duplicate material already published. I confirm all authors consent to publication of this Work and authorise the granting of this licence.

For peer review only

Title: Barriers to Childhood asthma care in Sub-Saharan Africa: a multi-country qualitative study with children and their caregivers.

Authors:

Naidoo KL, PhD[1]; Dladla S[1]; Mphahlele REM[1]; Mosler G[2], PhD; Muyemayema S[3]; Ssemata AS[4 5]; Mkutumula E[6]; Adeyeye O[7 8]; Moyo M[3]; Goodman Y[7 8]; Kuyinu Y[7 8]; Nantanda R[9 10]; Addo-Yobo E[11]; Ticklay I [3 12]; Mujuru HA[3]; Grigg J[2]* , PhD; Masekela R[1] , PhD*

1. Department of Paediatrics and Child Health, School of Clinical Medicine, College of Health Sciences, University of KwaZulu-Natal
2. Centre for Genomics and Child Health, Blizzard Institute, Barts and The London School of Medicine and Dentistry, Queen Mary University of London, London, UK
3. Child and Adolescent Health Unit (CAHU), Department of Primary Health Care Sciences, Faculty of Medicine and Health Sciences, University of Zimbabwe, Avondale, Harare, Zimbabwe
4. Department of Psychiatry, College of Health Sciences, Makerere University, Kampala, Uganda
5. Department of Global Health and Development, London School of Hygiene and Tropical Medicine, London, UK
6. Malawi Liverpool Wellcome Programme, Queen Elizabeth Central Hospital, College of Medicine, Chichiri , Malawi
7. Lagos State University College of Medicine Ikeja, Lagos, Nigeria
8. Lagos State University Teaching Hospital, Ikeja, Lagos, Nigeria
9. Makerere University Lung Institute, Makerere University College of Health Sciences, Kampala, Uganda
10. Department of Paediatrics and Child Health, Makerere University College of Health Sciences, Kampala, Uganda
11. Department of Child Health, Komfo Anokye Teaching Hospital, Kumasi, Ghana
12. Parirenyatwa Group of Hospitals, Causeway, Harare, Zimbabwe

For peer review only - http://bmjopen.bmj.com/site/about/guidelines.xhtml

Corresponding author
Dr KL Naidoo
Department of Paediatrics and Child Health, School of Clinical Medicine, College of Health Sciences, University of KwaZulu-Natal, 719 Umbilo Road, Congella, Durban South Africa
Email: naidook9@ukzn.ac.za
Telephone: 027 312604350
Fax: 027312604388

Five Key words: paediatrics, asthma, adolescence, children, sub-Saharan Africa.

Word count: Main manuscript word count = 4319 (excluding title page, abstract, references, figures and tables)

For peer review only

For peer review only - http://bmjopen.bmj.com/site/about/guidelines.xhtml

ABSTRACT – word count = 297

Objectives

This study identifies barriers and provides recommendations to improve asthma care in children across sub-Saharan Africa, where qualitative data is lacking despite high rates.

Design

One of the aims of our National Institute for Health Research global health research group "Achieving Control of Asthma in Children in Africa" (ACACIA) was to use qualitative thematic analysis of transcribed audio recordings from focus group discussions (FGDs) to describe barriers to achieving good asthma control.

Setting

Schools in Blantyre (Malawi), Lagos (Nigeria), Durban (South Africa), Kampala (Uganda), and Harare (Zimbabwe).

Participants

Children (n=136), 12 to 14 years with either asthma symptoms or a diagnosis and their caregivers participated in 39 FGDs. All were recruited using asthma control questions from the Global Asthma Network survey.

Results

There were four key themes identified: (1) Poor understanding, (2) Difficulties experienced with being diagnosed, (3) Challenges with caring for children experiencing an acute asthma episode and (4) Sub-optimal uptake and use of prescribed medicines. An inadequate understanding of environmental triggers, a hesitancy in using metered dose inhalers and a preference for oral and alternate medications were identified as barriers. In addition, limited access to health care with delays in diagnosis and an inability to cope with expected lifestyle changes was reported. Based on these findings, we recommend tailored education to promote access to and acceptance of metered dose inhalers, including advocating for access to a single therapeutic, preventative and treatment option. Furthermore, healthcare systems should have simpler diagnostic pathways and easier emergency access for asthma.

Conclusions

In a continent with rapidly increasing levels of poorly controlled asthma, we identified multiple barriers to achieving good asthma control along the trajectory of care. Exploration of these barriers reveals several generalisable recommendations that should modify asthma care plans and potentially transform asthma care in Africa.

Trial registration number 269211.

For peer review only - http://bmjopen.bmj.com/site/about/guidelines.xhtml

Key messages statements /strengths and limitations

- This is a first cross African, multi-country qualitative study that used thematic analysis to identify barriers to asthma care
- Perspectives of both adolescents and their caregivers on asthma diagnosis and management are included
- Views of both children with a physician diagnosis of asthma and those with symptoms of asthma and not formally diagnosed are included.

Keywords: children, poor control, Childhood asthma, sub-Saharan Africa.

Funding statement

This research was funded by the National Institute for Health Research (NIHR) (Trial registration number 269211) using UK aid from the UK Government to support global health research.

Disclaimer The views expressed in this publication are those of the author(s) and not necessarily those of the NIHR or the UK Department of Health and Social Care (17/63/38).

INTRODUCTION

Globally, asthma is the most common non-communicable respiratory disease among children.[1] while the prevalence of asthma in African children is currently 14% and matches the global average, it is increasing in low-middle income countries (LMIC) with different trajectories suggested by the wide range of prevalence between countries, for example, Kenya at 15.8%, Nigeria 13.0%, and South Africa 20.3%. [1-4] The goal of asthma care is to optimise symptom control and maintain a good quality of life.[5, 6] Asthma control has been reported to be poor with the 2018 Global Asthma Report reporting high uncontrolled asthma prevalence rates across sub-Saharan Africa (sSA).[4] The determinants of poor asthma control in sSA are multifactorial including environmental factors which are certainly important and include rural to urban migration, early life urban residence, overcrowding, poorer air quality, lifestyle changes and diet.[6-9] Patient-related factors reported in African children include poor knowledge and symptom recognition, non-acceptance of inhaler treatment, older age and concurrent allergic rhinitis.[6,7] Health system factors include limited diagnosis of asthma, episodic management of

For peer review only - http://bmjopen.bmj.com/site/about/guidelines.xhtml

asthma symptoms at the primary health care (PHC) level, and poor access to inhaled corticosteroids.[5,6,9-12]. However, to date, there is little qualitative data from children and young people on the determinants of asthma control in this region. Studies in high-income countries have found that children's and caregivers' health beliefs about asthma and poor relationships with healthcare providers (HCPs) can undermine asthma management.[13] Caregivers have also reported fear, feeling of powerlessness, uncertainty, and social tension related to their children who have asthma.[14-16] Studies from individual African countries have also identified physical, emotional and social burdens related to childhood asthma, and these have been postulated to affect non-adherence to recommended treatment regimens.[10,17-19]

Understanding the views of caregivers and affected children with doctor-diagnosed asthma and those with symptoms suggestive of asthma are needed to capture perspectives effectively. To accurately and efficiently determine strategies to improve asthma care, recommendations need to be identified and explored, that are derived from the perspectives of affected African children and their caregiver's concerns .[20] A richer, qualitative understanding of identified barriers to asthma care in African countries will assist with the development of relevant advocacy and policy interventions across the continent.[21] This study aimed to capture the views of children with asthma/asthma symptoms with and their caregivers, in sSA and to identify barriers to asthma diagnosis and care as well as interventions to improve asthma care across countries in Africa.

METHODS

Study design

This study was a component of the achieving control of the National Institute for Health Research (NIHR)funded global health research group's "Achieving control of asthma in children in Africa" (ACACIA), a larger observational cohort study across six African countries; Ghana, Malawi, Nigeria, South Africa, Uganda and Zimbabwe.[22] The ACACIA project aims to determine the burden of asthma in school-going children by assessing asthma control in children between 12 and 14 years with symptoms compatible with asthma or a doctor-diagnosis of asthma to assess symptom severity and asthma control. As a separate study, we conducted focus group discussions (FGDs) with children with an asthma diagnosis or symptoms suggestive of asthma and their caregivers to obtain their perspectives on the barriers to managing asthma. The study plan, procedures and methods were standardised across all countries.[22]

For peer review only - http://bmjopen.bmj.com/site/about/guidelines.xhtml

Study population

Children and their caregivers were recruited initially as part of the ACACIA dataset. A Breathing Survey developed from questions in the Global Asthma Network (GAN) questionnaire (Supplementary file 1) was used to identify participants between 12 and 14 years with asthma symptoms or an asthma diagnosis.[22] Focus group participants (both children and their caregivers) were recruited using purposive sampling and categorised into four groups; children with a doctor-diagnosis of asthma (CAD) and their parents/caregivers (PAD), children with severe wheezing symptoms without a doctor-diagnosis of asthma (CND) and their parents/caregivers (PND), with FGDs held separately for each category. The aim of this group categorisation was to ensure views of both children and caregivers, as well as those with and without a formal asthma diagnosis, were adequately sampled.

Study setting

The FGDs were held between 1 November 2020 and 30 June 2021 in schools in cities across countries: Blantyre, (Malawi); Lagos, (Nigeria); Durban, (South Africa); Kampala, (Uganda); and Harare, (Zimbabwe). . Data from Ghana were not included for analysis due to challenges with COVID-19 restrictions in that country that impacted data collection. The in-country team of researchers and fieldworkers facilitated the focus group discussions.

Data collection

Focus groups were conducted face-to-face, with adherence to in-country-specific COVID-19 regulations with at least 1-2 FGD/s per category per country. All FGDs were moderated by at least two facilitators, one of whom was a study investigator at each site. FGDs took place in either English and /or isiZulu (SA), Shona (Zimbabwe) and Chichewa (Malawi). Other centres only used English. Focus groups were audio-recorded, transcribed verbatim and translated to English if needed in the country. Discussions lasted between 40-60 minutes and followed a predefined question guide specific to each focus group category. The discussion guides were developed after multiple iterations by the ACACIA core study team and informed by the study aims and literature review on barriers to asthma care. (Supplementary file 2)

Data analysis

The transcripts taken during the discussion were used to analyse the data using the NVivo 1.6 software program. We used an inductive analysis to examine the data.[23] Ten members of the ACACIA research group from all the participating countries independently developed codes

For peer review only - http://bmjopen.bmj.com/site/about/guidelines.xhtml

from the transcripts and then discussed the coding to develop a code book.[23,24] At least two investigators reviewed each transcript. A reiterative process was followed to apply the codebook to the transcribed data and determine whether further codes emerged. When no new codes emerged, the codebook was assumed to be a valid representation of the data and finalised (Supplementary 3).

Braun and Clarke's six-step thematic analysis was then used as the analytic framework to identify the patterns and themes within the coded data.[25] Major themes and subthemes were identified based on those commonly occurring codes in all countries. These sub-themes were highlighted and grouped into major themes based on commonly occurring patterns and relationships noted between the identified codes. All investigators reached consensus on these major themes and grouping of sub-themes after multiple iterations. Quotes chosen to illustrate the themes derived from the data were specifically selected only if they resonated across all countries.

Patient and Public Involvement

The focus groups were part of the wider ACACIA study and its patient and public involvement .[22.] The focus groups set up, especially for children, was developed at each site with the local school staff, to provide a friendly and safe experience in a familiar environment. We tested the questions used in the focus group discussion at one of our sites (Nigeria) before other sites. Patients were involved at several stages of the ACACIA study, but not directly for the recruitment and conduct of the focus group element. A summary of all ACACIA study results will be disseminated to the participating schools, who will communicate it to students and parents.

ETHICS

Ethical approval was granted at all the study sites: Malawi, (name deleted to maintain the integrity of the review process); Nigeria, (name deleted to maintain the integrity of the review process); Uganda, (name deleted to maintain the integrity of the review process); Zimbabwe, (name deleted to maintain the integrity of the review process); South Africa, (name deleted to maintain the integrity of the review process). Participants provided written informed consent as well as informed caregiver (parent /guardian) consent and child assent in accordance with both country/local regulations and United Kingdom (UK) ethics regulations. Trial registration

For peer review only - http://bmjopen.bmj.com/site/about/guidelines.xhtml

number (name deleted to maintain the integrity of the review process). Data were de-identified and stored on a password-protected computer with access only to researchers.

RESULTS

Description of the focus groups conducted

The data from 39 transcribed FGDs reflecting 256 participants across five countries were analysed. Table 1 indicates the breakdown of these groups per category and country. A total of 136 children were included with CAD 45.6% (n=62) and CND 54.4% (n=74). Of the 120 caregivers included they included PAD 42.5% (n=51) and PND 57.5% (n=69)

Table 1: Summary of children with asthma or asthma symptoms and their caregivers who participated in focus group discussions (n=256).

For peer review only

For peer review only - http://bmjopen.bmj.com/site/about/guidelines.xhtml

Summary of findings

Table 2 lists the subthemes and themes identified and Figure 1 shows the relationships. There were four major themes identified which were grouped into: (1) Poor understanding of Asthma, (2) Difficulties experienced with being diagnosed with Asthma, (3) Challenges with caring for children experiencing an acute asthma episode and (4) Sub-optimal uptake and use of prescribed asthma medicines .

Table 2. Major themes and subthemes identified.

Figure 1 .Relationships of the major themes and subthemes identified.

Poor understanding of asthma

This theme related to perspectives on how caregivers and children defined asthma and what was considered the cause of asthma. We could not ascertain differences in knowledge amongst caregivers of those with and without a diagnosis of asthma. We did find a distinction in how caregivers and children understood the causation of asthma. Children expressed fewer opinions on the familial or genetic nature of asthma than their caregivers. In all groups, there was a poor understanding of how environmental triggers and other factors such as prematurity and genetic predisposition contribute to the development of asthma.

> *There is dirt of some kind that interrupts ones breathing in their lungs so when air is meant to come in then you'll find that it's this dirt that is blocking the inhalation and exhalation of air.* (PND ZA, Group 6)

Difficulties experienced with being diagnosed with asthma

This theme related to perspectives being expressed about delays in being diagnosed, reflected predominantly in the groups with a formal doctor-diagnosis. Specific concerns related to a perceived reluctance of health care workers in making an asthma diagnosis with resulting frustration these delays cause.

> *I think that symptoms start with cough and flu. We waste a lot of time and yet the actual disease is not treated. So, after you have given that dose of cough and flu, when you go back, the doctor will tell you that is an infection yet it is asthma* (PAD, UG, and Group 2)

For peer review only - http://bmjopen.bmj.com/site/about/guidelines.xhtml

In addition, this theme reflected on the emotional distress felt with disruptions to lifestyles due to acute asthmatic episodes. Both children and caregivers indicated displeasure about the disruption in activities of normal daily life after being diagnosed with asthma. These included disruptions to school attendance, participation in sports and the child's share of household chores. Children expressed emotions of sadness and loss whilst caregivers expressed anxiety related to the disruption of their previous activities.

> *When am in an attack I can't do anything, I can't study, I can't go to class, I can't breathe properly, I can't focus, I can't understand. I can't be at school.* (CAD, MW, Group1)

> *Since we discovered she has asthma, my daughter is not exposed to a lot of things anymore. We don't allow her to sweep except if she's covered with a face mask before sweeping, we stopped her from drinking cold water and her room is cleaned properly, no rug, no cockroaches in the house, the fan is kept clean.* (PAD, NG, Group2)

Children also specifically expressed concerns about the stigma and discrimination they endured when their asthma diagnosis was known in the school environment. .These reactions from significant others impacted negatively on children and caregivers leading to feelings of isolation and this promoted non-disclosure

> *It's also embarrassing because they all look at you funny and some start excluding you from games because they fear that you will have an attack. Some end up avoiding you forever and you remain with no one to play with.* (CAD, ZW, Group1)

> *I don't tell people that I am asthmatic because they will start looking at me all different.* (CAD, UG, Group2)

Challenges with caring for children experiencing an acute asthma episode

In all groups, identifying and avoiding various asthma triggers was seen as an important component in managing symptoms.

For peer review only - http://bmjopen.bmj.com/site/about/guidelines.xhtml

Simple solutions like make sure your surroundings are clean, check for dampness, in your bathroom or the ceilings and your walls … there are lots of things you can do to ensure that, the triggers are reduced. (PND, ZA, Group2)

For caregivers, these avoidance strategies translated to altering the external environments of their children, whilst children focused more on how lifestyle changes impacted their daily lives. All groups articulated that they had difficulties with understanding what the various environmental triggers are and how to avoid them effectively.

I was outside and then my sister started sweeping the compound raising dust, the dust affected me and I started wheezing. I was then told to go inside the house and sleep. (CAD, ZW, Group1)

The theme also includes caregivers' perspectives on their interactions the health systems, with challenges in accessing acute care when a child experiences an acute asthma episode
In these interactions, caregivers reflected on their frustrations, financial dependencies and vulnerabilities. These arose following the experiences of limited and often difficult access to health care during an acute asthmatic attack.

Government hospitals are useless … It is possible that the doctor's house is very near but for him to come is a challenge. Sometimes they will leave in the middle of their shift and you come with your child to the hospital and they tell you to wait … meanwhile your child's condition is worsening (PAD, MW, and Group 1)

Apart from the hospital, I would also seek help from more accessible nurses in the neighbourhood or at home because these attacks usually happen when the hospital is too far and, in that moment, they may not be able to help you. (PND, UG, Group1)

Sub-optimal uptake and use of prescribed asthma medicines

Across all groups, common perspectives on the role, availability and acceptance of various types of medications to treat asthma were identified. All groups reflected on relatively easier

For peer review only - http://bmjopen.bmj.com/site/about/guidelines.xhtml

access to and acceptance of alternate and complementary asthma treatments than prescribed asthma medications.

> *Others told me to find a chameleon and leave it on flour for it to walk over, then to use the flour to make porridge to give the child. None of those things really worked! I have taken too much of these traditional medications!* (PAD, MW, Group1)

> *I also use the inhaling smoke method. My mother does the same thing of putting marijuana seeds on hot coal and covers me with a blanket to inhale the smoke but it will be for five minutes tops and it clears my chest and I breathe better.* (CND, ZW, Group1)

Juxtaposed to the acceptance of various alternate medications and treatment, there are widely held views that using and relying on inhalers should be met with caution. In addition, caregivers and children with asthma and no asthma diagnosis expressed various fears and myths about using prescribed inhalers. Concerns over possible addiction developing with the use of inhalers and fears of what could happen when inhalers are unavailable were expressed. In addition to these concerns, there is a clearly articulated preference for oral over inhaled medication by many.

> *Okay a lot of people would tell us …we can use the inhalers but when I grow up it will build up in my body … also what if I get addicted to the inhaler and one day I have shortness of breath and don't have the inhaler? What will happen? (*CAD, MW, group2)

> *The people from my village saw that I am using an inhaler, they asked me why I have started using it as if I don't know how such things end. But since last year when I started using the inhaler, everything is fine and we have not been admitted in the hospital since. I really had that mind-set that inhalers kill but since I started using them I saw that they are really helpful (*PAD, MW, and group1)

Barriers identified across countries

From the themes, we identified several barriers to asthma care across the five African countries, Table 3. Although there was some understanding of the role of genetic predisposition and

For peer review only - http://bmjopen.bmj.com/site/about/guidelines.xhtml

allergen triggers, children and caregivers still expressed the need for more information, specifically regarding trigger avoidance. This included lifestyle changes that had to be made for families and disruption of normal family routines and chores. Reliance on the use of oral and complementary medications was expressed with the stigma around the use of asthma inhaler therapies.

Table 3: Identified Barriers to asthma care across African countries

Discussion

Main findings

Several barriers to asthma care that were common in all five African countries in this study were identified .The relationships of these barriers to optimal asthma care in Africa is highlighted specifically from the participants' perspectives. On further exploration, several cross-cutting recommendations were extrapolated from the analysis of these relationships of the barriers to caring for children with asthma and these were derived from the author's perspectives.

Inadequate understanding of environmental triggers and genetic predisposition in causing asthma

Asthma is a complex heterogeneous condition with multiple risk factors. The role of genetics, atopy and environment is complex even for experienced researchers. It is therefore not surprising that there was a poor societal understanding of the differences between cure, prevention and control, including confusion between trigger avoidance and symptom control using chronic treatments. Studies from SSA have noted that caregivers and children ascribe triggers with causation[10, 17]. Thus, removing these 'offenders' equates to treatment/cure, lessening the perceived necessity for measures to prevent or control asthma.[10, 17]. These perspectives are identified in our study's findings of an inadequate understanding of environmental triggers, such as dust and cold weather. We speculate that this inadequate understanding translates to low 'necessity beliefs' towards the administration of chronic medications documented in Africa.[17] We thus further suggest that asthma education programs should clarify the role of triggers in the precipitation of asthma episodes and the need for medications for asthma control.

For peer review only - http://bmjopen.bmj.com/site/about/guidelines.xhtml

Hesitancy in health care workers making an asthma diagnosis resulting in delays and frustration

The delays in children receiving a doctor-diagnosis of asthma identified in our study can possibly be viewed within the context of poor symptom recognition, episodic management and low level of implementation of existing asthma guidelines by health care workers especially noted at the primary health care level (PHC) in Africa.[17,26] A systematic review by Mphahlele RE et al. records low rates of asthma diagnosis (38.8%), prescribed medication (47.6%) and an increasing prevalence of severe uncontrolled asthma in African children.[6] Simplifying diagnostic algorithms for asthma to ensure ease of implementation of care in the African context should be thus viewed as a crucial need. We strongly advocate for this, based on our findings.

Difficulties experienced coping with adjustments in lifestyles

Children's perspectives on being self-conscious when using inhalers and thus appearing 'different' was an important theme in the present study and has been reported across both resource-developed and challenged countries.[13,19,26] The stigma associated with asthma and the subsequent reluctance to use inhalers in front of peers thus impacts self-management interventions deemed crucial to most asthma care programmes.[19] The use of child-generated strategies where peer interactions help break down social barriers has been suggested for asthma school education programmes.[27] It is relevant to African policy since asthma education programmes must compete with multiple health care messaging from common infectious diseases like human immunodeficiency virus, tuberculosis and malaria.[20] In this study, we identify peer acceptance as a major barrier in coping with asthma adjustments. We thus recommend that school-going children be sensitised to provide support, specifically with inhaler usage, to affected peers.

Another barrier identified was the difficulty delineating asthma triggers and the inability to alter their environment adequately. Studies from both sSA and elsewhere corroborate this finding, and the use of global checklists that can provide guidance on the range of triggers and viable environment modifications may be useful for inclusion, for example, in the World Health Organization Package of essential non-communicable disease interventions for primary health care (WHO PEN).[28]

Difficulties in obtaining hospital care when a child experiences an acute asthma episode

For peer review only - http://bmjopen.bmj.com/site/about/guidelines.xhtml

Poor access to asthma care across resource-constrained contexts, including Africa, has been noted as a general barrier, and our study corroborates this across all sampled countries.[7] This limited and often difficult access often leads to caregivers seeking alternate avenues for care and delaying appropriate care.[29,30] The WHO PEN attempts to improve asthma management at PHC sites; however, logistic challenges, including lack of essential medications at some sites, require interventions.[28] We specifically identify the need for access to oxygen, nebulisers and monitoring, especially during acute episodes. The concern is that while PHC services may be accessible geographically, the cost of transport, waiting times, and loss of income also contribute to poor access. We thus advocate for improved access to both acute and chronic asthma care in primary health care settings.

Easier access to and acceptance of alternate and complementary asthma treatments compared with prescribed asthma medication

The acceptance and usage of traditional medicines and alternative treatments for asthma identified in this study have been previously ascribed to negative perceptions of prescribed medications and seen as inappropriate health-seeking behaviour that contributes to severe asthma.[17,26,30] In the current study, although ambivalence to prescribed medications contributes to poor inhaler use, widespread access to traditional and complementary medicine and indigenous knowledge systems also contributed to alternative treatments. We propose that the propagation of key programmes that advocate for and educate on inhaler therapies and devices should actively engage traditional health systems in the African context to understand better and embed asthma education in these systems.

Preference for prescribed oral asthma medication with fears and myths over using metered dose inhalers

Multiple studies have corroborated the poor usage and non-adherence to metered dose inhalers in childhood asthma as a major barrier to care.[11,17] This is further seen with the low levels of inhaled corticosteroids (6.7- 17%) and 73% of doctor-diagnosed/suspected asthma not using any inhaler documented.[6,31] Over-reliance on short-acting beta agonist (SABA) inhalers per se is also a barrier to appropriate care.[32] A preference for oral over inhaler-administered medications and widely held concerns that metered dose inhalers were addictive and harmful identified have been noted previously.[10,18] These factors are identified as leading reasons for inadequate use of metered dose inhalers.

For peer review only - http://bmjopen.bmj.com/site/about/guidelines.xhtml

With efficacy and safety studies advocating the use of single rapid onset (long-acting beta-agonist/inhaled corticosteroids (LABA/ICS) metered dose inhalers for maintenance, and reliever therapy there is a potential that this single therapeutic option could transform asthma care in sSA.[5,11] Advocacy and policy interventions to make this therapeutic option cheaper need urgent action.[17,11] We suggest that universal access to using a single therapeutic metered dose inhaler, where appropriate, be strongly advocated for in Africa and, in addition, knowledge to dispel fears of using inhaler therapies and devices be included in asthma care plans.

Strengths and limitations of this study

This study presents a large qualitative dataset from multiple sites across Africa, reflecting a wide participant base. The juxtaposition of children with and without a formal diagnosis and their caregivers generated rich data and new insights. We actively sought to identify common barriers across all sites, thus generating data generalisable to the SSA context. Transcription verbatim and using qualitative software to code the data enhanced the reliability. We attempted to ensure a fair and equitable coding team to decrease in-country biases, and this study thus reflects one of the few unique cross-country analyses from Africa.

Some loss of meaning may have occurred during the translation of African language quotes however, the data were analysed in the source language by members of the large international coding team who checked the semantic and context equivalence for translated quotes. We did not conduct member checks. Basic demographic data on participants was not provided except for enabling categorisation into the various groups.

We used an inductive approach with constant data comparisons and discussions to inform our understanding of the views expressed. We were aware of the risk of researcher bias in qualitative research and actively discussed reflexivity in the regular multidisciplinary team discussions, which aided analysis and informed interpretation. In addition, we have provided an explicit account for the derivation of the recommendations.

Conclusion

In a continent with rapidly increasing levels of severe uncontrolled asthma, multiple barriers to asthma diagnosis and management are identified along the entire trajectory of care. Exploration of these barriers reveals several broad, generalisable recommendations to modify asthma care plans that can mitigate challenges and potentially transform asthma care in Africa. Advocacy and tailored education are needed to promote access and acceptance for metered

For peer review only - http://bmjopen.bmj.com/site/about/guidelines.xhtml

dose inhalers, including advocating for access to a single therapeutic, preventative and treatment modality. Healthcare systems require simpler diagnostic pathways and easier emergency access for childhood asthma across the continent.

DECLARATIONS

Acknowledgements

The authors wish to express our gratitude to all the children , caregivers and patient advisers who participated in this study.

Funding

This research was funded by the National Institute for Health Research (NIHR) , project number 17/63/38269211, trial registration number: 269211

Availability of data and materials

Anonymised data is available from the corresponding author upon request.

Authors' contributions

RM, JG, HAM, GM, and IT contributed to the study's conception. KLN, REM, SN, AS, MM, YG, YK, RN and OA performed the data collection, checking and coding the transcripts. KLN, REM, SN, AS, MM, YG, YK, RN and OA contributed to data analysis and interpretation. KLN, REM, SN and RM drafted the manuscript, and all authors provided critical revisions and editing. All authors reviewed the manuscript. RM and JG accept full responsibility for the work and the conduct of the study, had access to the data, and controlled the decision to publish

Disclaimer

The views and opinions expressed in this article are those of the authors and do not necessarily reflect the official policy or position of any affiliated agency of the authors.

Ethical consideration and consent to participate

This is contained in the manuscript.

Consent for publication

Not applicable.

Competing interests

JG reports personal fees from GSK, personal fees from Vifor Pharmaceuticals, personal fees from Novartis, personal fees from BV Pharma, personal fees from AstraZeneca, outside the submitted work.

References

For peer review only - http://bmjopen.bmj.com/site/about/guidelines.xhtml

1. Global Asthma Network. The Global Asthma Report 2018. http://globalasthmanetwork.org/Global%20Asthma%20Report%202018.pdf (accessed 03 October 2022)
2. Mortimer K, Reddel HK, Pitrez PM, et al. Asthma management in low and middle income countries: case for change. *Eur Respir J.* 2022; 60(3):2103179. doi: 10.1183/13993003.03179-2021.
3. Adeloye D, Chan KY, Rudan I, Campbell H. An estimate of asthma prevalence in Africa: a systematic analysis. *Croat Med J.* 2013; 54(6):519-31. doi: 10.3325/cmj.2013.54.519.
4. Asher MI, Rutter CE, Bissell K, et al.; Global Asthma Network Phase I Study Group. Worldwide trends in the burden of asthma symptoms in school-aged children: Global Asthma Network Phase I cross-sectional study. *Lancet* 2021; 398(10311):1569-1580. doi: 10.1016/S0140-6736(21)01450-1.
5. Global Initiative for Asthma. Global Strategy for Asthma Management and Prevention, 2022: GINA 2022. https://ginasthma.org/wp-content/uploads/2022/07/GINA-Main-Report-2022-FINAL-22-07-01-WMS.pdf (accessed 03 October 2022)
6. Mphahlele RE, Kitchin O, Masekela R. Barriers and determinants of asthma control in children and children in Africa: a systematic review. *BMJ Open* 2021;11:e053100. doi: 10.1136/bmjopen-2021-053100.
7. Meghji J, Mortimer K, Agusti A, et al. Improving lung health in low-income and middle-income countries: from challenges to solutions. *Lancet* 2021 Mar 6; 397(10277):928-940. doi: 10.1016/S0140-6736(21)00458-X.
8. Nagel G, Weinmayr G, Kleiner A, et al.; ISAAC Phase Two Study Group. Effect of diet on asthma and allergic sensitisation in the International Study on Allergies and Asthma in Childhood (ISAAC) Phase Two. *Thorax* 2010; 65(6):516-522. doi: 10.1136/thx.2009.128256.
9. Rodriguez A, Vaca MG, Chico ME, et al. Rural to urban migration is associated with increased prevalence of childhood wheeze in a Latin-American City. *BMJ Open Respir Res* 2017; 4:e000205.
10. Mpairwe H, Tumwesige P, Namutebi M, et al. Asthma control and management among schoolchildren in urban Uganda: results from a cross-sectional study. *Wellcome Open Res* 2019; 4:168. doi: 10.12688/wellcomeopenres.15460.1.
11. Masekela R, Mortimer K, Nantanda R, Lesosky M, Meme H, Devereux G, et al. Asthma care in sub-Saharan Africa: Mind the gap! *J Pan Afr Thorac Soc* 2022; 3(2):59-62. doi:10.25259/JPATS_12_2022

For peer review only - http://bmjopen.bmj.com/site/about/guidelines.xhtml

12. García-Marcos L, Asher MI, Pearce N, et al.; Global Asthma Network Phase I Study Group. The burden of asthma, hay fever and eczema in children in 25 countries: GAN Phase I study. *Eur Respir J* 2022; 60(3):2102866. doi: 10.1183/13993003.02866-2021.

13. Callery P, Milnes L, Verduyn C, Couriel J. Qualitative study of young people's and parents' beliefs about childhood asthma. *Br J Gen Pract* 2003; 53(488):185-90. https://bjgp.org/content/bjgp/53/488/185.full.pdf (accessed 03 October 2022)

14. Lakhanpaul M, Culley L, Robertson N, et al. A qualitative study to identify parents' perceptions of and barriers to asthma management in children from South Asian and White British families. *BMC Pulm Med* 2017; 17(1):126. doi: 10.1186/s12890-017-0464-9.

15. Chong YY, Leung D, Mak YW. When Control Exacerbates Distress: A Qualitative Study Exploring the Experiences of Hong Kong Chinese Parents in Caring for a Child with Asthma. *Int J Environ Res Public Health* 2018; 15(7):1372. doi: 10.3390/ijerph15071372.

16. Chen SH, Huang JL, Yeh KW, Tsai YF. The Stress of Caring for Children with Asthma: A Qualitative Study of Primary Caregivers. *J Nurs Res* 2015;23(4):298-307. doi: 10.1097/JNR.0000000000000083.

17. Kassa E, Kebede RA, Habte BM. Perceptions towards childhood asthma and barriers to its management among patients, caregivers and healthcare providers: a qualitative study from Ethiopia. *BMC Pulm Med* 2022; 22(1):184. doi: 10.1186/s12890-022-01984-2.

18. Barakat D, Rockers PC, Vian T, et al. Access to asthma medicines at the household level in eight counties of Kenya. *Int J Tuberc Lung Dis* 2018; 22(5):585-590. doi: 10.5588/ijtld.17.0664.

19. Rose S, Paul C, Boyes A, et al. Stigma-related experiences in non-communicable respiratory diseases: A systematic review. *Chron Respir Dis* 2017; 14(3):199-216. doi: 10.1177/1479972316680847.

20. Penza-Clyve SM, Mansell C, McQuaid EL. Why don't children take their asthma medications? A qualitative analysis of children's perspectives on adherence. *J Asthma* 2004; 41(2):189-97. doi: 10.1081/jas-120026076.

21. Tefera BB, Kibret MA, Molla YB, et al. The interaction of healthcare service quality and community-based health insurance in Ethiopia. *PLoS One* 2021; 16(8):e0256132. doi: 10.1371/journal.pone.0256132.

22. Mosler G, Oyenuga V, Addo-Yobo E, et al. Achieving Control of Asthma in Children in Africa (ACACIA): protocol of an observational study of children's lung health in six sub-

For peer review only

For peer review only - http://bmjopen.bmj.com/site/about/guidelines.xhtml

Saharan African countries. *BMJ Open* 2020; 10:e035885. doi:10.1136/ bmjopen-2019-035885

23. Roberts K, Dowell A, Nie JB. Attempting rigour and replicability in thematic analysis of qualitative research data; a case study of codebook development. *BMC Med Res Methodol* 2019; 19(1):66. doi: 10.1186/s12874-019-0707-y.
24. Boyatzis RE. Transforming qualitative information: thematic analysis and code development. Thousand oaks CA: SAGE Publications Inc; 1998. ISBN-13: 978-0761909613.
25. Braun V, Clarke V. Using Thematic Analysis in Psychology. Qualitative Research in Psychology 2006; 3:77-101. doi: 10.1191/1478088706qp063oa.
26. Lion-Cachet HC, Musonda JMM, Omole OB. Severe asthma in South Africa: A literature review and management approach for primary care. *S Afr Fam Pract* 2021; 63(1):e1-e10. Doi: 10.4102/safp.v63i1.5179.
27. Kelada L, Molloy CJ, Hibbert P, et al. Child and caregiver experiences and perceptions of asthma self-management. *NPJ Prim Care Respir Med* 2021; 31(1):42. doi: 10.1038/s41533-021-00253-9.
28. World Health Organization. WHO package of essential non-communicable (PEN) disease interventions for primary health care. 07 September 2022. https://apps.who.int/iris/rest/bitstreams/1301957/retrieve (accessed 04 October 2022)
29. Searle A, Jago R, Henderson J, et al. Children's, parents' and health professionals' views on the management of childhood asthma: a qualitative study. *NPJ Prim Care Respir Med* 2017; 27(1):53. doi: 10.1038/s41533-017-0053-7.
30. Joseph CL, Williams LK, Ownby DR, et al. Applying epidemiologic concepts of primary, secondary, and tertiary prevention to the elimination of racial disparities in asthma. *J Allergy Clin Immunol* 2006; 117(2):233-40; quiz 241-2. doi: 10.1016/j.jaci.2005.11.004.
31. Mphahlele REM, Lesosky M, Masekela R. The prevalence, severity, and risk factors of asthma in South African children. Paper presented at The European Respiratory Society International Congress; PA3953; 2022 Sep 4-6; Barcelona, Spain.
32. Zeitouni MO, Al-Moamary MS, Coussa ML, Riachy M, Mahboub B, AlHuraish F, Zidan MH, Metwally MM, Aksu K, Yavuz E, Kalla IS, Chakaya J, Abdelmadjid S, Ghedira H. Challenges and recommendations for the management of asthma in the Middle East and Africa. *Ann Thorac Med* 2022; 17(2):71-80. doi: 10.4103/atm.atm_469_21.

For peer review only - http://bmjopen.bmj.com/site/about/guidelines.xhtml

For peer review only - http://bmjopen.bmj.com/site/about/guidelines.xhtml

Table 1: Summary of children with asthma or asthma symptoms and their caregivers who participated in focus group discussions (n=256).

**Country (n=total number of focus groups)**	**Children n=136**				**Caregivers n=120**			
	Children with a doctor-diagnosis of Asthma (CAD)		Children with severe asthma symptoms but no diagnosis (CND)		Caregivers of children with a doctor-diagnosis of asthma (PAD)		Caregivers of children with severe asthma symptoms but no diagnosis. (PND)	
	Focus group (n)	Number of participants	Focus group (n)	Number of participants	Focus group (n)	Number of participants	Focus group (n)	Number of participants
**Malawi, MW (n=4)**	2	8	1	8	1	6	0	0
**Nigeria, NG(n=7)**	2	14	2	12	2	12	1	8
**South Africa, ZA (n=15)**	3	19	3	29	3	21	6	41
**Uganda, UG(n=7)**	2	12	1	6	2	12	2	19
**Zimbabwe, ZW(n=6)**	1	9	3	19	0	0	2	10
**Total participants (n=256)**	**10**	**62**	**10**	**74**	**8**	**51**	**11**	**69**

For peer review only

For peer review only - http://bmjopen.bmj.com/site/about/guidelines.xhtml

Table 2: Major themes and subthemes and their relationships

**Major themes**	Poor understanding of Asthma	Difficulties experienced with being diagnosed with Asthma	Challenges with caring for children experiencing an acute asthma episode	Sub-optimal uptake and use of prescribed asthma medicines
**Subthemes**	• Confusion about asthma definitions<br>• Lack of clarity about what causes asthma	• Concerns about delays in getting an asthma diagnosis<br>• Disruptions in child's life on being diagnosed with asthma<br>• Negative responses from significant others to child's asthma diagnosis	• Confusion around triggers of an acute asthma episode<br>• Lack of knowledge on recognising and responding to an acute asthma episode<br>• Difficulties in access to hospital care when a child experiences an acute asthma episode	• Preferences for trying alternative/complementary treatments<br>• Poor acceptance with using inhalers

For peer review only - http://bmjopen.bmj.com/site/about/guidelines.xhtml

For peer review only

For peer review only - http://bmjopen.bmj.com/site/about/guidelines.xhtml

675 **Table 3: Identified Barriers to asthma care across African countries**

676

Barriers to asthma care across the African countries
1. Inadequate understanding of environmental triggers and causes of asthma impact on optimal, holistic asthma care<br>2. Reluctance in health care workers to make an asthma diagnosis results in delays in diagnosing asthma<br>3. Difficulties experienced coping with adjustments in lifestyles required with asthma hamper prevention of acute episodes<br>4. Limited access to health care during an acute asthmatic episode<br>5. Preferred usage of alternate and complementary asthma treatments delays uptake of prescribed asthma medication<br>6. Preferences in using oral asthma medication delays early adoption of inhaler medication<br>7. Fears and myths prevent uptake use of metered dose inhalers

677

678

679

For peer review only

For peer review only - http://bmjopen.bmj.com/site/about/guidelines.xhtml

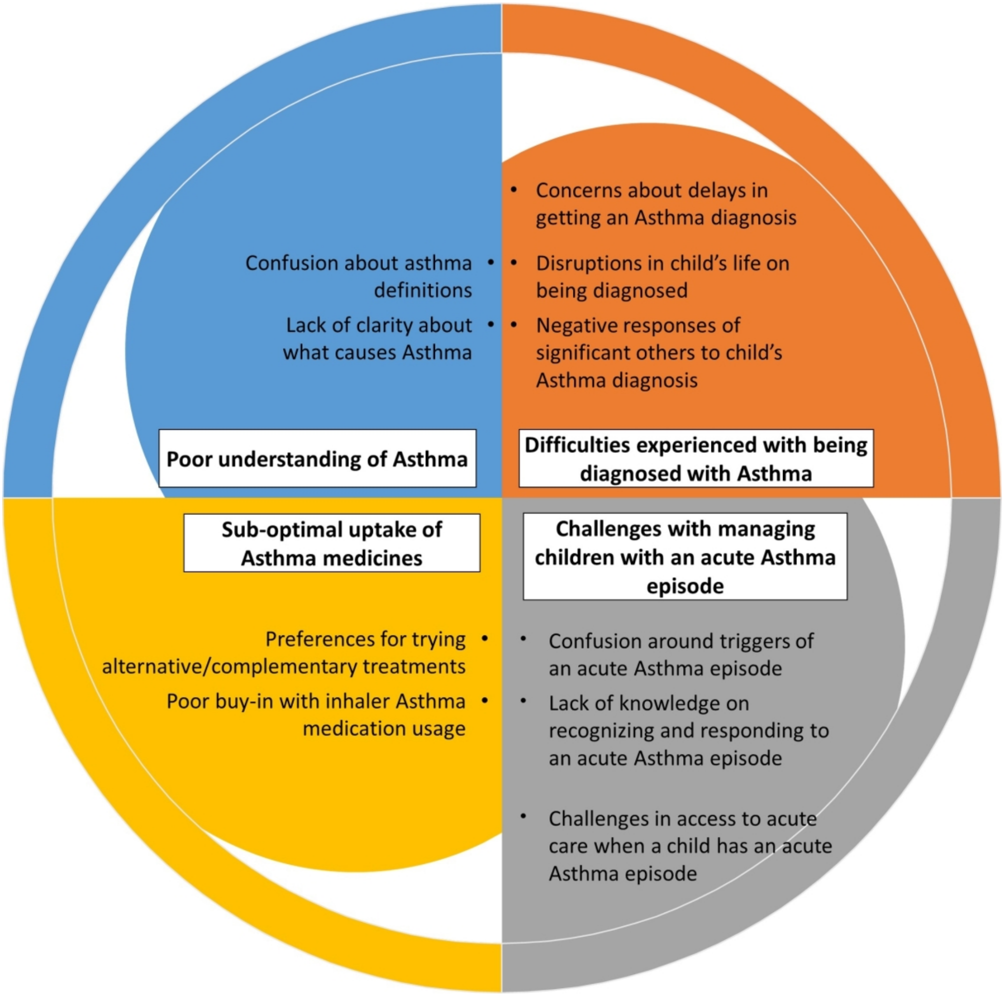

Figure 1 Relationship of the Major themes and sub-themes identified.

176x174mm (300 x 300 DPI)

acacia achieving control of asthma in children in africa

UNIVERSITY OF KWAZULU-NATAL INYUVESI YAKWAZULU-NATALI

ACACIA Breathing Survey

QR code label or Participant ID:

____

Introduction

We are a group of researchers interested in healthy breathing. In the questions below we will ask if you sometimes make a noise when you breathe, which is called wheezing. Wheezing is a whistling sound from the chest that some people make when they breathe.

Section 1: Personal Details

1. **What is your name?** ____

2. **Today's date:** DAY / MONTH / YEAR

3. **School:** ____

4. **Are you male or female?**

   ☐ Male

   ☐ Female

5. **How old are you?** __________

6. **What is your date of birth?** DAY / MONTH / YEAR

7. **Which school class or school year are you in?** ______________________________

8. **How would you describe your ethnicity?**

   ☐ Black

   ☐ White

   ☐ East Asian, such as Chinese, Japanese, or Korean

   ☐ South Asian, such as Indian, or Bangladeshi

   ☐ Mixed race, please specify: ________________________

   ☐ Other, please specify: ____________________________

For peer review only

Section 2: Breathing

1. **Have you had wheezing or whistling in the chest in the past 12 months?**

☐ Yes

☐ No

IF YOU HAVE ANSWERED "NO" PLEASE SKIP TO QUESTION 5

2. **How many attacks of wheezing have you had in the past 12 months?**

☐ None

☐ 1 to 3

☐ 4 to 12

☐ More than 12

3. **In the past 12 months, how often, on average, has your sleep been disturbed due to wheezing?**

☐ Never woken with wheezing

☐ Less than one night per week

☐ One or more nights per week

4. **In the past 12 months, has wheezing ever been severe enough to limit your speech to only one or two words at a time between breaths?**

☐ Yes

☐ No

5. **Have you ever had asthma?**

☐ Yes

☐ No

IF YOU HAVE ANSWERED YES,

6. **Was your asthma confirmed by a doctor?**

☐ Yes

☐ No

For peer review only - http://bmjopen.bmj.com/site/about/guidelines.xhtml

acacia achieving control of asthma in children in africa

Page | 1

FGD 1: Children (12-14 yrs) with severe asthma and diagnosis of asthma

Before the discussion

1. Introduction
2. Game/warming up exercise

Discussion

1. Starter activity: Go around the room, everyone should finish the sentence: 'Asthma is…

   (The facilitators start, e.g. Asthma is… not easy/ hard to talk about/ limiting/ not a problem…)

2. How much do breathing problems affect what you do?
   - … At school?
   - … At home?
   - … Anywhere else?
3. If you have asthma medication, how do you take your asthma medications?
   - What helps you take your asthma medication?
   - What makes it difficult to take your asthma medication?
   - What do you dislike about your asthma medication?
   - Is there any other medication that you think helps you? For example traditional medication?
   - What are some of the ideas you have to make it easier to take your asthma medication?
4. How do you feel if you have difficulties breathing?
   - … When you are around your friends?
     - How do they react?
   - … When you are in class?
     - How does the teacher react?
5. *What do you believe people around you think about your asthma?*
   - Friends,
   - family,
   - teachers
   - What do you tell people about your asthma? And who do you talk to?

For peer review only - http://bmjopen.bmj.com/site/about/guidelines.xhtml

acacia achieving control of asthma in children in africa

For peer review only

NIHR | National Institute for Health Research

Queen Mary University of London

For peer review only - http://bmjopen.bmj.com/site/about/guidelines.xhtml

FGD 2: Children (12-14yrs) with severe asthma and no diagnosis

Before the discussion:

1. Introduction
2. Game/warming up exercise

Discussion:

1. What would you do if you have difficulties breathing?
   - Can you think of anything that might make it easier to breathe?
   - Do you think there is anything that might make it worse?
2. Have you ever taken any medication or remedies for your breathing difficulty?
   - Tell me about it.
3. How much do breathing problems affect what you do?
   - … At school?
   - … At home?
   - … Anywhere else?
4. How do you feel if you have difficulties breathing...?
   - … When you are around your friends?
     - How do you think they would react?
   - … When you are in class?
     - How has the teacher reacted?
5. Have you heard of asthma?
   - What do you think does it mean to have asthma?
6. What would you think if a very good friend suddenly tells you that he/she has asthma?

For peer review only

For peer review only - http://bmjopen.bmj.com/site/about/guidelines.xhtml

Page | 4

FGD 3: Parents of children with diagnosed asthma

Before the discussion

1. Introduction
2. Warm up exercise / tea & cookies
3. Short questionnaire
   - Knowledge questions (symptoms of asthma/triggers etc.)
   - Questions about beliefs around asthma (e.g. How much support do you think a person with asthma needs? A lot,..., none)
   - Where do you go to seek help?

Discussion

1. Asthma is... (finish sentence)
2. Tell us about your experience with asthma?
   - Tell us about your experience with healthcare
     - diagnosis
   - ... Experience with asthma treatment (inhalers, alternative treatment methods etc.)
   - Tell us about any impact the asthma diagnosis had for you and your family
     - Financially
     - Socially
3. Can you tell us what makes it difficult for your child to take the asthma medication?
4. What do you think it means for your child to have asthma?
   - Would you have any special concerns about your child because of their asthma?
     - Would you think there is anything that an asthmatic child should avoid?
5. Do you have any examples of what makes life with asthma difficult for your child?
6. Is there anything that helps your child to live with asthma, apart from medication?

FGD 4: Parents of children without asthma

Before the discussion

1. Introduction
2. Warm up exercise/ tea & cookies
3. Short questionnaire
   - Knowledge questions (symptoms of asthma/triggers etc.)

For peer review only - http://bmjopen.bmj.com/site/about/guidelines.xhtml

- Questions about beliefs around asthma (e.g. How much support do you think a person with asthma needs? A lot,…, none)
- If your child had problems to breathe sometimes, where would you go to seek help?

Discussion

1. How would you describe asthma?
    - Tell us about your experience with asthma (Do you know anyone with asthma?)
    - If you hear that your child's friend had asthma, what would you say to your child?
2. If you are told that your child has asthma,
    - How would you react?
    - What would you tell your child?
    - Where would you seek help?
    - How would you manage?
    - How would you feel?
3. Why do you think someone might be worried about asthma?
    - Who do you think might be affected by asthma?

For peer review only

For peer review only - http://bmjopen.bmj.com/site/about/guidelines.xhtml

FGD 1: Children (12-14 yrs.) with severe asthma and diagnosis of asthma		
**Code**	**Definition**	**Purpose/meaning of code**
1. Definition of asthma	Participant defines asthma	Indicates participant's knowledge of what asthma is<br>Indicates Knowledge
2. Effects of breathing difficulties	Participant describes their own experience of how their asthma affects them when they are in various environments such as school, home and anywhere else	Participant indicates how much their breathing problems alter their activities in different environments.
3. Positive experience with asthma medication	participants describe what is pleasing to them about their medications and why they use them	participant describe what improves their symptoms<br>what makes it easy for them to use the medications
4. Negative experience with asthma medication	Participant describe what is not pleasing about their medication and why they do not like to use them.	What make is difficult to take their medication, can be taste, smell, stigma attached to taking medication in public
5. Alternative medication	Participant indicates and describes an experience of taking any alternative medication for their asthma	Indicate alternative medication/methods or idea to relieve their symptoms. Can be at school, home or anywhere else
6. reaction of friends to an asthma attack	How their friends have reacted to them while having an asthma attack and how they felt about their friend's reaction	Determine if there are perceived stigma limiting expression of symptoms. Determine the extent to which peers could provide support or otherwise
7. Reaction to having an asthmatic attack teachers	Participant recalls or describes how their teachers reacted when they were having an asthma attack and how they felt when that was happening	How they perceive getting help from the teachers. Finding out the aid that is offered to the learner and how teachers respond/react to asthmatic learner
8. Perception of asthma from friends	Describe whether friends are aware that they have asthma or not? Describe how the friends feel about having an asthma sufferer as a friend	Describe friends' reaction to them having asthma.<br>how has the relationship being impacted
9. Perception of asthma from family	Participants describe what they family think or feel about their asthma	Are they treated differently in their family because of their asthma? How does their family react when they exhibit symptoms? Have there been any changes to the household since their diagnosis?
10. Perceptions of asthma from teachers	Participants describe what their teachers feel about them having asthma	Do their teachers know they have asthma? What kind of assistance do they receive from the teachers to reduce risk of developing symptoms in school?<br>What kind of assistance do they receive when they exhibit asthma symptoms?
11. Personal perceptions about your asthma	Participants describe how they feel about their own asthma, what are their experiences around it	We are trying to find out how they feel or think about their asthma, what are their experiences around it, good or bad

For peer review only

FGD 2: Children (12-14yrs) with severe asthma and no diagnosis		
**Code**	**Definition**	**Purpose/meaning of code**
1. Easing breathing difficulties	Participants describe what makes it easier for them to breath when they have trouble breathing	What are their coping mechanisms, what do they do in order for their symptoms not to get worse
2. Exacerbations of breathing difficulties	Participants describe what makes it worse for them when they have difficulties breathing	identify things that make their symptom worse
3. Medications	Participants indicate if they have taken any medications for their symptoms or any alternative methods to help them with their symptoms. Identify medication use : type	Finding out what medications and remedies they have used. Describe what is done to bring relief
4. Effects of experiencing breathing problem	Participant describes their experience of how their asthma affects them when they are in various environments such as school, home and anywhere else	description of experience of asthma symptoms- home, school etc.
5. Reaction from friends	Participant recalls and describe how their friends reacted when they were having an asthma attack and how they felt when that was happening	How they perceive getting help from their peers. Finding out they experience any stigma from exhibiting breathing difficulties in front of their friends
6. Reaction from teachers	Participant recalls and describe how their teachers reacted when they were having an asthma attack and how they felt when that was happening	How they perceive the help or otherwise from school. describe what was done and the teacher's reaction
7. Perceptions of having breathing problems /asthma	Participant perception of what asthma is and how they will react if a friend have asthma	Definition of asthma and perception of it

For peer review only

FGD 3: Parents of children with diagnosed asthma		
**Code**	**Definition**	**Purpose/meaning of code**
1.Definition of asthma	Participant defines asthma/ describe asthma symptoms	Indicates participant's knowledge of what asthma is
2. Response to initial asthma diagnosis	Participant describes their experience when their child was diagnosed with asthma	Describe parents' initial reaction to asthma diagnosis
3.Experience with healthcare with regards asthma		Journey to diagnosis of asthma in healthcare
4.Positive experiences asthma medication	Participants describes their experience with asthma treatment	describe parents knowledge about asthma treatment and support provided when child was diagnosed
5.Negative experience with asthma medication	Participant describes negative aspects of asthma medication. What they don't like about the medication	What make is difficult to for their children to take the medication, can be taste, smell, stigma attached to taking medication in public
6.Financial impact of asthma diagnosis	Cost of medication, availability of medication at their local healthcare facility, fear of side effects	determine the financial implication (direct and indirect cost of asthma care)
7.Social implications of the diagnosis	Social implications of the asthma diagnosis on their children and family	Try to find out the perceptions and attitude to asthma in the community they live in or perceptions from their family. Find out the if they have experienced stigma or support
8.Concerns around asthma	What it means for their child to have asthma. Their perceptions and worries around the diagnosis and care of asthma	How people would Perceive or treat their child, how the child would cope if not at home etc.
9.Enablers in asthma care of children	Participant describes what makes it easier for their child with an asthma diagnosis to be cared for	Parents perception of what will make life easier for a child with asthma
10. Difficulties of an asthma diagnosis	Participant describes what has been difficult since the asthma diagnosis	Parents perception of what has made life difficult for a child with asthma

For peer review only

FGD 4: Parents of children without asthma		
**Codes**	**Definition**	**Purpose/meaning code**
1. Knowledge of breathing problems in children	Participant recalls and describes their experience and knowledge around asthma	Try to find out their knowledge around asthma and if they know someone with asthma
2. Perception of asthma in other children	Their reactions if their child's friend had asthma	Try to find out their perception around asthma, fear, stigma etc.
3. Reaction if their child had asthma	Their reaction if their child was diagnosed with asthma	Try to find out their perception and feeling about asthma
4. communicating with child on asthma	How would they tell their child about the diagnosis	Try to find out their knowledge around asthma or what resources they would use to tell the child more about asthma. How would they communicate asthma information to their child
5. Seeking help if their child had asthma	Where would they go to seek help for asthma, asthma care, treatment and information	Try to find out if they have access to resources that can help them and their child. What type kind of resources are available to them in case of emergencies<br>Where they would seek help i.e. from people, places
6. Manage asthma care/Breathing problems	Participant describe what they would do differently if their child was diagnosed with asthma and how they would care for them	How would they manage the care of their child who has asthma- things they would do in terms of care
7. Positive Feelings on asthma	Their perceptions around asthma	any positive feeling described
8. Negative feelings /Concerns around asthma	Participant describes their concerns about asthma or an asthmatic's concern about asthma	Describes such feeling as fear, afraid of stigma, afraid of financial burden, burden of visiting hospital frequently, fear for sudden asthma attacks or what the child may not be able to do.

For peer review only

For peer review only

COREQ Qualitative research checklist :
Barriers to Childhood asthma care in Sub-Saharan Africa: a multi-country qualitative study with children and their caregivers.

No Item Guide	questions/description	
**Domain 1:**	**Research team and reflexivity**	NB We attempted to ensure a fair and equitable coding team to decrease in-country biases **Page 5, data analysis 2nd sentence**<br>We used an inductive approach with constant data comparisons and discussions to inform our understanding of the views expressed. We were aware of the risk of researcher bias in qualitative research and actively discussed reflexivity in the regular multidisciplinary team discussions, which aided analysis and informed interpretation. **Page 5 , data analysis 2nd paragraph**
**Personal Characteristics**		
1. Interviewer/facilitator?	Which author/s conducted the interview or focus group?	All focus group discussions (FGDs) were moderated by at least two facilitators, one of whom was a study investigator at each site. **Page 4 , Data collection**<br>Reference is provided for the protocol **Reference 22**
2. Credentials	What were the researcher's credentials? E.g. PhD, MD	"one of whom was a study investigator at each site', Research project has a list of credentials of all investigators- reference for the published protocol included<br>**Page 4 ,data collection 2nd sentence and full protocol reference 22**
3. Occupation	What was their occupation at the time of the study?	Research project has a list of credentials of all investigators- reference for the published protocol included<br>**Page 4 ,data collection 2nd sentence and full protocol reference 22**,
4. Gender	Was the researcher male or female?	All genders .multiple researchers<br>**Page 4**
5. Experience and training	What experience or training did the researcher have?	Research project has a list of credentials of all investigators- reference for the published protocol included<br>**Page 4 and Reference 22 Protocol has details of the greater research project**
**Relationship with participants**		
6. Relationship established Was a relationship established prior to study commencement?	Was a relationship established prior to study commencement?	The details of the larger study are provided. 'Children and their caregivers were recruited initially as part of the ACACIA dataset'<br>**Page 4 Study population and reference 22**

For peer review only

COREQ Qualitative research checklist :
Barriers to Childhood asthma care in Sub-Saharan Africa: a multi-country qualitative study with children and their caregivers.

7. Participant knowledge of the interviewer	What did the participants know about the researcher? e.g. personal goals, reasons for doing the	All moderators introduced themselves at all FGDs. The standardised interview guide included a formal introductions component<br>Participants provided written informed consent as well as informed caregiver (parent /guardian) consent and child assent in accordance with both country/local regulations and United Kingdom (UK) ethics regulations.- this process provided information on the goals of both the larger project and the FGDs<br>**Page 4 data collection**
8. Interviewer characteristics,	What characteristics were reported about the interviewer/facilitator? e.g. Bias, assumptions, reasons and interests in the research topic	There were multiple facilitators across all countries. Focus groups were conducted face-to-face, with adherence to in-country-specific COVID-19 regulations. All FGDs were moderated by at least two facilitators, one of whom was a study investigator at each site. **Page 4 , data collection paragraph**<br>Participants provided written informed consent as well as informed caregiver (parent /guardian) consent and child assent in accordance with both country/local regulations and United Kingdom (UK) ethics regulations.- this process provided information on the goals of both the larger project and the FGDs<br>**Page 5 , ethics paragraph**
**Domain 2: study design**		
**Theoretical framework**		
9. Methodological orientation and theory	What methodological orientation was stated to underpin the study? e.g. grounded theory, discourse analysis, ethnography, phenomenology, content analysis	This study was across 6 countries and used an inductive analysis to examine the data.[23] The methodological orientation was largely content analysis using a codebook analysis due to the large number of transcripts .We have provided references for the process we followed with the code book development<br>Ten members of the ACACIA research group from all the participating countries independently developed codes from the transcripts and then discussed the coding to develop a code book.[23,24] At least two investigators reviewed each transcript. A reiterative process was followed to apply the codebook to the transcribed data and determine whether further codes emerged. When no new codes emerged, the codebook was assumed to be a valid representation of the data and finalised<br>**Page 5 , data analysis**

For peer review only - http://bmjopen.bmj.com/site/about/guidelines.xhtml

COREQ Qualitative research checklist :
Barriers to Childhood asthma care in Sub-Saharan Africa: a multi-country qualitative study with children and their caregivers.

**Participant selection**		
10. Sampling	How were participants selected? e.g. purposive, convenience, consecutive, snowball	Children and their caregivers were recruited initially as part of the ACACIA dataset. A Breathing Survey developed from questions in the Global Asthma Network (GAN) questionnaire **(Supplementary file 1)**was used to identify participants between 12 and 14 years with asthma symptoms or an asthma diagnosis.[22] Focus group participants (both children and their caregivers) were recruited using purposive sampling and categorised into four groups; children with a doctor-diagnosis of asthma (CAD) and their parents/caregivers (PAD), children with severe wheezing symptoms without a doctor-diagnosis of asthma (CND) and their parents/caregivers (PND), with FGDs held separately for each category<br>**Page 4 Study population**
11. Method of approach, mail, email	How were participants approached? e.g. face-to-face, telephone, mail, email	Participants were approached face to face. Focus groups were conducted face-to-face, with adherence to in-country-specific COVID-19 regulations.<br>**Page 4, Data collection , first sentence**
12. Sample size	How many participants were in the study?	A total of 136 children were included and 120 caregivers<br>**Page 5 , results and Table 1**
13. Non-participation	How many people refused to participate or dropped out? Reasons?	We did not keep data on the refusals or non-participation across countries<br>**Page 4 , Study population**
**Setting**		
14. Setting of data collection	Where was the data collected? e.g. home, clinic, workplace	At schools where the larger study was being carried out<br>**Page 4, Study setting , first sentence**
15. Presence of non-participants	Was anyone else present besides the participants and researchers?	Yes teachers. However the teachers did not join the children or caregivers FGDs. We conducted separate teachers FGDs<br>**Page 4 Study population**
**Data collection**		
17. Interview guide	Were questions, prompts, guides provided by the authors? Was it pilot tested?	Discussions lasted between 40-60 minutes and followed a predefined question guide specific to each focus group category. The discussion guides were developed after multiple iterations by the ACACIA core study team and informed by the study aims and literature review on barriers to asthma care. **(Supplementary file 2)**<br>**Page 4, data collection**

For peer review only - http://bmjopen.bmj.com/site/about/guidelines.xhtml

COREQ Qualitative research checklist :
Barriers to Childhood asthma care in Sub-Saharan Africa: a multi-country qualitative study with children and their caregivers.

18. Repeat interviews carried out?	Were repeat interviews carried out? If yes, how many?	No repeat interviews were done<br>**Page 14 , strengths and limitations**
19. Audio/visual recording	Did the research use audio or visual recording to collect the data?	Focus groups were audio-recorded, transcribed verbatim and translated to English if needed in the country.<br>**Page 4 , data collection**
20. Field notes	Were field notes made during and/or after the interview or focus group?	Yes this was done in the various countries<br>**Page 4 , data collection**
21. Duration	What was the duration of the interviews or focus group?	Discussions lasted between 40-60 minutes and followed a predefined question guide specific to each focus group category.<br>**Page 4 , data collection**
22. Data saturation	Was data saturation discussed?	Yes at two points<br>1. Code book development: A reiterative process was followed to apply the codebook to the transcribed data and determine whether further codes emerged. When no new codes emerged, the codebook was assumed to be a valid representation of the data and finalised (Supplementary 3).<br>2. Data analysis: Major themes and subthemes were identified based on patterns and relationships noted between derived codes, and all investigators agreed upon these after multiple iterations.<br>**Page 5 , data analysis**
23. Transcripts returned	Were transcripts returned to participants for comment and/or correction?	No. We did not conduct member checks. We have listed this in the limitations<br>**Page 14**
**Domain 3: analysis and findings**		
**Data analysis**		
24. Number of data coders	How many data coders coded the data?	Ten members of the ACACIA research group from all the participating countries independently developed codes from the transcripts and then discussed the coding to develop a code book.[23,24]<br>**Page 5 , data analysis**
25. Description of the coding tree	Did authors provide a description of the coding tree?	Yes. We used an inductive analysis to examine the data.[23] Ten members of the ACACIA research group from all the participating countries independently developed codes from the transcripts and then discussed the coding to develop a code book.[23,24] At least two investigators reviewed each transcript. A reiterative process was followed to apply the codebook to the transcribed data and

For peer review only - http://bmjopen.bmj.com/site/about/guidelines.xhtml

COREQ Qualitative research checklist :
Barriers to Childhood asthma care in Sub-Saharan Africa: a multi-country qualitative study with children and their caregivers.

		determine whether further codes emerged. When no new codes emerged, the codebook was assumed to be a valid representation of the data and finalised **(Supplementary 3).** **Page 5 , data analysis**
26. Derivation of themes	Were themes identified in advance or derived from the data?	We used an inductive analysis to examine the data.[23] **Page 4 ,data analysis**
27. Software	What software, if applicable, was used to manage the data?	The transcripts taken during the discussion were used to analyse the data using the NVivo 1.6 software program. **Page 4 , data analysis**
28. Participant checking	Did participants provide feedback on the findings?	No. Noted as a limitation **Page 14**
**Reporting**		
29. Quotations presented	29. Quotations presented Were participant quotations presented to illustrate the themes / findings? Was each quotation identified? e.g. participant number	Yes .each quote is identified using the country origin and category of the FGD and group number e.g. (CAD, MW, Group1); (CAD, ZW, Group1) **Pages 5 and 7-10**
30. Data and findings consistent	Was there consistency between the data presented and the findings?	Yes we have attempted to present the data to support the findings. **Page 7-10**
31.Clarity of major themes	Were major themes clearly presented in the findings?	Yes the themes are presented clearly and tables are used to support this **Page 7 , Table 2 and Figure 1**
32. Clarity of minor themes	Is there a description of diverse cases or discussion of minor themes?	With the multiple countries a major aim of the research was to focus on common (cross cutting) themes. However nuanced themes specific to countries are noted<br><br>The themes that were common across countries were specifically identified and highlighted and where specific in-country views differed these were noted. **Page 5 ,2nd paragraph , data analysis**

For peer review only - http://bmjopen.bmj.com/site/about/guidelines.xhtml

BMJ Open

Barriers to Childhood asthma care in Sub-Saharan Africa: a multi-country qualitative study with children and their caregivers.

Journal:	*BMJ Open*
Manuscript ID	bmjopen-2022-070784.R2
Article Type:	Original research
Date Submitted by the Author:	03-Aug-2023
Complete List of Authors:	Naidoo, Kimesh; University of KwaZulu-Natal Nelson R Mandela School of Medicine, Paediatrics and Child health ; King Edward VIII Hospital<br>Dladla, Sindiswa; University of KwaZulu-Natal Nelson R Mandela School of Medicine, Department of Paediatrics and Child Health<br>Mphahlele, Reratilwe; University of KwaZulu-Natal Nelson R Mandela School of Medicine, Paediatrics and Child Health<br>Mosler, Gioia; Queen Mary University of London Faculty of Medicine and Dentistry, Centre for Genomics and Child Health<br>Muyemayema, S; University of Zimbabwe, Department of Primary Health Care Sciences<br>Ssemata, Andrew; Makerere University College of Health Sciences, Psychiatry<br>Mkutumula, E; College of Medicine, 6. Malawi Liverpool Wellcome Programme, Queen Elizabeth Central Hospital, College of Medicine, Chichiri, Malawi<br>Adeyeye, Olayinka; Lagos State University College of Medicine<br>Moyo, Melinda ; University of Zimbabwe, 3. Child and Adolescent Health Unit (CAHU), Department of Primary Health Care Sciences<br>Goodman, Y; Lagos State University; Lagos State University Teaching Hospital<br>Kuyinu, Y; Lagos State University College of Medicine; Lagos State University Teaching Hospital<br>Nantanda, Rebecca; Makerere University, Lung Institute, Makerere College of Health Sciences<br>Ticklay, Ismail; University of Zimbabwe College of Health Sciences, Paediatrics and Child Health; University of Zimbabwe College of Health Sciences, Medical School Parirenyatwa Hospital<br>HA, Mujuru; University of Zimbabwe, Department of Primary Health Care Sciences<br>Grigg, Jonathan; Queen Mary University of London, Paediatric Respiratory<br>Masekela, R ; University of KwaZulu-Natal College of Health Sciences, Paediatrics and Child Health
**Primary Subject Heading**:	Paediatrics
Secondary Subject Heading:	Respiratory medicine
Keywords:	Asthma < THORACIC MEDICINE, PAEDIATRICS, RESPIRATORY

	MEDICINE (see Thoracic Medicine), PUBLIC HEALTH

I, the Submitting Author has the right to grant and does grant on behalf of all authors of the Work (as defined in the below author licence), an exclusive licence and/or a non-exclusive licence for contributions from authors who are: i) UK Crown employees; ii) where BMJ has agreed a CC-BY licence shall apply, and/or iii) in accordance with the terms applicable for US Federal Government officers or employees acting as part of their official duties; on a worldwide, perpetual, irrevocable, royalty-free basis to BMJ Publishing Group Ltd ("BMJ") its licensees and where the relevant Journal is co-owned by BMJ to the co-owners of the Journal, to publish the Work in this journal and any other BMJ products and to exploit all rights, as set out in our licence.

The Submitting Author accepts and understands that any supply made under these terms is made by BMJ to the Submitting Author unless you are acting as an employee on behalf of your employer or a postgraduate student of an affiliated institution which is paying any applicable article publishing charge ("APC") for Open Access articles. Where the Submitting Author wishes to make the Work available on an Open Access basis (and intends to pay the relevant APC), the terms of reuse of such Open Access shall be governed by a Creative Commons licence – details of these licences and which Creative Commons licence will apply to this Work are set out in our licence referred to above.

Other than as permitted in any relevant BMJ Author's Self Archiving Policies, I confirm this Work has not been accepted for publication elsewhere, is not being considered for publication elsewhere and does not duplicate material already published. I confirm all authors consent to publication of this Work and authorise the granting of this licence.

For peer review only

Title: Barriers to Childhood asthma care in Sub-Saharan Africa: a multi-country qualitative study with children and their caregivers.

Authors:

Naidoo KL, PhD[1]; Dladla S[1]; Mphahlele REM[1]; Mosler G[2], PhD; Muyemayema S[3]; Ssemata AS[4 5]; Mkutumula E[6]; Adeyeye O[7 8]; Moyo M[3]; Goodman Y[7 8]; Kuyinu Y[7 8]; Nantanda R[9 10];; Ticklay I [3 11]; Mujuru HA[3]; Grigg J[2]* , PhD; Masekela R[1] , PhD*

1. Department of Paediatrics and Child Health, School of Clinical Medicine, College of Health Sciences, University of KwaZulu-Natal
2. Centre for Genomics and Child Health, Blizzard Institute, Barts and The London School of Medicine and Dentistry, Queen Mary University of London, London, UK
3. Child and Adolescent Health Unit (CAHU), Department of Primary Health Care Sciences, Faculty of Medicine and Health Sciences, University of Zimbabwe, Avondale, Harare, Zimbabwe
4. Department of Psychiatry, College of Health Sciences, Makerere University, Kampala, Uganda
5. Department of Global Health and Development, London School of Hygiene and Tropical Medicine, London, UK
6. Malawi Liverpool Wellcome Programme, Queen Elizabeth Central Hospital, College of Medicine, Chichiri , Malawi
7. Lagos State University College of Medicine Ikeja, Lagos, Nigeria
8. Lagos State University Teaching Hospital, Ikeja, Lagos, Nigeria
9. Makerere University Lung Institute, Makerere University College of Health Sciences, Kampala, Uganda
10. Department of Paediatrics and Child Health, Makerere University College of Health Sciences, Kampala, Uganda
11. Parirenyatwa Group of Hospitals, Causeway, Harare, Zimbabwe

*Joint senior authors

For peer review only

For peer review only - http://bmjopen.bmj.com/site/about/guidelines.xhtml

Corresponding author

Dr KL Naidoo

Department of Paediatrics and Child Health, School of Clinical Medicine, College of Health Sciences, University of KwaZulu-Natal, 719 Umbilo Road, Congella, Durban South Africa

Email: naidook9@ukzn.ac.za

Telephone: 027 312604350

Fax: 027312604388

Five Key words: paediatrics, asthma, adolescence, children, sub-Saharan Africa.

Word count: Main manuscript word count = 4052 (excluding title page, abstract, references, figures and tables)

For peer review only

For peer review only - http://bmjopen.bmj.com/site/about/guidelines.xhtml

ABSTRACT – word count = 296

Objectives

This study identifies barriers and provides recommendations to improve asthma care in children across sub-Saharan Africa, where qualitative data is lacking despite high rates.

Design

One of the aims of our National Institute for Health Research global health research group "Achieving Control of Asthma in Children in Africa" (ACACIA) was to use qualitative thematic analysis of transcribed audio recordings from focus group discussions (FGDs) to describe barriers to achieving good asthma control.

Setting

Schools in Blantyre (Malawi), Lagos (Nigeria), Durban (South Africa), Kampala (Uganda), and Harare (Zimbabwe).

Participants

Children (n=136), 12 to 14 years with either asthma symptoms or a diagnosis and their caregivers participated in 39 FGDs. All were recruited using asthma control questions from the Global Asthma Network survey.

Results

There were four key themes identified: (1) Poor understanding, (2) Difficulties experienced with being diagnosed, (3) Challenges with caring for children experiencing an acute asthma episode and (4) Sub-optimal uptake and use of prescribed medicines. An inadequate understanding of environmental triggers, a hesitancy in using metered dose inhalers and a preference for oral and alternate medications were identified as barriers. In addition, limited access to health care with delays in diagnosis and an inability to cope with expected lifestyle changes was reported. Based on these findings, we recommend tailored education to promote access to and acceptance of metered dose inhalers, including advocating for access to a single therapeutic, preventative and treatment option. Furthermore, healthcare systems should have simpler diagnostic pathways and easier emergency access for asthma.

Conclusions

In a continent with rapidly increasing levels of poorly controlled asthma, we identified multiple barriers to achieving good asthma control along the trajectory of care. Exploration of these barriers reveals several generalisable recommendations that should modify asthma care plans and potentially transform asthma care in Africa.

Trial registration number 269211.

For peer review only

Key messages statements /strengths and limitations

- This is a first multi-country African qualitative study that used thematic analysis to identify barriers to asthma care.
- Perspectives of both adolescents and their caregivers on asthma diagnosis and management are included.
- Views of both children with a physician diagnosis of asthma and those with symptoms of asthma and not formally diagnosed are included.

Keywords: children, poor control, Childhood asthma, sub-Saharan Africa.

Funding statement

This research was funded by the National Institute for Health Research (NIHR) (Trial registration number 269211) using UK aid from the UK Government to support global health research.

Disclaimer The views expressed in this publication are those of the author(s) and not necessarily those of the NIHR or the UK Department of Health and Social Care (17/63/38).

INTRODUCTION

Globally, asthma is the most common non-communicable respiratory disease among children.(1) While the prevalence of asthma in African children is currently 14% and matches the global average, it is increasing in low-middle income countries (LMIC) with different trajectories suggested by the wide range of prevalence between countries, for example, Kenya at 15.8%, Nigeria 13.0%, and South Africa 20.3%.(1-4) The goal of asthma care is to optimise symptom control and maintain a good quality of life.(5, 6) Asthma control has been reported to be poor with the 2018 Global Asthma Report reporting high uncontrolled asthma prevalence rates across sub-Saharan Africa (sSA).(4) The determinants of poor asthma control in sSA are multifactorial including environmental factors which are certainly important and include rural to urban migration, early life urban residence, overcrowding, poorer air quality, lifestyle changes and diet.(6-9) Patient-related factors reported in African children include poor knowledge and symptom recognition, non-acceptance of inhaler treatment, older age and concurrent allergic rhinitis.(6,7) Health system factors include limited diagnosis of asthma, episodic management of asthma symptoms at the primary health care (PHC) level, and poor

For peer review only - http://bmjopen.bmj.com/site/about/guidelines.xhtml

access to inhaled corticosteroids.(5,6,9-12) However, to date, there is little qualitative data from children and young people on the determinants of asthma control in this region. Studies in high-income countries have found that children's and caregivers' health beliefs about asthma and poor relationships with healthcare providers (HCPs) can undermine asthma management.(13) Caregivers have also reported fear, feeling of powerlessness, uncertainty, and social tension related to their children who have asthma.(14-16) Studies from individual African countries have also identified physical, emotional and social burdens related to childhood asthma, and these have been postulated to affect non-adherence to recommended treatment regimens.(10,17-19)

Understanding the views of caregivers and affected children with doctor-diagnosed asthma and those with symptoms suggestive of asthma are needed to capture perspectives effectively. To accurately and efficiently determine strategies to improve asthma care, recommendations need to be identified and explored, that are derived from the perspectives of affected African children and their caregiver's concerns.(20) A richer, qualitative understanding of identified barriers to asthma care in African countries will assist with the development of relevant advocacy and policy interventions across the continent.(21) This study aimed to capture the views of children with asthma/asthma symptoms with and their caregivers, in sSA and to identify barriers to asthma diagnosis and care as well as interventions to improve asthma care across countries in Africa.

METHODS

Study design

This study was a component of the achieving control of the National Institute for Health Research (NIHR)funded global health research group's "Achieving control of asthma in children in Africa" (ACACIA), a larger observational cohort study across six African countries; Ghana, Malawi, Nigeria, South Africa, Uganda and Zimbabwe.(22) The ACACIA project aims to determine the burden of asthma in school-going children by assessing asthma control in children between 12 and 14 years with symptoms compatible with asthma or a doctor-diagnosis of asthma to assess symptom severity and asthma control. As a separate study, we conducted focus group discussions (FGDs) with children with an asthma diagnosis or symptoms suggestive of asthma and their caregivers to obtain their perspectives on the barriers to managing asthma. The study plan, procedures and methods were standardised across all countries.(22)

For peer review only - http://bmjopen.bmj.com/site/about/guidelines.xhtml

Study population

Children and their caregivers were recruited initially as part of the ACACIA dataset. A Breathing Survey developed from questions in the Global Asthma Network (GAN) questionnaire (Supplementary file 1) was used to identify participants between 12 and 14 years with asthma symptoms or an asthma diagnosis.(22) Focus group participants (both children and their caregivers) were recruited using purposive sampling and categorised into four groups; children with a doctor-diagnosis of asthma (CAD) and their parents/caregivers (PAD), children with severe wheezing symptoms without a doctor-diagnosis of asthma (CND) and their parents/caregivers (PND), with FGDs held separately for each category. The aim of this group categorisation was to ensure views of both children and caregivers, as well as those with and without a formal asthma diagnosis, were adequately sampled.

Study setting

The FGDs were held between 1 November 2020 and 30 June 2021 in schools in cities across countries: Blantyre, (Malawi); Lagos, (Nigeria); Durban, (South Africa); Kampala, (Uganda); and Harare, (Zimbabwe). Data from Ghana were not included for analysis due to challenges with COVID-19 restrictions in that country that impacted data collection. The in-country team of researchers and fieldworkers facilitated the focus group discussions.

Data collection

Focus groups were conducted face-to-face, with adherence to in-country-specific COVID-19 regulations with at least 1-2 FGD/s per category per country. All FGDs were moderated by at least two facilitators, one of whom was a study investigator at each site. FGDs took place in either English and /or isiZulu (SA), Shona (Zimbabwe) or Chichewa (Malawi). Other centres only used English. Focus groups were audio-recorded, transcribed verbatim and translated to English if needed in the country. Discussions lasted between 40-60 minutes and followed a predefined question guide specific to each focus group category. The discussion guides were developed after multiple iterations by the ACACIA core study team and informed by the study aims and literature review on barriers to asthma care. (Supplementary file 2)

Data analysis

The transcripts taken during the discussion were used to analyse the data using the NVivo 1.6 software program. We used an inductive analysis to examine the data.(23) Ten members of the

For peer review only - http://bmjopen.bmj.com/site/about/guidelines.xhtml

ACACIA research group from all the participating countries independently developed codes from the transcripts and then discussed the coding to develop a code book.(23,24) At least two investigators reviewed each transcript. A reiterative process was followed to apply the codebook to the transcribed data and determine whether further codes emerged. When no new codes emerged, the codebook was assumed to be a valid representation of the data and finalised (Supplementary 3).

Braun and Clarke's six-step thematic analysis was then used as the analytic framework to identify the patterns and themes within the coded data. (25) Major themes and subthemes were identified based on those commonly occurring codes in all countries. These sub-themes were highlighted and grouped into major themes based on commonly occurring patterns and relationships noted between the identified codes. All investigators reached consensus on these major themes and grouping of sub-themes after multiple iterations. Quotes chosen to illustrate the themes derived from the data were specifically selected only if they resonated across all countries.

Patient and Public Involvement

The focus groups were part of the wider ACACIA study and its patient and public involvement .(22) The focus groups set up, especially for children, was developed at each site with the local school staff, to provide a friendly and safe experience in a familiar environment. We tested the questions used in the focus group discussion at one of our sites (Nigeria) before other sites. Patients were involved at several stages of the ACACIA study, but not directly for the recruitment and conduct of the focus group element. A summary of all ACACIA study results will be disseminated to the participating schools, who will communicate it to students and parents

ETHICS

Ethical approval was granted at all the study sites: Malawi, COMREC Reference Number: COMREC P.10/18/2494;Nigeria, LREC 06/10/1084; Uganda, MHREC 1514, and UNCST SS 4940;Zimbabwe,MRCZ/A/2415;South Africa, BREC Ref No.: BF002/19.Participants provided written informed consent as well as informed caregiver (parent /guardian) consent and child assent in accordance with both country/local regulations and United Kingdom (UK)

For peer review only - http://bmjopen.bmj.com/site/about/guidelines.xhtml

ethics regulations **:**Trial registration number 269211.Data was de-identified and stored on a password protected computer with access only to researchers.

RESULTS

Description of the focus groups conducted

The data from 39 transcribed FGDs reflecting 256 participants across five countries were analysed. South Africa held 15 FGDs compared with 4-6 in all other countries. This was due to more parents and children who indicated willingness to participate in FDGs in SA during the COVID.-19 pandemic than all other countries. Table 1 indicates the breakdown of these groups per category and country. A total of 136 children were included with CAD 45.6% (n=62) and CND 54.4% (n=74). Of the 120 caregivers, they included PAD 42.5% (n=51) and PND 57.5% (n=69).

Table 1: Summary of children with asthma or asthma symptoms and their caregivers who participated in focus group discussions (n=256).

For peer review only - http://bmjopen.bmj.com/site/about/guidelines.xhtml

Summary of findings

Table 2 lists the subthemes and themes identified and Figure 1 shows the relationships. There were four major themes identified which were grouped into: (1) Poor understanding of Asthma, (2) Difficulties experienced with being diagnosed with Asthma, (3) Challenges with caring for children experiencing an acute asthma episode and (4) Sub-optimal uptake and use of prescribed asthma medicines .

Table 2. Major themes and subthemes identified.

Figure 1 .Relationships of the major themes and subthemes identified.

Poor understanding of asthma

This theme related to perspectives on how caregivers and children defined asthma and what was considered the cause of asthma. We could not ascertain differences in knowledge amongst caregivers of those with and without a diagnosis of asthma. We did find a distinction in how caregivers and children understood the causation of asthma. Children expressed fewer opinions on the familial or genetic nature of asthma than their caregivers. In all groups, there was a poor understanding of how environmental triggers and other factors such as prematurity and genetic predisposition contribute to the development of asthma.

> *There is dirt of some kind that interrupts ones breathing in their lungs so when air is meant to come in then you'll find that it's this dirt that is blocking the inhalation and exhalation of air.* (PND ZA, Group 6)

Difficulties experienced with being diagnosed with asthma

This theme related to perspectives being expressed about delays in being diagnosed, reflected predominantly in the groups with a formal doctor-diagnosis. Specific concerns related to a perceived reluctance of health care workers in making an asthma diagnosis with resulting frustration these delays cause.

> *I think that symptoms start with cough and flu. We waste a lot of time and yet the actual disease is not treated. So, after you have given that dose of cough and flu, when you go back, the doctor will tell you that is an infection yet it is asthma* (PAD, UG, and Group2)

In addition, this theme reflected on the emotional distress felt with disruptions to lifestyles due to acute asthmatic episodes. Both children and caregivers indicated displeasure about the disruption in activities of normal daily life after being diagnosed with asthma. These included

For peer review only - http://bmjopen.bmj.com/site/about/guidelines.xhtml

disruptions to school attendance, participation in sports and the child's share of household chores. Children expressed emotions of sadness and loss whilst caregivers expressed anxiety related to the disruption of their previous activities.

> *When am in an attack I can't do anything, I can't study, I can't go to class, I can't breathe properly, I can't focus, I can't understand. I can't be at school.* (CAD, MW, Group1)

> *Since we discovered she has asthma, my daughter is not exposed to a lot of things anymore. We don't allow her to sweep except if she's covered with a face mask before sweeping, we stopped her from drinking cold water and her room is cleaned properly, no rug, no cockroaches in the house, the fan is kept clean.* (PAD, NG, Group2)

Children also specifically expressed concerns about the stigma and discrimination they endured when their asthma diagnosis was known in the school environment. These reactions from significant others impacted negatively on children and caregivers leading to feelings of isolation and this promoted non-disclosure

> *It's also embarrassing because they all look at you funny and some start excluding you from games because they fear that you will have an attack. Some end up avoiding you forever and you remain with no one to play with.* (CAD, ZW, Group1)

> *I don't tell people that I am asthmatic because they will start looking at me all different.* (CAD, UG, Group2)

Challenges with caring for children experiencing an acute asthma episode

In all groups, identifying and avoiding various asthma triggers was seen as an important component in managing symptoms.

> *Simple solutions like make sure your surroundings are clean, check for dampness, in your bathroom or the ceilings and your walls … there are lots of things you can do to ensure that, the triggers are reduced.* (PND, ZA, Group2)

For peer review only - http://bmjopen.bmj.com/site/about/guidelines.xhtml

For caregivers, these avoidance strategies translated to altering the external environments of their children, whilst children focused more on how lifestyle changes impacted their daily lives. All groups articulated that they had difficulties with understanding what the various environmental triggers are and how to avoid them effectively.

> *I was outside and then my sister started sweeping the compound raising dust, the dust affected me and I started wheezing. I was then told to go inside the house and sleep.* (CAD, ZW, Group1)

The theme also includes caregivers' perspectives on their interactions the health systems, with challenges in accessing acute care when a child experiences an acute asthma episode
In these interactions, caregivers reflected on their frustrations, financial dependencies and vulnerabilities. These arose following the experiences of limited and often difficult access to health care during an acute asthmatic attack.

> *Government hospitals are useless … It is possible that the doctor's house is very near but for him to come is a challenge. Sometimes they will leave in the middle of their shift and you come with your child to the hospital and they tell you to wait … meanwhile your child's condition is worsening* (PAD, MW, and Group 1)

> *Apart from the hospital, I would also seek help from more accessible nurses in the neighbourhood or at home because these attacks usually happen when the hospital is too far and, in that moment, they may not be able to help you.* (PND, UG, Group1)

Sub-optimal uptake and use of prescribed asthma medicines

Across all groups, common perspectives on the role, availability and acceptance of various types of medications to treat asthma were identified. All groups reflected on relatively easier access to and acceptance of alternate and complementary asthma treatments than prescribed asthma medications.

For peer review only - http://bmjopen.bmj.com/site/about/guidelines.xhtml

Others told me to find a chameleon and leave it on flour for it to walk over, then to use the flour to make porridge to give the child. None of those things really worked! I have taken too much of these traditional medications! (PAD, MW, Group1)

I also use the inhaling smoke method. My mother does the same thing of putting marijuana seeds on hot coal and covers me with a blanket to inhale the smoke but it will be for five minutes tops and it clears my chest and I breathe better. (CND, ZW, Group1)

Juxtaposed to the acceptance of various alternate medications and treatment, there are widely held views that using and relying on inhalers should be met with caution. In addition, caregivers and children with asthma and no asthma diagnosis expressed various fears and myths about using prescribed inhalers. Concerns over possible addiction developing with the use of inhalers and fears of what could happen when inhalers are unavailable were expressed. In addition to these concerns, there is a clearly articulated preference for oral over inhaled medication by many.

*Okay a lot of people would tell us …we can use the inhalers but when I grow up it will build up in my body … also what if I get addicted to the inhaler and one day I have shortness of breath and don't have the inhaler? What will happen? (*CAD, MW, group2)

*The people from my village saw that I am using an inhaler, they asked me why I have started using it as if I don't know how such things end. But since last year when I started using the inhaler, everything is fine and we have not been admitted in the hospital since. I really had that mind-set that inhalers kill but since I started using them I saw that they are really helpful (*PAD, MW, and group1)

Barriers identified across countries

From the themes, we identified several barriers to asthma care across the five African countries, Table 3. Although there was some understanding of the role of genetic predisposition and allergen triggers, children and caregivers still expressed the need for more information, specifically regarding trigger avoidance. This included lifestyle changes that had to be made for families and disruption of normal family routines and chores. Reliance on the use of oral

For peer review only - http://bmjopen.bmj.com/site/about/guidelines.xhtml

and complementary medications was expressed with the stigma around the use of asthma inhaler therapies.

Table 3: Identified Barriers to asthma care across African countries

Discussion

Several barriers to asthma care that were common in all five African countries in this study were identified .The relationships of these barriers to optimal asthma care in Africa is highlighted specifically from the participants' perspectives. On further exploration, several cross-cutting recommendations were extrapolated from the analysis of these relationships of the barriers to caring for children with asthma and these were derived from the author's perspectives.

Inadequate understanding of environmental triggers and genetic predisposition in causing asthma

Asthma is a complex heterogeneous condition with multiple risk factors. The role of genetics, atopy and environment is complex even for experienced researchers. It is therefore not surprising that there was a poor societal understanding of the differences between cure, prevention and control, including confusion between trigger avoidance and symptom control using chronic treatments. Studies from SSA have noted that caregivers and children ascribe triggers with causation.(10,17) Thus, removing these 'offenders' equates to treatment/cure, lessening the perceived necessity for measures to prevent or control asthma.(10,17) These perspectives are identified in our study's findings of an inadequate understanding of environmental triggers, such as dust and cold weather. We speculate that this inadequate understanding translates to low 'necessity beliefs' towards the administration of chronic medications documented in Africa.(17) We thus further suggest that asthma education programs should clarify the role of triggers in the precipitation of asthma episodes and the need for medications for asthma control.

Hesitancy in health care workers making an asthma diagnosis resulting in delays and frustration

The delays in children receiving a doctor-diagnosis of asthma identified in our study can possibly be viewed within the context of poor symptom recognition, episodic management and low level of implementation of existing asthma guidelines by health care workers especially

For peer review only - http://bmjopen.bmj.com/site/about/guidelines.xhtml

noted at the primary health care level (PHC) in Africa.(17,26) A systematic review by Mphahlele RE et al. records low rates of asthma diagnosis (38.8%), prescribed medication (47.6%) and an increasing prevalence of severe uncontrolled asthma in African children.[6] Simplifying diagnostic algorithms for asthma to ensure ease of implementation of care in the African context should be thus viewed as a crucial need. We strongly advocate for this, based on our findings.

Difficulties experienced coping with adjustments in lifestyles

Children's perspectives on being self-conscious when using inhalers and thus appearing 'different' was an important theme in the present study and has been reported across both resource-developed and challenged countries.(13,19,26)The stigma associated with asthma and the subsequent reluctance to use inhalers in front of peers thus impacts self-management interventions deemed crucial to most asthma care programmes.(19) The use of child-generated strategies where peer interactions help break down social barriers has been suggested for asthma school education programmes.(27) It is relevant to African policy since asthma education programmes must compete with multiple health care messaging from common infectious diseases like human immunodeficiency virus, tuberculosis and malaria.(20) In this study, we identify peer acceptance as a major barrier in coping with asthma adjustments. We thus recommend that school-going children be sensitised to provide support, specifically with inhaler usage, to affected peers.

Another barrier identified was the difficulty delineating asthma triggers and the inability to alter their environment adequately. Studies from both sSA and elsewhere corroborate this finding, and the use of global checklists that can provide guidance on the range of triggers and viable environment modifications may be useful for inclusion, for example, in the World Health Organization Package of essential non-communicable disease interventions for primary health care (WHO PEN).(28)

Difficulties in obtaining hospital care when a child experiences an acute asthma episode

Poor access to asthma care across resource-constrained contexts, including Africa, has been noted as a general barrier, and our study corroborates this across all sampled countries.[7] This limited and often difficult access often leads to caregivers seeking alternate avenues for care and delaying appropriate care.(29,30) The WHO PEN attempts to improve asthma management at PHC sites; however, logistic challenges, including lack of essential medications at some sites, require interventions.(28) We specifically identify the need for access to oxygen,

For peer review only - http://bmjopen.bmj.com/site/about/guidelines.xhtml

nebulisers and monitoring, especially during acute episodes. The concern is that while PHC services may be accessible geographically, the cost of transport, waiting times, and loss of income also contribute to poor access. We thus advocate for improved access to both acute and chronic asthma care in primary health care settings.

Easier access to and acceptance of alternate and complementary asthma treatments compared with prescribed asthma medication

The acceptance and usage of traditional medicines and alternative treatments for asthma identified in this study have been previously ascribed to negative perceptions of prescribed medications and seen as inappropriate health-seeking behaviour that contributes to severe asthma.(17,26,30) In the current study, although ambivalence to prescribed medications contributes to poor inhaler use, widespread access to traditional and complementary medicine and indigenous knowledge systems also contributed to alternative treatments. We propose that the propagation of key programmes that advocate for and educate on inhaler therapies and devices should actively engage traditional health systems in the African context to understand better and embed asthma education in these systems.

Preference for prescribed oral asthma medication with fears and myths over using metered dose inhalers

Multiple studies have corroborated the poor usage and non-adherence to metered dose inhalers in childhood asthma as a major barrier to care.(11,17) This is further seen with the low levels of inhaled corticosteroids (6.7- 17%) and 73% of doctor-diagnosed/suspected asthma not using any inhaler documented.(6,31) Over-reliance on short-acting beta agonist (SABA) inhalers per se is also a barrier to appropriate care.(32) A preference for oral over inhaler-administered medications and widely held concerns that metered dose inhalers were addictive and harmful identified have been noted previously.(10,18) These factors are identified as leading reasons for inadequate use of metered dose inhalers.

With efficacy and safety studies advocating the use of single rapid onset (long-acting beta-agonist/inhaled corticosteroids (LABA/ICS) metered dose inhalers for maintenance, and reliever therapy there is a potential that this single therapeutic option could transform asthma care in sSA.(5,11) Advocacy and policy interventions to make this therapeutic option cheaper need urgent action.(17,11) We suggest that universal access to using a single therapeutic metered dose inhaler, where appropriate, be strongly advocated for in Africa and, in addition,

For peer review only - http://bmjopen.bmj.com/site/about/guidelines.xhtml

knowledge to dispel fears of using inhaler therapies and devices be included in asthma care plans.

Strengths and limitations of this study

This study presents a large qualitative dataset from multiple sites across Africa, reflecting a wide participant base. The large number of FGD were evenly spread over 4 countries with the exception of SA having more. The juxtaposition of children with and without a formal diagnosis and their caregivers generated rich data and new insights. We actively sought to identify common barriers across all sites, thus generating data generalisable to the SSA context. Transcription verbatim and using qualitative software to code the data enhanced the reliability. We attempted to ensure a fair and equitable coding team to decrease in-country biases, and this study thus reflects one of the few unique cross-country analyses from Africa.

Some loss of meaning may have occurred during the translation of African language quotes however, the data were analysed in the source language by members of the large international coding team who checked the semantic and context equivalence for translated quotes. We did not conduct member checks. Basic demographic data on participants was not provided except for enabling categorisation into the various groups.

We used an inductive approach with constant data comparisons and discussions to inform our understanding of the views expressed. We were aware of the risk of researcher bias in qualitative research and actively discussed reflexivity in the regular multidisciplinary team discussions, which aided analysis and informed interpretation. In addition, we have provided an explicit account for the derivation of the recommendations.

Conclusion

In a continent with rapidly increasing levels of severe uncontrolled asthma, multiple barriers to asthma diagnosis and management are identified along the entire trajectory of care. Exploration of these barriers reveals several broad, generalisable recommendations to modify asthma care plans that can mitigate challenges and potentially transform asthma care in Africa. Advocacy and tailored education are needed to promote access and acceptance for metered dose inhalers, including advocating for access to a single therapeutic, preventative and treatment modality. Healthcare systems require simpler diagnostic pathways and easier emergency access for childhood asthma across the continent.

DECLARATIONS

For peer review only - http://bmjopen.bmj.com/site/about/guidelines.xhtml

Acknowledgements

The authors wish to express our gratitude to all the children , caregivers and patient advisers who participated in this study.

Funding

This research was funded by the National Institute for Health Research (NIHR) , project number 17/63/38269211, trial registration number: 269211

Availability of data and materials

Anonymised data is available from the corresponding author upon request.

Authors' contributions

RM, JG, HAM, GM, and IT contributed to the study's conception. KLN, REM, SN, AS, MM, YG, YK, RN and OA performed the data collection, checking and coding the transcripts. KLN, REM, SN, AS, MM, YG, YK, RN and OA contributed to data analysis and interpretation. KLN, REM, SN and RM drafted the manuscript, and all authors provided critical revisions and editing. All authors reviewed the manuscript. RM and JG accept full responsibility for the work and the conduct of the study, had access to the data, and controlled the decision to publish

Disclaimer

The views and opinions expressed in this article are those of the authors and do not necessarily reflect the official policy or position of any affiliated agency of the authors.

Ethical consideration and consent to participate

This is contained in the manuscript.

Consent for publication

Not applicable.

Competing interests

The author(s) declare that they have no financial or personal relationship(s) that may have inappropriately influenced them in writing this article. The funder did not influence the results and compiling of the manuscript. JG reports personal fees from GSK, personal fees from Novartis, personal fees and a grant to QMUL from OM Pharma, personal fees and payment to QMUL from AstraZeneca, personal fees from Omron, outside the submitted work, and is supported by a NIHR Senior Investigator Award. RM reports consultancy and advisory board membership from AstraZeneca, Boehringer and Organon

References

1. Global Asthma Network. The Global Asthma Report 2018. http://globalasthmanetwork.org/Global%20Asthma%20Report%202018.pdf (accessed 03 October 2022)

For peer review only - http://bmjopen.bmj.com/site/about/guidelines.xhtml

2. Mortimer K, Reddel HK, Pitrez PM, et al. Asthma management in low and middle income countries: case for change. *Eur Respir J.* 2022;60(3):2103179. doi: 10.1183/13993003.03179-2021.
3. Adeloye D, Chan KY, Rudan I, Campbell H. An estimate of asthma prevalence in Africa: a systematic analysis. *Croat Med J.* 2013;54(6):519-31. doi: 10.3325/cmj.2013.54.519.
4. Asher MI, Rutter CE, Bissell K, et al.; Global Asthma Network Phase I Study Group. Worldwide trends in the burden of asthma symptoms in school-aged children: Global Asthma Network Phase I cross-sectional study. *Lancet* 2021;398(10311):1569-1580. doi: 10.1016/S0140-6736(21)01450-1.
5. Global Initiative for Asthma. Global Strategy for Asthma Management and Prevention, 2022: GINA 2022. https://ginasthma.org/wp-content/uploads/2022/07/GINA-Main-Report-2022-FINAL-22-07-01-WMS.pdf (accessed 03 October 2022)
6. Mphahlele RE, Kitchin O, Masekela R. Barriers and determinants of asthma control in children and children in Africa: a systematic review. *BMJ Open* 2021;11:e053100. doi: 10.1136/bmjopen-2021-053100.
7. Meghji J, Mortimer K, Agusti A, et al. Improving lung health in low-income and middle-income countries: from challenges to solutions. *Lancet* 2021 Mar 6;397(10277):928-940. doi: 10.1016/S0140-6736(21)00458-X.
8. Nagel G, Weinmayr G, Kleiner A, et al.; ISAAC Phase Two Study Group. Effect of diet on asthma and allergic sensitisation in the International Study on Allergies and Asthma in Childhood (ISAAC) Phase Two. *Thorax* 2010;65(6):516-522. doi: 10.1136/thx.2009.128256.
9. Rodriguez A, Vaca MG, Chico ME, et al. Rural to urban migration is associated with increased prevalence of childhood wheeze in a Latin-American City. *BMJ Open Respir Res* 2017; 4:e000205.
10. Mpairwe H, Tumwesige P, Namutebi M, et al. Asthma control and management among schoolchildren in urban Uganda: results from a cross-sectional study. *Wellcome Open Res* 2019;4:168. doi: 10.12688/wellcomeopenres.15460.1.
11. Masekela R, Mortimer K, Nantanda R, Lesosky M, Meme H, Devereux G, et al. Asthma care in sub-Saharan Africa: Mind the gap! *J Pan Afr Thorac Soc* 2022;3(2):59-62. doi:10.25259/JPATS_12_2022
12. García-Marcos L, Asher MI, Pearce N, et al.; Global Asthma Network Phase I Study Group. The burden of asthma, hay fever and eczema in children in 25 countries: GAN Phase I study. *Eur Respir J* 2022;60(3):2102866. doi: 10.1183/13993003.02866-2021.

For peer review only - http://bmjopen.bmj.com/site/about/guidelines.xhtml

13. Callery P, Milnes L, Verduyn C, Couriel J. Qualitative study of young people's and parents' beliefs about childhood asthma. *Br J Gen Pract* 2003;53(488):185-90. https://bjgp.org/content/bjgp/53/488/185.full.pdf (accessed 03 October 2022)

14. Lakhanpaul M, Culley L, Robertson N, et al. A qualitative study to identify parents' perceptions of and barriers to asthma management in children from South Asian and White British families. *BMC Pulm Med* 2017;17(1):126. doi: 10.1186/s12890-017-0464-9.

15. Chong YY, Leung D, Mak YW. When Control Exacerbates Distress: A Qualitative Study Exploring the Experiences of Hong Kong Chinese Parents in Caring for a Child with Asthma. *Int J Environ Res Public Health* 2018;15(7):1372. doi: 10.3390/ijerph15071372.

16. Chen SH, Huang JL, Yeh KW, Tsai YF. The Stress of Caring for Children With Asthma: A Qualitative Study of Primary Caregivers. *J Nurs Res* 2015;23(4):298-307. doi: 10.1097/JNR.0000000000000083.

17. Kassa E, Kebede RA, Habte BM. Perceptions towards childhood asthma and barriers to its management among patients, caregivers and healthcare providers: a qualitative study from Ethiopia. *BMC Pulm Med* 2022;22(1):184. doi: 10.1186/s12890-022-01984-2.

18. Barakat D, Rockers PC, Vian T, et al. Access to asthma medicines at the household level in eight counties of Kenya. *Int J Tuberc Lung Dis* 2018;22(5):585-590. doi: 10.5588/ijtld.17.0664.

19. Rose S, Paul C, Boyes A, et al. Stigma-related experiences in non-communicable respiratory diseases: A systematic review. *Chron Respir Dis* 2017;14(3):199-216. doi: 10.1177/1479972316680847.

20. Penza-Clyve SM, Mansell C, McQuaid EL. Why don't children take their asthma medications? A qualitative analysis of children's perspectives on adherence. *J Asthma* 2004;41(2):189-97. doi: 10.1081/jas-120026076.

21. Tefera BB, Kibret MA, Molla YB, et al. The interaction of healthcare service quality and community-based health insurance in Ethiopia. *PLoS One* 2021;16(8):e0256132. doi: 10.1371/journal.pone.0256132.

22. Mosler G, Oyenuga V, Addo-Yobo E, et al. Achieving Control of Asthma in Children in Africa (ACACIA): protocol of an observational study of children’s lung health in six sub-Saharan African countries. *BMJ Open* 2020;10:e035885. doi:10.1136/ bmjopen-2019-035885

For peer review only - http://bmjopen.bmj.com/site/about/guidelines.xhtml

23. Roberts K, Dowell A, Nie JB. Attempting rigour and replicability in thematic analysis of qualitative research data; a case study of codebook development. *BMC Med Res Methodol* 2019;19(1):66. doi: 10.1186/s12874-019-0707-y.
24. Boyatzis RE. Transforming qualitative information: thematic analysis and code development. Thousand oaks CA: SAGE Publications Inc; 1998. ISBN-13: 978-0761909613.
25. Braun V, Clarke V. Using Thematic Analysis in Psychology. Qualitative Research in Psychology 2006;3:77-101. doi: 10.1191/1478088706qp063oa.
26. Lion-Cachet HC, Musonda JMM, Omole OB. Severe asthma in South Africa: A literature review and management approach for primary care. *S Afr Fam Pract* 2021;63(1):e1-e10. Doi: 10.4102/safp.v63i1.5179.
27. Kelada L, Molloy CJ, Hibbert P, et al. Child and caregiver experiences and perceptions of asthma self-management. *NPJ Prim Care Respir Med* 2021;31(1):42. doi: 10.1038/s41533-021-00253-9.
28. World Health Organization. WHO package of essential non-communicable (PEN) disease interventions for primary health care. 07 September 2022. https://apps.who.int/iris/rest/bitstreams/1301957/retrieve (accessed 04 October 2022)
29. Searle A, Jago R, Henderson J, et al. Children's, parents' and health professionals' views on the management of childhood asthma: a qualitative study. *NPJ Prim Care Respir Med* 2017;27(1):53. doi: 10.1038/s41533-017-0053-7.
30. Joseph CL, Williams LK, Ownby DR, et al. Applying epidemiologic concepts of primary, secondary, and tertiary prevention to the elimination of racial disparities in asthma. *J Allergy Clin Immunol* 2006;117(2):233-40; quiz 241-2. doi: 10.1016/j.jaci.2005.11.004.
31. Mphahlele REM, Lesosky M, Masekela R. The prevalence, severity, and risk factors of asthma in South African children . Paper presented at The European Respiratory Society International Congress; PA3953; 2022 Sep 4-6; Barcelona, Spain.
32. Zeitouni MO, Al-Moamary MS, Coussa ML, Riachy M, Mahboub B, AlHuraish F, Zidan MH, Metwally MM, Aksu K, Yavuz E, Kalla IS, Chakaya J, Abdelmadjid S, Ghedira H. Challenges and recommendations for the management of asthma in the Middle East and Africa. *Ann Thorac Med* 2022;17(2):71-80. doi: 10.4103/atm.atm_469_21.

For peer review only - http://bmjopen.bmj.com/site/about/guidelines.xhtml

For peer review only

For peer review only - http://bmjopen.bmj.com/site/about/guidelines.xhtml

664 **Table 1: Summary of children with asthma or asthma symptoms and their caregivers who participated in focus group discussions(n=256).**

**Country (n=total number of focus groups)**	**Children n=136**				**Caregivers n=120**			
	Children with a doctor-diagnosis of Asthma (CAD)		Children with severe asthma symptoms but no diagnosis (CND)		Caregivers of children with a doctor-diagnosis of asthma (PAD)		Caregivers of children with severe asthma symptoms but no diagnosis. (PND)	
	Focus group (n)	Number of participants	Focus group (n)	Number of participants	Focus group (n)	Number of participants	Focus group (n)	Number of participants
**Malawi, MW (n=4)**	2	8	1	8	1	6	0	0
**Nigeria, NG(n=7)**	2	14	2	12	2	12	1	8
**South Africa, ZA (n=15)**	3	19	3	29	3	21	6	41
**Uganda, UG(n=7)**	2	12	1	6	2	12	2	19
**Zimbabwe, ZW(n=6)**	1	9	3	19	0	0	2	10
**Total participants (n=256)**	**10**	**62**	**10**	**74**	**8**	**51**	**11**	**69**

665

666

667

For peer review only - http://bmjopen.bmj.com/site/about/guidelines.xhtml

Table 2 : Major themes and subthemes and their relationships

**Major themes**	Poor understanding of Asthma	Difficulties experienced with being diagnosed with Asthma	Challenges with caring for children experiencing an acute asthma episode	Sub-optimal uptake and use of prescribed asthma medicines
**Subthemes**	• Confusion about asthma definitions<br>• Lack of clarity about what causes asthma	• Concerns about delays in getting an asthma diagnosis<br>• Disruptions in child's life on being diagnosed with asthma<br>• Negative responses from significant others to child's asthma diagnosis	• Confusion around triggers of an acute asthma episode<br>• Lack of knowledge on recognising and responding to an acute asthma episode<br>• Difficulties in access to hospital care when a child experiences an acute asthma episode	• Preferences for trying alternative/complementary treatments<br>• Poor acceptance with using inhalers

For peer review only

For peer review only

For peer review only - http://bmjopen.bmj.com/site/about/guidelines.xhtml

677 **Table 3: Identified Barriers to asthma care across African countries**

678

Barriers to asthma care across the African countries
1. Inadequate understanding of environmental triggers and causes of asthma impact on optimal, holistic asthma care<br>2. Reluctance in health care workers to make an asthma diagnosis results in delays in diagnosing asthma<br>3. Difficulties experienced coping with adjustments in lifestyles required with asthma hamper prevention of acute episodes<br>4. Limited access to health care during an acute asthmatic episode<br>5. Preferred usage of alternate and complementary asthma treatments delays uptake of prescribed asthma medication<br>6. Preferences in using oral asthma medication delays early adoption of inhaler medication<br>7. Fears and myths prevent uptake use of metered dose inhalers

679

680

681

For peer review only

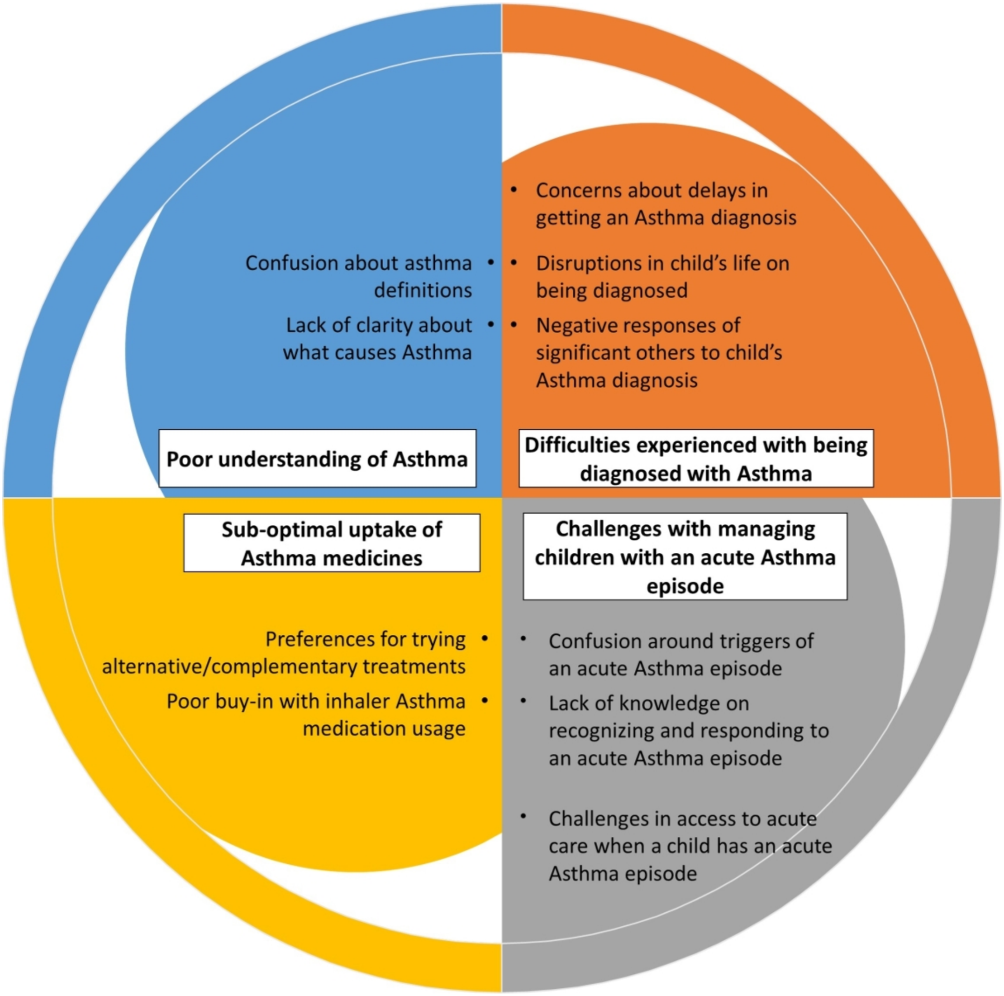

Figure 1 Relationship of the Major themes and sub-themes identified.

176x174mm (300 x 300 DPI)

acacia achieving control of asthma in children in africa

UNIVERSITY OF KWAZULU-NATAL INYUVESI YAKWAZULU-NATALI

ACACIA Breathing Survey

QR code label or Participant ID:

____

Introduction

We are a group of researchers interested in healthy breathing. In the questions below we will ask if you sometimes make a noise when you breathe, which is called wheezing. Wheezing is a whistling sound from the chest that some people make when they breathe.

Section 1: Personal Details

1. **What is your name?** ____

2. **Today's date:** DAY / MONTH / YEAR

3. **School:** ____

4. **Are you male or female?**

   ☐ Male

   ☐ Female

5. **How old are you?** _________

6. **What is your date of birth?** DAY / MONTH / YEAR

7. **Which school class or school year are you in?** ____________________________

8. **How would you describe your ethnicity?**

   ☐ Black

   ☐ White

   ☐ East Asian, such as Chinese, Japanese, or Korean

   ☐ South Asian, such as Indian, or Bangladeshi

   ☐ Mixed race, please specify: _______________________

   ☐ Other, please specify: ___________________________

For peer review only

acacia
achieving control of asthma in children in africa

UNIVERSITY OF KWAZULU-NATAL
INYUVESI YAKWAZULU-NATALI

Section 2: Breathing

1. **Have you had wheezing or whistling in the chest in the past 12 months?**

☐ Yes

☐ No

IF YOU HAVE ANSWERED “NO” PLEASE SKIP TO QUESTION 5

2. **How many attacks of wheezing have you had in the past 12 months?**

☐ None

☐ 1 to 3

☐ 4 to 12

☐ More than 12

3. **In the past 12 months, how often, on average, has your sleep been disturbed due to wheezing?**

☐ Never woken with wheezing

☐ Less than one night per week

☐ One or more nights per week

4. **In the past 12 months, has wheezing ever been severe enough to limit your speech to only one or two words at a time between breaths?**

☐ Yes

☐ No

5. **Have you ever had asthma?**

☐ Yes

☐ No

IF YOU HAVE ANSWERED YES,

6. **Was your asthma confirmed by a doctor?**

☐ Yes

☐ No

For peer review only

acacia
achieving control of asthma in children in africa

Page | 1

FGD 1: Children (12-14 yrs) with severe asthma and diagnosis of asthma

Before the discussion

1. Introduction
2. Game/warming up exercise

Discussion

1. Starter activity: Go around the room, everyone should finish the sentence: 'Asthma is…

   (The facilitators start, e.g. Asthma is… not easy/ hard to talk about/ limiting/ not a problem…)

2. How much do breathing problems affect what you do?
   - … At school?
   - … At home?
   - … Anywhere else?
3. If you have asthma medication, how do you take your asthma medications?
   - What helps you take your asthma medication?
   - What makes it difficult to take your asthma medication?
   - What do you dislike about your asthma medication?
   - Is there any other medication that you think helps you? For example traditional medication?
   - What are some of the ideas you have to make it easier to take your asthma medication?
4. How do you feel if you have difficulties breathing?
   - … When you are around your friends?
     - How do they react?
   - … When you are in class?
     - How does the teacher react?
5. *What do you believe people around you think about your asthma?*
   - Friends,
   - family,
   - teachers
   - What do you tell people about your asthma? And who do you talk to?

For peer review only

For peer review only - http://bmjopen.bmj.com/site/about/guidelines.xhtml

For peer review only

For peer review only - http://bmjopen.bmj.com/site/about/guidelines.xhtml

FGD 2: Children (12-14yrs) with severe asthma and no diagnosis

Before the discussion:

1. Introduction
2. Game/warming up exercise

Discussion:

1. What would you do if you have difficulties breathing?
    - Can you think of anything that might make it easier to breathe?
    - Do you think there is anything that might make it worse?
2. Have you ever taken any medication or remedies for your breathing difficulty?
    - Tell me about it.
3. How much do breathing problems affect what you do?
    - … At school?
    - … At home?
    - … Anywhere else?
4. How do you feel if you have difficulties breathing...?
    - … When you are around your friends?
        - How do you think they would react?
    - … When you are in class?
        - How has the teacher reacted?
5. Have you heard of asthma?
    - What do you think does it mean to have asthma?
6. What would you think if a very good friend suddenly tells you that he/she has asthma?

For peer review only

For peer review only - http://bmjopen.bmj.com/site/about/guidelines.xhtml

FGD 3: Parents of children with diagnosed asthma

Before the discussion

1. Introduction
2. Warm up exercise / tea & cookies
3. Short questionnaire
    - Knowledge questions (symptoms of asthma/triggers etc.)
    - Questions about beliefs around asthma (e.g. How much support do you think a person with asthma needs? A lot,…, none)
    - Where do you go to seek help?

Discussion

1. Asthma is… (finish sentence)
2. Tell us about your experience with asthma?
    - Tell us about your experience with healthcare
        - diagnosis
    - … Experience with asthma treatment (inhalers, alternative treatment methods etc.)
    - Tell us about any impact the asthma diagnosis had for you and your family
        - Financially
        - Socially
3. Can you tell us what makes it difficult for your child to take the asthma medication?
4. What do you think it means for your child to have asthma?
    - Would you have any special concerns about your child because of their asthma?
        - Would you think there is anything that an asthmatic child should avoid?
5. Do you have any examples of what makes life with asthma difficult for your child?
6. Is there anything that helps your child to live with asthma, apart from medication?

FGD 4: Parents of children without asthma

Before the discussion

1. Introduction
2. Warm up exercise/ tea & cookies
3. Short questionnaire
    - Knowledge questions (symptoms of asthma/triggers etc.)

For peer review only - http://bmjopen.bmj.com/site/about/guidelines.xhtml

- Questions about beliefs around asthma (e.g. How much support do you think a person with asthma needs? A lot,…, none)
- If your child had problems to breathe sometimes, where would you go to seek help?

Discussion

1. How would you describe asthma?
   - Tell us about your experience with asthma (Do you know anyone with asthma?)
   - If you hear that your child's friend had asthma, what would you say to your child?
2. If you are told that your child has asthma,
   - How would you react?
   - What would you tell your child?
   - Where would you seek help?
   - How would you manage?
   - How would you feel?
3. Why do you think someone might be worried about asthma?
   - Who do you think might be affected by asthma?

For peer review only

For peer review only - http://bmjopen.bmj.com/site/about/guidelines.xhtml

FGD 1: Children (12-14 yrs.) with severe asthma and diagnosis of asthma		
**Code**	**Definition**	**Purpose/meaning of code**
1. Definition of asthma	Participant defines asthma	Indicates participant's knowledge of what asthma is<br>Indicates Knowledge
2. Effects of breathing difficulties	Participant describes their own experience of how their asthma affects them when they are in various environments such as school, home and anywhere else	Participant indicates how much their breathing problems alter their activities in different environments.
3. Positive experience with asthma medication	participants describe what is pleasing to them about their medications and why they use them	participant describe what improves their symptoms<br>what makes it easy for them to use the medications
4. Negative experience with asthma medication	Participant describe what is not pleasing about their medication and why they do not like to use them.	What make is difficult to take their medication, can be taste, smell, stigma attached to taking medication in public
5. Alternative medication	Participant indicates and describes an experience of taking any alternative medication for their asthma	Indicate alternative medication/methods or idea to relieve their symptoms. Can be at school, home or anywhere else
6. reaction of friends to an asthma attack	How their friends have reacted to them while having an asthma attack and how they felt about their friend's reaction	Determine if there are perceived stigma limiting expression of symptoms. Determine the extent to which peers could provide support or otherwise
7. Reaction to having an asthmatic attack teachers	Participant recalls or describes how their teachers reacted when they were having an asthma attack and how they felt when that was happening	How they perceive getting help from the teachers. Finding out the aid that is offered to the learner and how teachers respond/react to asthmatic learner
8. Perception of asthma from friends	Describe whether friends are aware that they have asthma or not? Describe how the friends feel about having an asthma sufferer as a friend	Describe friends' reaction to them having asthma.<br>how has the relationship being impacted
9. Perception of asthma from family	Participants describe what they family think or feel about their asthma	Are they treated differently in their family because of their asthma? How does their family react when they exhibit symptoms? Have there been any changes to the household since their diagnosis?
10. Perceptions of asthma from teachers	Participants describe what their teachers feel about them having asthma	Do their teachers know they have asthma? What kind of assistance do they receive from the teachers to reduce risk of developing symptoms in school?<br>What kind of assistance do they receive when they exhibit asthma symptoms?
11. Personal perceptions about your asthma	Participants describe how they feel about their own asthma, what are their experiences around it	We are trying to find out how they feel or think about their asthma, what are their experiences around it, good or bad

FGD 2: Children (12-14yrs) with severe asthma and no diagnosis		
**Code**	**Definition**	**Purpose/meaning of code**
1. Easing breathing difficulties	Participants describe what makes it easier for them to breath when they have trouble breathing	What are their coping mechanisms, what do they do in order for their symptoms not to get worse
2. Exacerbations of breathing difficulties	Participants describe what makes it worse for them when they have difficulties breathing	identify things that make their symptom worse
3. Medications	Participants indicate if they have taken any medications for their symptoms or any alternative methods to help them with their symptoms. Identify medication use : type	Finding out what medications and remedies they have used. Describe what is done to bring relief
4. Effects of experiencing breathing problem	Participant describes their experience of how their asthma affects them when they are in various environments such as school, home and anywhere else	description of experience of asthma symptoms- home, school etc.
5. Reaction from friends	Participant recalls and describe how their friends reacted when they were having an asthma attack and how they felt when that was happening	How they perceive getting help from their peers. Finding out they experience any stigma from exhibiting breathing difficulties in front of their friends
6. Reaction from teachers	Participant recalls and describe how their teachers reacted when they were having an asthma attack and how they felt when that was happening	How they perceive the help or otherwise from school. describe what was done and the teacher's reaction
7. Perceptions of having breathing problems /asthma	Participant perception of what asthma is and how they will react if a friend have asthma	Definition of asthma and perception of it

For peer review only

FGD 3: Parents of children with diagnosed asthma		
**Code**	**Definition**	**Purpose/meaning of code**
1.Definition of asthma	Participant defines asthma/ describe asthma symptoms	Indicates participant's knowledge of what asthma is
2. Response to initial asthma diagnosis	Participant describes their experience when their child was diagnosed with asthma	Describe parents' initial reaction to asthma diagnosis
3.Experience with healthcare with regards asthma		Journey to diagnosis of asthma in healthcare
4.Positive experiences asthma medication	Participants describes their experience with asthma treatment	describe parents knowledge about asthma treatment and support provided when child was diagnosed
5.Negative experience with asthma medication	Participant describes negative aspects of asthma medication. What they don't like about the medication	What make is difficult to for their children to take the medication, can be taste, smell, stigma attached to taking medication in public
6.Financial impact of asthma diagnosis	Cost of medication, availability of medication at their local healthcare facility, fear of side effects	determine the financial implication (direct and indirect cost of asthma care)
7.Social implications of the diagnosis	Social implications of the asthma diagnosis on their children and family	Try to find out the perceptions and attitude to asthma in the community they live in or perceptions from their family. Find out the if they have experienced stigma or support
8.Concerns around asthma	What it means for their child to have asthma. Their perceptions and worries around the diagnosis and care of asthma	How people would Perceive or treat their child, how the child would cope if not at home etc.
9.Enablers in asthma care of children	Participant describes what makes it easier for their child with an asthma diagnosis to be cared for	Parents perception of what will make life easier for a child with asthma
10. Difficulties of an asthma diagnosis	Participant describes what has been difficult since the asthma diagnosis	Parents perception of what has made life difficult for a child with asthma

For peer review only

FGD 4: Parents of children without asthma		
**Codes**	**Definition**	**Purpose/meaning code**
1. Knowledge of breathing problems in children	Participant recalls and describes their experience and knowledge around asthma	Try to find out their knowledge around asthma and if they know someone with asthma
2. Perception of asthma in other children	Their reactions if their child's friend had asthma	Try to find out their perception around asthma, fear, stigma etc.
3. Reaction if their child had asthma	Their reaction if their child was diagnosed with asthma	Try to find out their perception and feeling about asthma
4. communicating with child on asthma	How would they tell their child about the diagnosis	Try to find out their knowledge around asthma or what resources they would use to tell the child more about asthma. How would they communicate asthma information to their child
5. Seeking help if their child had asthma	Where would they go to seek help for asthma, asthma care, treatment and information	Try to find out if they have access to resources that can help them and their child. What type kind of resources are available to them in case of emergencies<br>Where they would seek help i.e. from people, places
6. Manage asthma care/Breathing problems	Participant describe what they would do differently if their child was diagnosed with asthma and how they would care for them	How would they manage the care of their child who has asthma- things they would do in terms of care
7. Positive Feelings on asthma	Their perceptions around asthma	any positive feeling described
8. Negative feelings /Concerns around asthma	Participant describes their concerns about asthma or an asthmatic's concern about asthma	Describes such feeling as fear, afraid of stigma, afraid of financial burden, burden of visiting hospital frequently, fear for sudden asthma attacks or what the child may not be able to do.

For peer review only

For peer review only

COREQ Qualitative research checklist :
Barriers to Childhood asthma care in Sub-Saharan Africa: a multi-country qualitative study with children and their caregivers.

No Item Guide	questions/description	
**Domain 1:**	**Research team and reflexivity**	NB We attempted to ensure a fair and equitable coding team to decrease in-country biases **Page 5, data analysis 2nd sentence**<br>We used an inductive approach with constant data comparisons and discussions to inform our understanding of the views expressed. We were aware of the risk of researcher bias in qualitative research and actively discussed reflexivity in the regular multidisciplinary team discussions, which aided analysis and informed interpretation. **Page 5 , data analysis 2nd paragraph**
**Personal Characteristics**		
1. Interviewer/facilitator?	Which author/s conducted the interview or focus group?	All focus group discussions (FGDs) were moderated by at least two facilitators, one of whom was a study investigator at each site. **Page 4 , Data collection**<br>Reference is provided for the protocol **Reference 22**
2. Credentials	What were the researcher's credentials? E.g. PhD, MD	"one of whom was a study investigator at each site', Research project has a list of credentials of all investigators- reference for the published protocol included<br>**Page 4 ,data collection 2nd sentence and full protocol reference 22**
3. Occupation	What was their occupation at the time of the study?	Research project has a list of credentials of all investigators- reference for the published protocol included<br>**Page 4 ,data collection 2nd sentence and full protocol reference 22**,
4. Gender	Was the researcher male or female?	All genders .multiple researchers<br>**Page 4**
5. Experience and training	What experience or training did the researcher have?	Research project has a list of credentials of all investigators- reference for the published protocol included<br>**Page 4 and Reference 22 Protocol has details of the greater research project**
**Relationship with participants**		
6. Relationship established Was a relationship established prior to study commencement?	Was a relationship established prior to study commencement?	The details of the larger study are provided. 'Children and their caregivers were recruited initially as part of the ACACIA dataset'<br>**Page 4 Study population and reference 22**

For peer review only - http://bmjopen.bmj.com/site/about/guidelines.xhtml

COREQ Qualitative research checklist :
Barriers to Childhood asthma care in Sub-Saharan Africa: a multi-country qualitative study with children and their caregivers.

7. Participant knowledge of the interviewer	What did the participants know about the researcher? e.g. personal goals, reasons for doing the	All moderators introduced themselves at all FGDs. The standardised interview guide included a formal introductions component<br>Participants provided written informed consent as well as informed caregiver (parent /guardian) consent and child assent in accordance with both country/local regulations and United Kingdom (UK) ethics regulations.- this process provided information on the goals of both the larger project and the FGDs<br>**Page 4 data collection**
8. Interviewer characteristics,	What characteristics were reported about the interviewer/facilitator? e.g. Bias, assumptions, reasons and interests in the research topic	There were multiple facilitators across all countries. Focus groups were conducted face-to-face, with adherence to in-country-specific COVID-19 regulations. All FGDs were moderated by at least two facilitators, one of whom was a study investigator at each site. **Page 4 , data collection paragraph**<br>Participants provided written informed consent as well as informed caregiver (parent /guardian) consent and child assent in accordance with both country/local regulations and United Kingdom (UK) ethics regulations.- this process provided information on the goals of both the larger project and the FGDs<br>**Page 5 , ethics paragraph**
**Domain 2: study design**		
**Theoretical framework**		
9. Methodological orientation and theory	What methodological orientation was stated to underpin the study? e.g. grounded theory, discourse analysis, ethnography, phenomenology, content analysis	This study was across 6 countries and used an inductive analysis to examine the data.[23] The methodological orientation was largely content analysis using a codebook analysis due to the large number of transcripts .We have provided references for the process we followed with the code book development<br>Ten members of the ACACIA research group from all the participating countries independently developed codes from the transcripts and then discussed the coding to develop a code book.[23,24] At least two investigators reviewed each transcript. A reiterative process was followed to apply the codebook to the transcribed data and determine whether further codes emerged. When no new codes emerged, the codebook was assumed to be a valid representation of the data and finalised<br>**Page 5 , data analysis**

For peer review only - http://bmjopen.bmj.com/site/about/guidelines.xhtml

COREQ Qualitative research checklist :
Barriers to Childhood asthma care in Sub-Saharan Africa: a multi-country qualitative study with children and their caregivers.

**Participant selection**		
10. Sampling	How were participants selected? e.g. purposive, convenience, consecutive, snowball	Children and their caregivers were recruited initially as part of the ACACIA dataset. A Breathing Survey developed from questions in the Global Asthma Network (GAN) questionnaire **(Supplementary file 1)**was used to identify participants between 12 and 14 years with asthma symptoms or an asthma diagnosis.[22] Focus group participants (both children and their caregivers) were recruited using purposive sampling and categorised into four groups; children with a doctor-diagnosis of asthma (CAD) and their parents/caregivers (PAD), children with severe wheezing symptoms without a doctor-diagnosis of asthma (CND) and their parents/caregivers (PND), with FGDs held separately for each category<br>**Page 4 Study population**
11. Method of approach, mail, email	How were participants approached? e.g. face-to-face, telephone, mail, email	Participants were approached face to face. Focus groups were conducted face-to-face, with adherence to in-country-specific COVID-19 regulations.<br>**Page 4, Data collection , first sentence**
12. Sample size	How many participants were in the study?	A total of 136 children were included and 120 caregivers<br>**Page 5 , results and Table 1**
13. Non-participation	How many people refused to participate or dropped out? Reasons?	We did not keep data on the refusals or non-participation across countries<br>**Page 4 , Study population**
**Setting**		
14. Setting of data collection	Where was the data collected? e.g. home, clinic, workplace	At schools where the larger study was being carried out<br>**Page 4, Study setting , first sentence**
15. Presence of non-participants	Was anyone else present besides the participants and researchers?	Yes teachers. However the teachers did not join the children or caregivers FGDs. We conducted separate teachers FGDs<br>**Page 4 Study population**
**Data collection**		
17. Interview guide	Were questions, prompts, guides provided by the authors? Was it pilot tested?	Discussions lasted between 40-60 minutes and followed a predefined question guide specific to each focus group category. The discussion guides were developed after multiple iterations by the ACACIA core study team and informed by the study aims and literature review on barriers to asthma care. **(Supplementary file 2)**<br>**Page 4, data collection**

For peer review only - http://bmjopen.bmj.com/site/about/guidelines.xhtml

COREQ Qualitative research checklist :
Barriers to Childhood asthma care in Sub-Saharan Africa: a multi-country qualitative study with children and their caregivers.

18. Repeat interviews carried out?	Were repeat interviews carried out? If yes, how many?	No repeat interviews were done<br>**Page 14 , strengths and limitations**
19. Audio/visual recording	Did the research use audio or visual recording to collect the data?	Focus groups were audio-recorded, transcribed verbatim and translated to English if needed in the country.<br>**Page 4 , data collection**
20. Field notes	Were field notes made during and/or after the interview or focus group?	Yes this was done in the various countries<br>**Page 4 , data collection**
21. Duration	What was the duration of the interviews or focus group?	Discussions lasted between 40-60 minutes and followed a predefined question guide specific to each focus group category.<br>**Page 4 , data collection**
22. Data saturation	Was data saturation discussed?	Yes at two points<br>1. Code book development: A reiterative process was followed to apply the codebook to the transcribed data and determine whether further codes emerged. When no new codes emerged, the codebook was assumed to be a valid representation of the data and finalised (Supplementary 3).<br>2. Data analysis: Major themes and subthemes were identified based on patterns and relationships noted between derived codes, and all investigators agreed upon these after multiple iterations.<br>**Page 5 , data analysis**
23. Transcripts returned	Were transcripts returned to participants for comment and/or correction?	No. We did not conduct member checks. We have listed this in the limitations<br>**Page 14**
**Domain 3: analysis and findings**		
**Data analysis**		
24. Number of data coders	How many data coders coded the data?	Ten members of the ACACIA research group from all the participating countries independently developed codes from the transcripts and then discussed the coding to develop a code book.[23,24]<br>**Page 5 , data analysis**
25. Description of the coding tree	Did authors provide a description of the coding tree?	Yes. We used an inductive analysis to examine the data.[23] Ten members of the ACACIA research group from all the participating countries independently developed codes from the transcripts and then discussed the coding to develop a code book.[23,24] At least two investigators reviewed each transcript. A reiterative process was followed to apply the codebook to the transcribed data and

For peer review only - http://bmjopen.bmj.com/site/about/guidelines.xhtml

COREQ Qualitative research checklist :
Barriers to Childhood asthma care in Sub-Saharan Africa: a multi-country qualitative study with children and their caregivers.

		determine whether further codes emerged. When no new codes emerged, the codebook was assumed to be a valid representation of the data and finalised **(Supplementary 3).** **Page 5 , data analysis**
26. Derivation of themes	Were themes identified in advance or derived from the data?	We used an inductive analysis to examine the data.[23] **Page 4 ,data analysis**
27. Software	What software, if applicable, was used to manage the data?	The transcripts taken during the discussion were used to analyse the data using the NVivo 1.6 software program. **Page 4 , data analysis**
28. Participant checking	Did participants provide feedback on the findings?	No. Noted as a limitation **Page 14**
**Reporting**		
29. Quotations presented	29. Quotations presented Were participant quotations presented to illustrate the themes / findings? Was each quotation identified? e.g. participant number	Yes .each quote is identified using the country origin and category of the FGD and group number e.g. (CAD, MW, Group1); (CAD, ZW, Group1) **Pages 5 and 7-10**
30. Data and findings consistent	Was there consistency between the data presented and the findings?	Yes we have attempted to present the data to support the findings. **Page 7-10**
31.Clarity of major themes	Were major themes clearly presented in the findings?	Yes the themes are presented clearly and tables are used to support this **Page 7 , Table 2 and Figure 1**
32. Clarity of minor themes	Is there a description of diverse cases or discussion of minor themes?	With the multiple countries a major aim of the research was to focus on common (cross cutting) themes. However nuanced themes specific to countries are noted<br><br>The themes that were common across countries were specifically identified and highlighted and where specific in-country views differed these were noted. **Page 5 ,2nd paragraph , data analysis**

For peer review only
